# Spatio-temporal trends of the prevalence of *Plasmodium falciparum* antimalarial drug resistance markers across sub-Saharan Africa from 2010 to 2024: a systematic review and meta-analysis

**DOI:** 10.1186/s12879-026-14233-2

**Published:** 2026-09-03

**Authors:** Francis Emmanuel Towanou Bohissou, Guétawendé Job Wilfried Nassa, Juliana Inoue, Paul Sondo, Toussaint Rouamba, Joanna Yessinou, Jana Held, Halidou Tinto

**Affiliations:** 1 Institute of Tropical Medicine, University Hospital Tübingen, 72074 Tübingen, Germany; 2 Institut de Recherche En Sciences de la Santé (IRSS)/Clinical Research Unit of Nanoro (CRUN), Nanoro, Burkina Faso; 3 Centre de Recherche Entomologique de Cotonou (CREC), Cotonou, Bénin; 4 German Center for Infection Research (DZIF), Partner Site Tübingen, Tübingen, Germany; 5 Centre de Recherches Médicales de Lambaréné, (CERMEL), Lambaréné, Gabon

**Keywords:** *Plasmodium falciparum*, Antimalarial, Resistance markers, Sub-Saharan Africa, Systematic review

## Abstract

**Background:**

The emergence and subsequent spread of antimalarial drug resistance represent a significant threat to the efficacy of artemisinin-based combination therapies (ACTs) in sub-Saharan Africa. Multiple molecular markers have been implicated in resistance to artemisinin and its partner drugs. We conducted a systematic review and meta-analysis of studies published between 2010 and 2024 in sub-Saharan Africa.

**Methods:**

The protocol was registered in PROSPERO (CRD42023432718). Studies were identified through PubMed/MEDLINE, Web of Science, Scopus, Embase, and African Journal Online. Study selection, data extraction, and risk-of-bias assessment were performed independently by two reviewers in accordance with the PRISMA 2020 guidelines. Prevalence data for mutations in *PfKelch13*, *Pfcrt*, *Pfmdr1*, and *Pfdhfr/Pfdhps* were extracted and analysed using random-effects models, with pooled prevalence estimates generated using a generalized linear mixed model (GLMM) with logit transformation. Between-study heterogeneity was assessed using Cochran’s Q and I^2^ statistics.

**Results:**

A total of 172 studies on *PfKelch13*, 182 on *Pfcrt*, 196 on *Pfmdr1*, and 176 on *Pfdhfr/Pfdhps* were included. From 2014 to 2023, validated *PfKelch13* mutations exhibited marked spatial and temporal heterogeneity, with R622I, R561H, and A675V expanding primarily in East Africa, reaching prevalences of 10–25% in Rwanda, Uganda, and Tanzania. In Central Africa, emergence was observed in 2021 at low levels (below 5%), while West Africa reported only sporadic low-prevalence (below 1%) detections. In contrast, resistance to some partner drugs declined substantially across Africa, with *Pfcrt* K76T decreasing from 76% (95% CI: 49% − 91%; *I*^*2*^ = 97.9%) in 2010 to 9% (95% CI: 8.7% − 9.5%; *I*^*2*^ = 96.8%) in 2023 and *Pfmdr1* N86Y falling from 34% (95% CI: 26% − 43%; *I*^*2*^ = 93.5%) to zero (95% CI: 0% − 11%; *I*^*2*^ = 97.1%), with persistent regional heterogeneity. Certain markers of sulfadoxine–pyrimethamine (SP) resistance (*Pfdhfr* N51I, C59R, S108N and *Pfdhps* A437G) remained near fixation, whereas *Pfdhfr* I164L, *Pfdhps* K540E, and *Pfdhps* A581G showed pronounced spatial clustering, with the highest prevalence reported in East and parts of Central Africa.

**Conclusion:**

Overall, the temporal and regional patterns of antimalarial drug resistance molecular markers across sub-Saharan Africa underscore the need for sustained molecular surveillance tailored to local epidemiological trends of resistance markers.

**Registration:**

Protocol registered in the International Prospective Register of Systematic Reviews (PROSPERO; registration number CRD42023432718).

**Supplementary Information:**

The online version contains supplementary material available at https://doi.org/10.1186/s12879-026-14233-2.

## Background

Artemisinin-based combination therapies (ACTs) are the cornerstone of malaria treatment worldwide, particularly in Africa. Indeed, the widespread deployment of ACTs has substantially reduced malaria incidence and mortality, contributing to significant public health gains [1]. In 2023, control programs in endemic countries distributed approximately 235 million ACTs, 99% of which were delivered in sub-Saharan Africa. Nearly 145 million ACTs were distributed across five countries alone: Nigeria (40 million), Uganda (29.2 million), Zambia (27.8 million), the Democratic Republic of Congo (26.2 million), and Mozambique (21.5 million) [2]. ACTs used as first-line treatments combine an artemisinin derivative with a partner drug such as lumefantrine, mefloquine, amodiaquine, piperaquine, pyronaridine, or sulfadoxine-pyrimethamine (SP). These combinations leverage the rapid parasiticidal activity but short half-life of artemisinin derivatives alongside the slower-acting yet longer-lasting partner drugs. Artemether–lumefantrine (AL) is the most widely used ACT in endemic countries, followed by artesunate–amodiaquine (ASAQ) and dihydroartemisinin–piperaquine (DHAPPQ) [1].

Despite these successes, declining ACT efficacy, particularly for AL, has emerged as a major concern [3–5]. Central to this threat is artemisinin partial resistance, clinically defined as delayed parasite clearance after treatment with artemisinin-based monotherapy or ACT [6]. Remarkably, only one year after the World Health Organisation (WHO) endorsed universal ACT use in 2008, delayed parasite clearance, suggestive of artemisinin partial resistance, was reported along the Thailand–Cambodia border [7]. Over the subsequent decade, artemisinin partial resistance spread widely across the Greater Mekong subregion, extending from coastal Vietnam in the east to the Indian border in the west [8, 9]. Artemisinin partial resistance is strongly associated with mutations in the *P. falciparum Kelch13 (PfKelch13*) propeller domain. These mutations reduce the parasite sensitivity to artemisinin by impairing drug activation, enhancing antioxidant defences, limiting protein damage, and promoting DNA repair [10, 11]. The WHO classifies *PfKelch13* mutations as validated markers when clinical and laboratory evidence demonstrates their association with artemisinin partial resistance. Other mutations are categorised as candidate or associated markers when the available evidence is incomplete, indicating an association with either clinical resistance phenotypes or in vitro resistance, but not both [12]. According to the WHO, 13 mutations (F441I, N458Y, C469Y, M476I, Y493H, R539T, I543T, P553L, R561H, P574L, C580Y, R622I, and A675V) are validated *PfKelch13* markers of artemisinin partial resistance, whereas nine others (P441L, G449A, C469F, A481V, R515K, P527H, N537I/D, G538V, and V568G) are classified as candidate or associated markers [12]. Reports of *PfKelch13* mutations associated with artemisinin partial resistance in East Africa and the Horn of Africa, indicating a slowing of parasite clearance rates, raise concerns about compromised ACT efficacy in these regions [13–15].

In parallel, the continued emergence of *P. falciparum* strains resistant to ACT partner drugs represents an additional and substantial threat to malaria control and elimination efforts [16–23]. Resistance to partner drugs has been linked to polymorphisms in several genes, including *P. falciparum chloroquine resistance transporter (Pfcrt)*, *P. falciparum multidrug resistance 1 (Pfmdr1), P. falciparum dihydrofolate reductase (Pfdhfr), P. falciparum dihydropteroate synthase (Pfdhps)*.

The *Pfmdr1* gene encodes the *P. falciparum* multidrug resistance protein 1, a P-glycoprotein homologue belonging to the ATP-binding cassette (ABC) transporter superfamily [24–26]. This transporter is located on the membrane of the digestive vacuole, where it mediates the transport of substrates from the parasite cytoplasm into the vacuole [27, 28]. Altered drug susceptibility has been attributed to both *Pfmdr1* single nucleotide polymorphisms and increased gene copy number. Amino-acid substitutions at positions N86Y, Y184F, S1034C, N1042D, and D1246Y have been associated with resistance to multiple antimalarial drugs. Increased *Pfmdr1* copy number has been linked to reduced susceptibility to mefloquine, lumefantrine, halofantrine, quinine, and artemisinin [29–31]. In contrast, specific point mutations (S1034C, N1042D, and D1246Y) have been shown to increase parasite susceptibility to mefloquine, halofantrine and artemisinin independently of gene amplification [26, 32]. Notably, the N86Y polymorphism has been consistently associated with in vivo and in vitro responses to several quinoline antimalarials such as chloroquine and amodiaquine [23, 33]. Furthermore, the wild-type *Pfmdr1* N86 or the haplotype *Pfmdr1* NFD (N86-184F-D1246) have been reported in several studies to be associated with reduced susceptibility to lumefantrine [34, 35].

The *Pfcrt* gene is the primary determinant of chloroquine resistance [36]. The *PfCRT* protein is localised on the digestive vacuole membrane, where it facilitates the efflux of antimalarial drugs from this compartment [37]. Its physiological role is thought to involve the transport of host-derived peptides into the parasite cytoplasm [38]. The key chloroquine resistance mutation K76T, along with additional polymorphisms, has been shown to influence parasite susceptibility to quinoline drugs such as piperaquine and amodiaquine [19, 39–41].

Resistance to SP arises through mutations in the *Pfdhfr* and *Pfdhps* genes, which encode for enzymes critical to the parasite’s folate biosynthesis pathway. Pyrimethamine resistance is primarily associated with the S108N mutation in *Pfdhfr*, with additional mutations at N51I, C59R, and I164L conferring progressively higher levels of resistance [42, 43]. Sulfadoxine resistance is linked to mutations in *Pfdhps*, including S436A/F, A437G, K540E, A581G, and A613S/T [42]. Mutation at codon 581 is associated with higher resistance levels, whereas A437G and K540E confer lower-level resistance and exert modulatory effects when combined with other *Pfdhps* mutations [42, 44]. Since sulfadoxine has historically been administered in combination with pyrimethamine, resistance to SP reflects the accumulation of mutations across both *Pfdhfr* and *Pfdhps* genes [42, 45].

Although several systematic reviews and WHO surveillance reports have summarized the distribution of antimalarial drug resistance markers in Africa, most were limited to specific genes, countries, or time periods [46–49]. Furthermore, WHO reports primarily provide annual surveillance updates and do not quantitatively assess temporal trends in marker prevalence. Considering the emergence and spread of artemisinin partial resistance in Africa, there is a critical need for an updated and comprehensive assessment of antimalarial drug resistance across the continent. This study evaluates the spatial and temporal dynamics of key molecular markers associated with antimalarial drug resistance (*PfKelch13, Pfmdr1, Pfcrt, Pfdhfr*, and *Pfdhps*) in sub-Saharan Africa, synthesising evidence from studies conducted over 15 years (2010–2024).

## Methods

### Study protocol registration

Studies included in this systematic review and meta-analysis were identified and selected in accordance with the Preferred Reporting Items for Systematic Reviews and Meta-Analysis 2020 (PRISMA) guidelines [50]. The study protocol was prospectively developed and registered in the International Prospective Register of Systematic Reviews (PROSPERO; registration number CRD42023432718). The protocol was divided into two complementary components: one addressing the efficacy of ACT, which has been published previously [3], and the second focusing on molecular markers of antimalarial drug resistance. This report examines molecular markers of *P. falciparum* associated with malaria drug resistance.

### Search strategy

The literature search was conducted across multiple databases, including PubMed, EMBASE, African Journals Online, Scopus, and Web of Science. Eligible studies were those published from 2010 onwards, with sample collection periods ranging from January 2010 to December 2024. The search strategy employed combinations of the following keywords and terms: antimalarials, malaria drug, artemisinin, *Plasmodium*, *Plasmodium falciparum*, malaria, paludism, sub-Saharan Africa, resistance, molecular markers, *Plasmodium falciparum chloroquine resistance transporter (Pfcrt)*, *Plasmodium falciparum multidrug resistance 1 (Pfmdr1)*, *Plasmodium falciparum dihydrofolate reductase (Pfdhfr)*, *Plasmodium falciparum dihydropteroate synthase (Pfdhps)*, and *Plasmodium falciparum Kelch 13 (PfKelch13)* (Supplementary 1).

### Inclusion criteria

All published studies from January 2010 to December 2024 that analysed one or more molecular markers of malaria resistance (*Pfcrt, Pfmdr1, Pfdhps*, *Pfdhfr*, *PfKelch13*) in sub-Saharan Africa were considered. However, only studies that recruited participants from 2010 onward were included in this analysis. The outcome analysed in these studies was the prevalence of molecular markers of malaria drug resistance (*Pfcrt, Pfmdr1, PfKelch13, Pfdhps, Pfdhfr*).

### Exclusion criteria

The following studies were excluded: those with samples collected before 2010; transfection studies or other experimental investigations; cloning studies; studies using non-clinical samples; studies based on samples obtained after parasite culture; conference abstracts; grey literature; and studies in languages other than English or French.

### Data extraction

All retrieved records were imported into the Rayyan platform (https://rayyan.qcri.org) [51] for screening. Two independent reviewers conducted the study selection process. After removing duplicate entries, titles and abstracts were screened to identify studies that met the inclusion criteria, and any discrepancies were resolved through consensus. Studies considered eligible at this stage were subjected to full-text review, and disagreements were settled prior to data extraction. Data extraction was carried out using Covidence (https://app.covidence.org/reviews) [52], with one reviewer extracting the data and a second reviewer independently verifying the extracted information.

### Quality assessment

Study quality was assessed using an adapted Joanna Briggs Institute (JBI) checklist for prevalence studies, comprising nine items covering sampling, sample size, study description, measurement validity and reliability, data analysis, statistical methods, and response rate. Items were scored as 1 (yes or not applicable), 0.5 (unclear), or 0 (no), with total scores ranging from 0 to 9. Studies scoring > 7 were classified as low risk of bias, scores of 6–7 as moderate risk, and scores < 6 as high risk of bias [53]. The studies at low risk of bias were included in the meta-analysis.

### Data analysis

For each included study, the prevalence data of key mutations were extracted, including *Pfcrt* K76T; *Pfmdr1* (N86Y, Y184F, and the NFD (N86-184F-D1246) haplotype); *PfKelch13* (validated markers R561H, A675V, R622I, C580Y, P574L, C469Y, R539T, and candidate/associated markers G449A, P441L, P527H); *Pfdhfr* (N51I, C59R, S108N, I164L); and *Pfdhps* (I431V, S436A, A437G, K540E, A581G). Information on sample size and the laboratory methods used for mutation detection were also collected. For studies conducted at multiple sites within the same country and year, mutation counts were pooled across sites. Synthesised data were summarised using descriptive statistics, including counts, ranges, and percentages. Temporal trends in prevalence were examined by analysing annual estimates at both continental and country levels using forest plots. A meta-analysis was performed in R to generate pooled prevalence estimates and corresponding 95% confidence intervals. Study-specific proportions were pooled using a random-effects generalized linear mixed model (GLMM) with logit transformation. Individual study confidence intervals were calculated using the Clopper–Pearson exact method, and pooled estimates were back-transformed to the proportion scale. Between-study heterogeneity was assessed using Cochran’s Q test and the I^2^ statistic, and meta-regression analysis was conducted to explore sources of heterogeneity [54]. Spatial clustering of *PfKelch13* mutation prevalence (C469Y, R561H, R622I, and A675V) was assessed using Local Moran’s I implemented in the R package *spdep*. Sites within 5 decimal degrees (approximately 550 km) were considered neighbours, and row-standardised spatial weights were applied. Significant local spatial autocorrelation was identified at *p* < 0.05 and classified as High–High (hotspots), Low–Low (coldspots), High–Low, or Low–High clusters (spatial outliers)

## Results

### Literature search and study characteristics

**Fig. 1:**
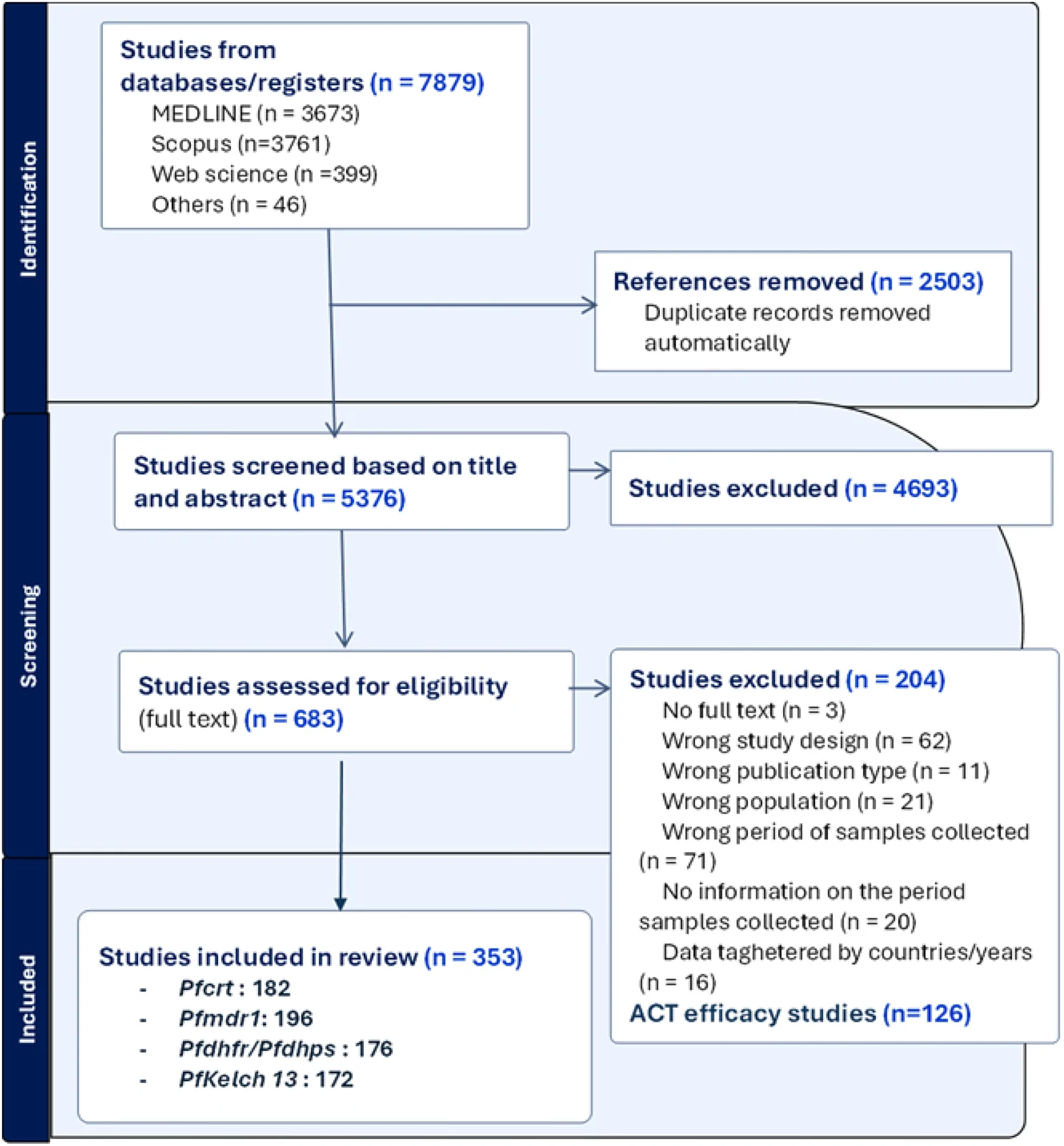
Preferred reporting items for systematic reviews and meta-analyses (PRISMA 2020) flow diagram of the study selection process. The figure illustrates the identification, screening, eligibility assessment, and inclusion of studies in the systematic review and meta-analysis of *Plasmodium falciparum* antimalarial drug resistance markers in sub-Saharan Africa from 2010 to 2024. A total of 353 studies met the predefined eligibility criteria and were included in the systematic review, with eligible data incorporated into the quantitative meta-analyses where appropriate. n denotes the number of records or studies at each stage of the selection process. PRISMA, Preferred reporting items for systematic reviews and meta-analyses

The systematic search of electronic databases yielded 7,879 records, of which 683 articles were included for full-text assessment. Overall, 353 studies met the inclusion criteria for the molecular marker review, including investigations of *Pfcrt* (*n* = 182), *Pfmdr1* (*n* = 196), *Pfdhfr/Pfdhps* (*n* = 176), and *PfKelch13* (*n* = 172) (Fig. 1, PRISMA flow diagram, Supplementary 2). Risk-of-bias evaluation showed that almost all included studies were at low risk of bias; a single study was classified as having a moderate risk of bias (sample size not available) and was therefore excluded from the meta-analysis (Supplementary 2). Among the 352 studies included, publication output increased steadily over time, peaking between 2020 and 2021 (12.2% and 11.9%, respectively), with sustained high numbers through 2023–2024 (21.6% combined) (Supplementary 3_Table 1).

The geographic distribution of molecular surveillance studies conducted between 2010 and 2024 showed that Eastern Africa contributed with the most significant number of studies, driven by Uganda (*Pfcrt,n* = 35; *Pfmdr1*, *n* = 38; *PfKelch13*, *n* = 27; *Pfdhfr/Pfdhps*, *n* = 17) and Kenya (*Pfcrt*, *n* = 22; *Pfmdr1*, *n* = 20; *PfKelch13*, *n* = 21; *Pfdhfr/Pfdhps*, *n* = 16). Western Africa was also strongly represented, particularly Ghana (*Pfcrt,n* = 17; *Pfmdr1*, *n* = 19; *PfKelch13*, *n* = 19; *Pfdhfr/Pfdhps*, *n* = 21) and Nigeria (*Pfcrt*, *n* = 11; *Pfmdr1*, *n* = 11; *PfKelch13*, *n* = 9; *Pfdhfr/Pfdhps*, *n* = 18). Central Africa showed moderate but fragmented contributions, mainly from Cameroon (*Pfcrt,n* = 10; *Pfmdr1*, *n* = 8; *PfKelch13*, *n* = 9; *Pfdhfr/Pfdhps*, *n* = 15) and the Democratic Republic of Congo (*Pfcrt*, *n* = 11; *Pfmdr1*, *n* = 9; *PfKelch13*, *n* = 14; *Pfdhfr/Pfdhps*, *n* = 13). In contrast, Southern and Northern Africa were sparsely represented across all markers, and study output declined markedly after 2019 (Supplementary 3 _Tables 2 3, 4, and 5).

### Methods used for analysing molecular markers

**Fig. 2:**
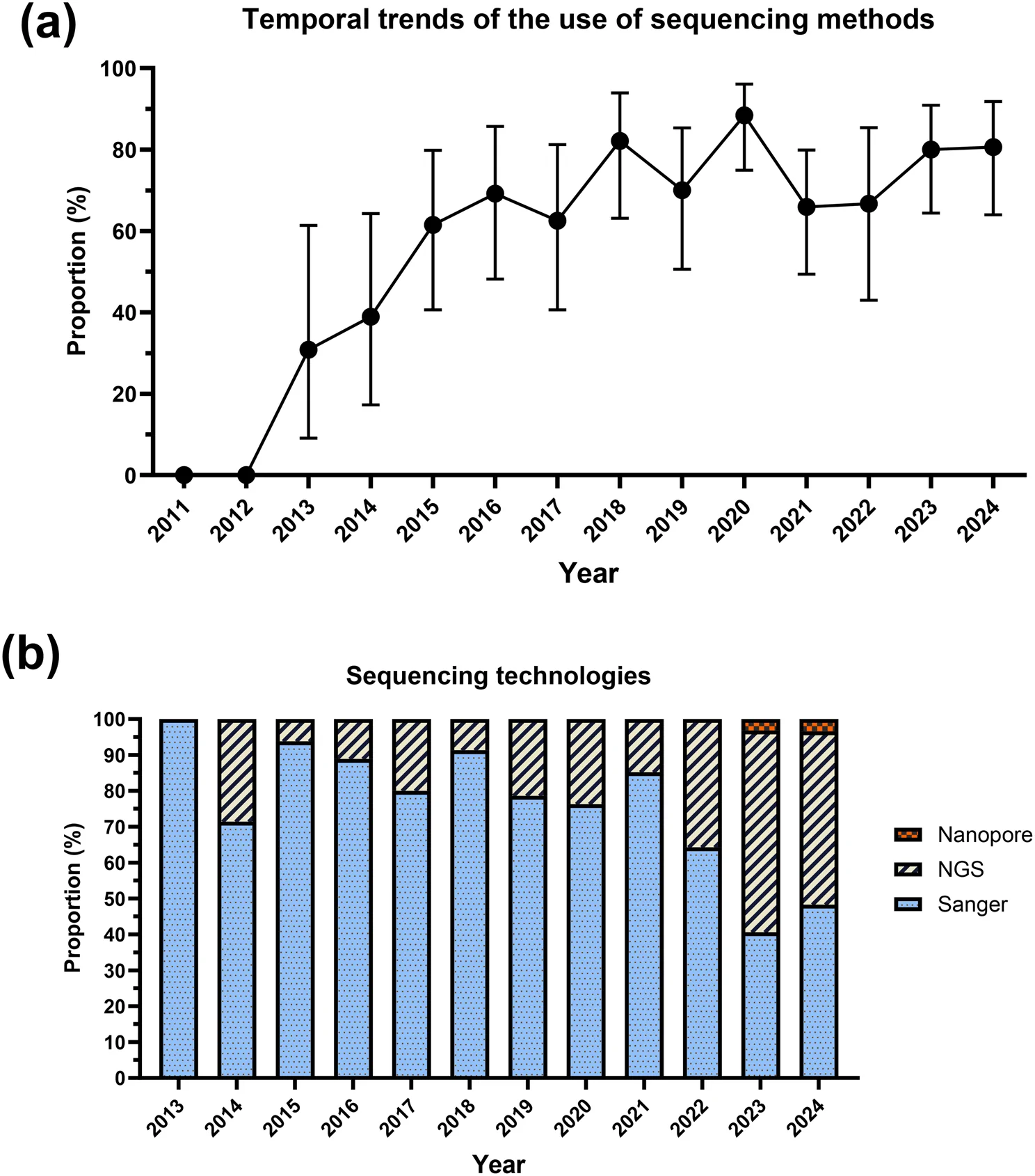
Trends in the use of sequencing methods and sequencing technologies among included malaria molecular surveillance studies in sub-Saharan Africa. (**a**) Annual proportion of included studies published between 2011 and 2024 that used sequencing methods for molecular analysis. Each point represents the proportion of studies published in a given year that used sequencing, and the error bars indicate the corresponding 95% confidence intervals (95% CI). (**b**) Annual distribution of sequencing technologies among studies using sequencing, expressed as the percentage of studies employing Sanger sequencing, next-generation sequencing (NGS), or nanopore sequencing. Each stacked bar represents the total number of publications in that year, and the coloured segments indicate the proportion of studies using each sequencing technology

The use of sequencing methods increased substantially over time. Indeed, sequencing was not reported between 2010 and 2012, then rose from 33.3% (4/12) in 2013 to 57.1% (16/28) in 2015 and 72.0% (18/25) in 2016, becoming the dominant approach in published studies from 2018 onward, peaking at 88.4% (38/43) in 2020. In sequencing approaches, Sanger sequencing predominated until 2018, but NGS contributions steadily increased from 2019, surpassing Sanger sequencing in 2023 (56.2%; 18/32) (Fig. 2; Supplementary 3 _Tables 7 and 8).

### Artemisinin partial resistance: *PfKelch13*

#### Spatial distribution of validated *Pfkelch13* mutations in Africa

**Fig. 3:**
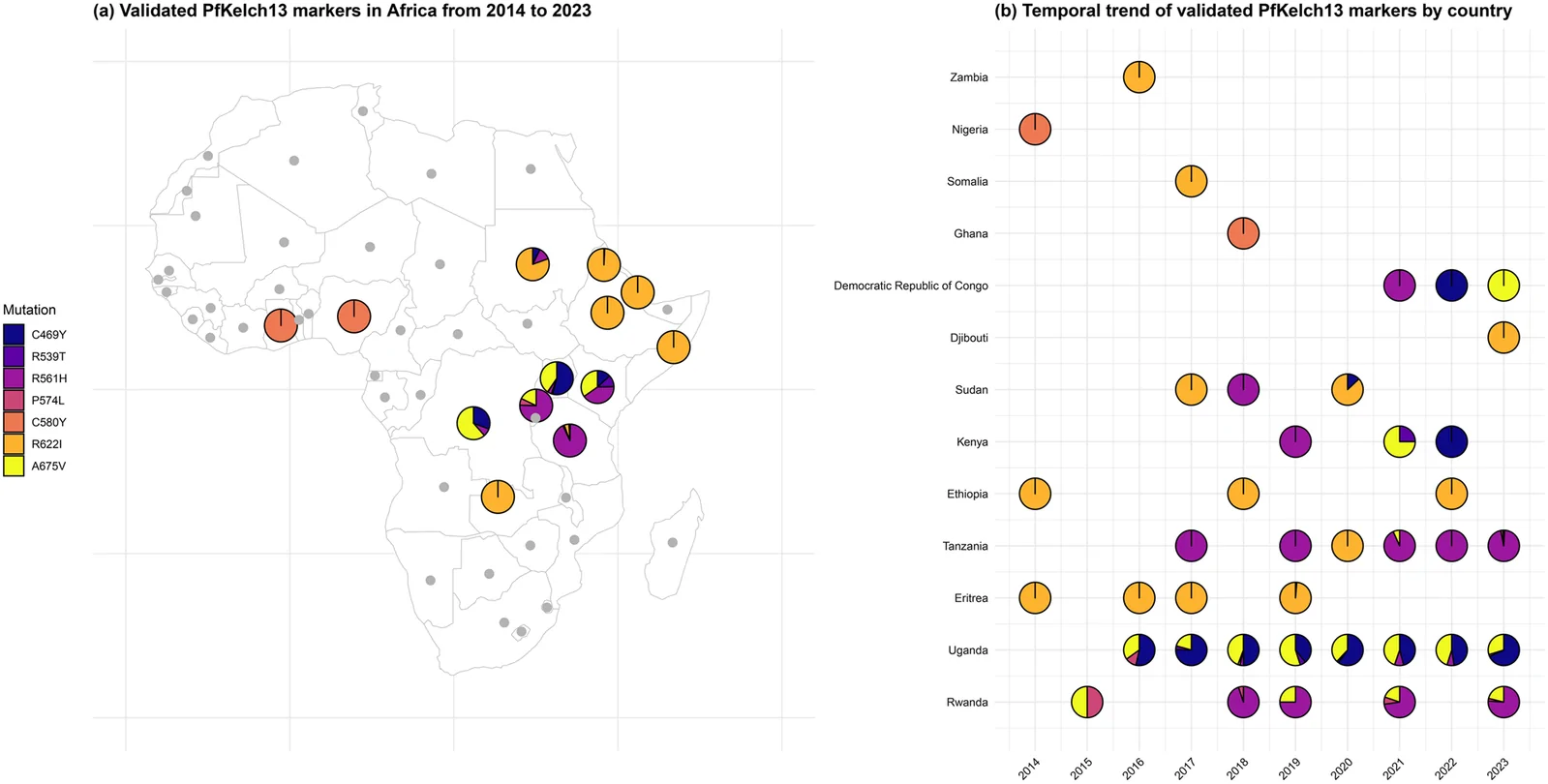
Spatial and temporal distribution of validated *PfKelch13* mutations reported in sub-Saharan Africa from 2014 to 2023. (**a**) Geographic distribution of validated *PfKelch13* mutations identified across sub-Saharan Africa. Each pie chart represents a country in which validated *PfKelch13* mutations were reported, and each coloured slice corresponds to the relative proportion of a specific validated mutation (C469Y, R539T, R561H, P574L, C580Y, R622I, or A675V) among all validated mutations reported for that country during the study period. Countries without validated mutations are shown as grey dots. (**b**) Temporal distribution of validated *PfKelch13* mutations by country from 2014 to 2023. Each pie chart represents the relative proportion of validated *PfKelch13* mutations reported in a given country and year, with colours indicating the mutation type as shown in the legend

From 2014 to 2023, the validated *PfKelch13* mutations R561H and A675V were the most geographically widespread, with repeated detection in Uganda, Rwanda, Tanzania, and the Democratic Republic of Congo. In several East African settings, mutation prevalences reached approximately 10–20% in recent years, particularly for R561H (Rwanda 2021: 24% (20/85); Rwanda 2023: 18% (515/2845); Tanzania 2023: 12% (186/1534)). R622I showed a distinct pattern, predominating in the Horn of Africa (Ethiopia (2022: 11% (35/328), Eritrea (2019: 21% (69/329)), Sudan (2020: 2% (7/352)), with prevalences commonly in the 2–21% range, and intermittent detections in East Africa (Tanzania 2020 (1/95); Uganda 2018 (1/1093)). In contrast, C580Y was rare, confined to isolated reports in West Africa, notably Ghana (2018: 5/530) and Nigeria (2014: 1/172), where prevalence generally remained below 1% (Fig. 3a; Supplementary 4).

#### Temporal trends of validated *PfKelch13* mutations by country

Temporal analysis revealed a progressive increase in both the diversity and prevalence of validated *PfKelch13* mutations in East Africa. Uganda showed the most sustained and diverse pattern, with A675V, C469Y, R561H, R539T, and R622I detected repeatedly since 2015. In Uganda, the prevalence of A675V increased steadily after 2018, rising from 2% (25/1168) in 2018 to 9% (98/1077) in 2022. Similarly, C469Y increased from 3% (29/999) in 2017 to 10% (98/1077) in 2022. However, other mutations remained below 1% in the country over time. In Rwanda, the prevalence of R561H showed a marked temporal increase from 15% (38/254) in 2018 to approximately 24% (20/85) by 2021, representing the highest levels observed across countries, and it persisted at 18% (515/2845) in 2023. A675V was also detected repeatedly in Rwanda, though generally at lower prevalence, increasing from 2% (1/66) in 2019 to 6% (6/94) and 5% (144/2850) in 2021 and 2023, respectively. Tanzania and Kenya showed increasing mutation diversity after 2018, mainly driven by A675V and R561H. For instance, in Tanzania, the prevalence of R561H increased from 0.3% (2/774) in 2017 to 12% (186/1534) in 2023. In contrast, the prevalences of A675V and R561H were approximately 2% (A675V: 3/154; R561H: 15/659) in Kenya. In Central Africa, the Democratic Republic of Congo showed a later emergence of validated mutations, with A675V, C469Y and R561H detected mainly after 2020. However, the prevalence remained low, at less than 1% (A675V 2023: 5/635; C469Y 2022: 1/256, and R561H in 2021: 1/936). By contrast, West African countries showed limited temporal activity, with sporadic C580Y detections remaining consistently low (<1%) in Ghana (2018: 0.9% (5/530)) and Nigeria (2014: 0.6% (1/172)), and without evidence of sustained upward trends (Fig. 3b, Supplementary 4).

#### Mutation-specific dynamics

**Fig. 4:**
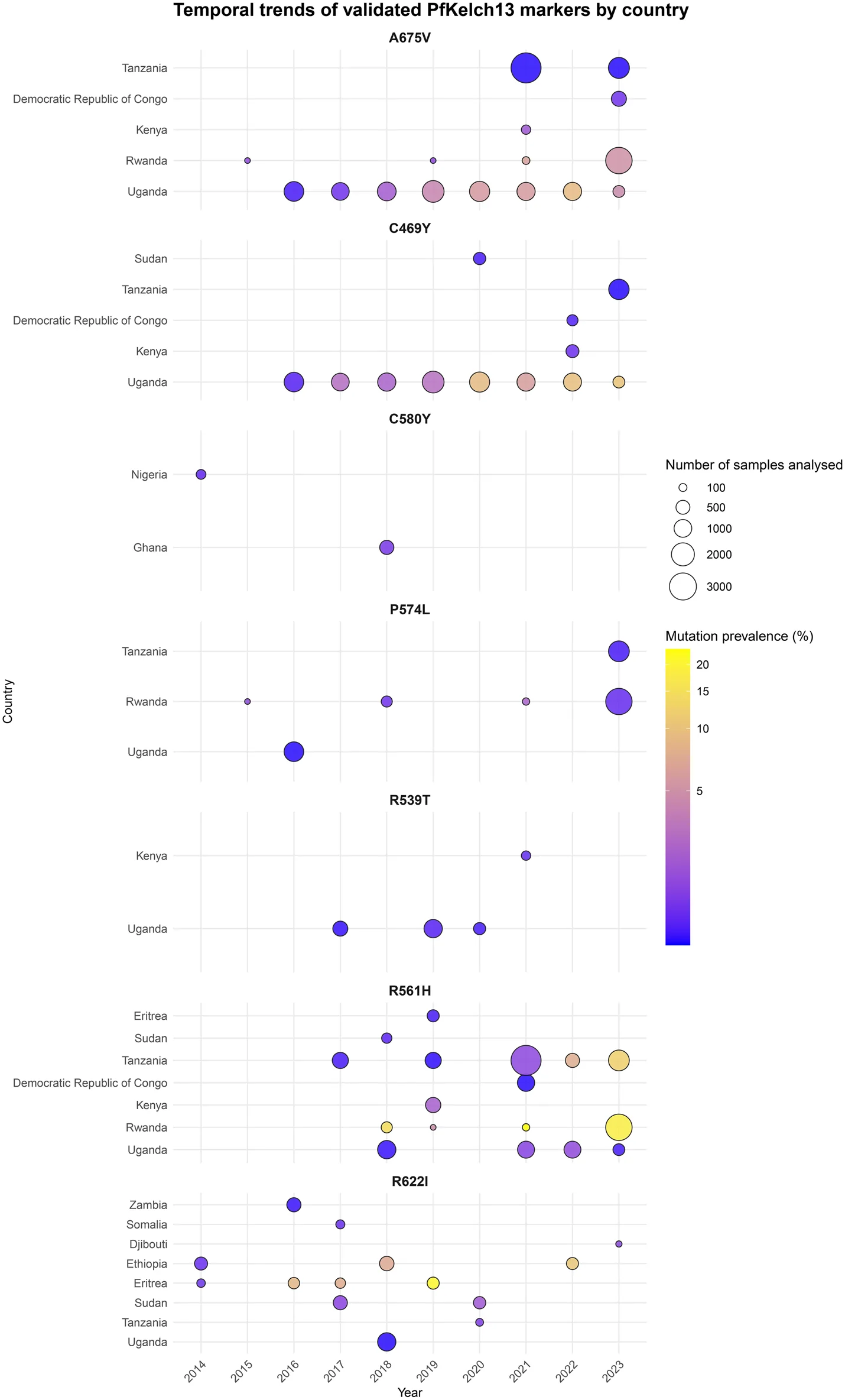
Country-level temporal trends in the prevalence of validated *PfKelch13* mutations in sub-Saharan Africa from 2014 to 2023. Each panel represents one validated *PfKelch13* mutation (A675V, C469Y, C580Y, P574L, R539T, R561H, and R622I). Each circle corresponds to a country-year observation. The colour of the circles indicates the reported mutation prevalence (%) according to the colour scale, whereas the size of the circles is proportional to the number of samples analysed. Only country-year combinations in which the corresponding mutation was detected are displayed

**Fig. 5:**
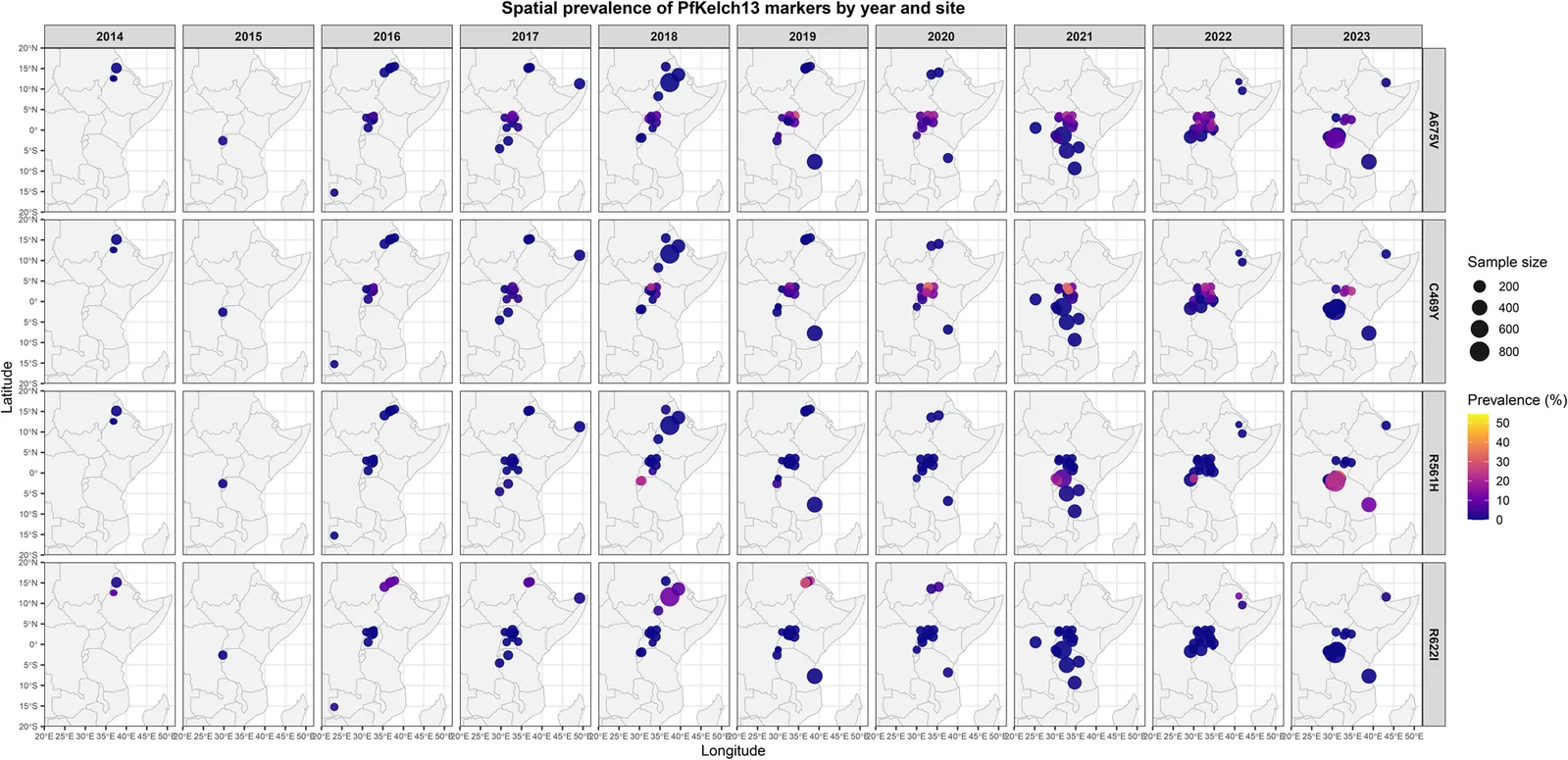
Spatio-temporal distribution of the prevalence of validated *PfKelch13* mutations (A675V, C469Y, R561H, and R622I) across study sites in sub-Saharan Africa from 2014 to 2023. Each column represents a study year (2014–2023), and each row corresponds to one validated *PfKelch13* mutation. Each circle represents a study site with reported prevalence greater than 0%. The colour of the circles indicates the reported mutation prevalence (%) according to the colour scale, while the size of the circles is proportional to the number of samples analysed at each site. Only study sites with available prevalence estimates greater than 0% are displayed

A675V emerged in 2015–2016 in Rwanda and Uganda, and expanded geographically to Tanzania and Kenya, becoming one of the most frequently reported validated mutations by 2021–2023, with prevalences commonly reported at 6–9% in East African countries. C469Y was largely confined to Uganda, persisting at 2–10% over multiple years. R561H showed the most concerning temporal signal, with a clear increase of prevalence in Rwanda and Tanzania, reaching up to 20%. R539T remained uncommon and geographically restricted, with most reports from Uganda and sporadic reports from Kenya, at a prevalence generally below 5%. R622I demonstrated moderate prevalence but broad geographic spread, particularly in the Horn of Africa, where prevalences ranged from 5% to 15% (Figs. 4 and 5).

**Fig. 6:**
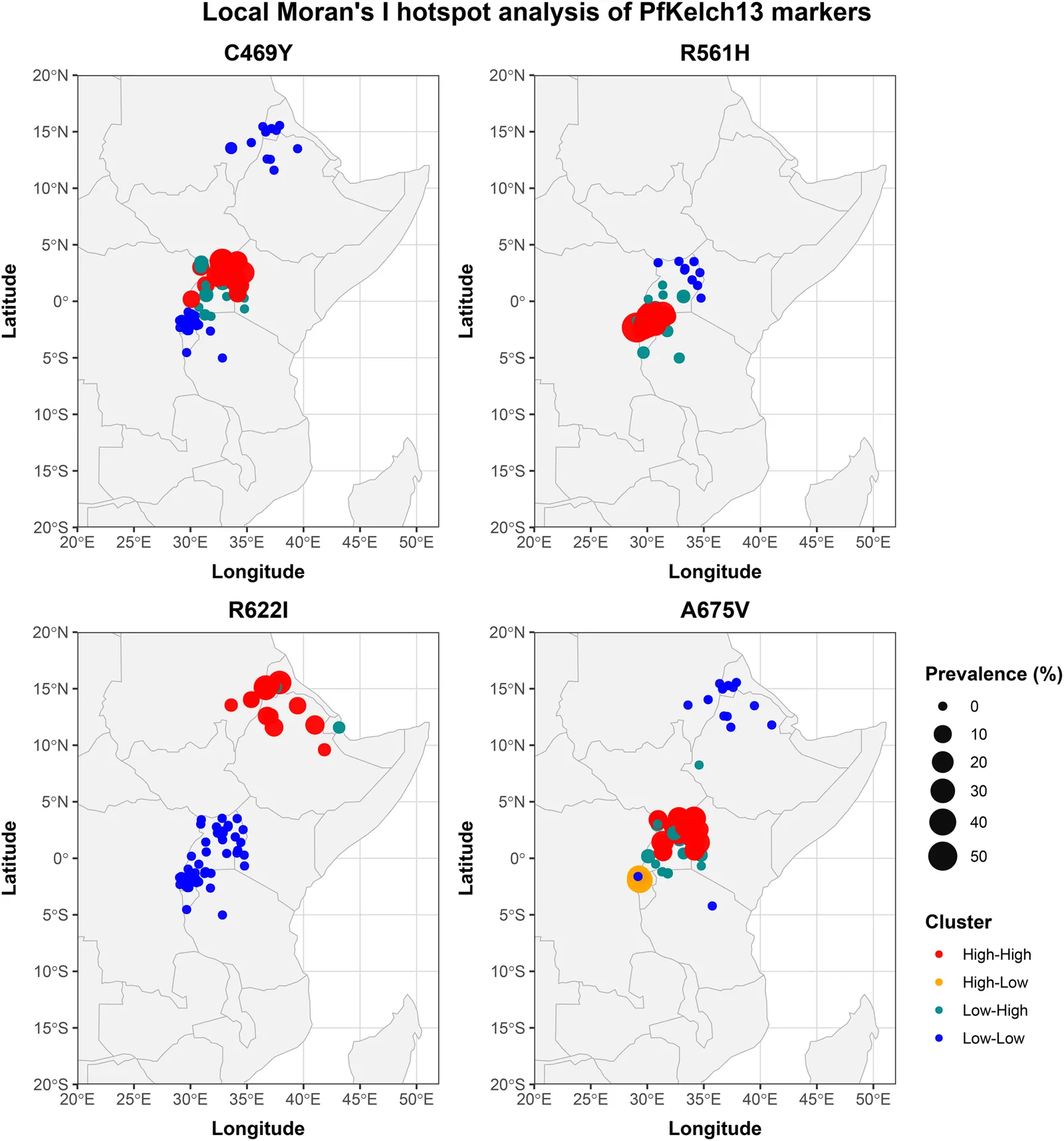
Local Moran’s I hotspot analysis of the prevalence of validated *PfKelch13* mutations (C469Y, R561H, R622I, and A675V) across study sites in sub-Saharan Africa. Each panel represents one validated *PfKelch13* mutation. Circles correspond to individual study sites, with circle size proportional to the reported mutation prevalence (%). Spatial autocorrelation was assessed using local Moran’s I, and statistically significant clusters (*p* < 0.05) were classified as high–high (hotspots), low–low (coldspots), high–low (high-prevalence outliers surrounded by low-prevalence neighbours), and low–high (low-prevalence outliers surrounded by high-prevalence neighbours). Colours indicate the cluster type as shown in the legend. Only study sites belonging to statistically significant clusters are displayed. The analysis identified high–high clusters of C469Y and A675V predominantly in Uganda, R561H in the Rwanda–Uganda region, and R622I in Ethiopia and Eritrea

Although the prevalence of some mutations increased over time, their distribution remained geographically clustered. Local Moran’s I analysis identified significant spatial clustering for *PfKelch13* mutations (Fig. 6). C469Y and A675V showed hotspots primarily in northern Uganda, whereas R561H clustered in the Rwanda–Uganda region. R622I exhibited the strongest hotspot pattern, concentrated in Ethiopia and Eritrea.

#### Associated/Candidate *PfKelch13* markers

Associated *PfKelch13* mutations (G449A, P441L, and P527H) were detected at low prevalence (<5%) and in limited geographic regions. G449A was reported sporadically in Ghana (2018: 0.4%; 2/530), Nigeria (2018: 0.5%; 2/414), and Rwanda (2023: 3.4%; 26/2854), with a prevalence generally below 1%. P441L was mainly observed in Uganda, Tanzania, and Rwanda, with a gradual increase over time, reaching a prevalence of 3.4% in Uganda (36/1058) in 2022 and 3.8% in Tanzania (56/1462) in 2023. P527H was extremely rare, detected only once in Cameroon (2014: 1/378), indicating an isolated occurrence without evidence of spread (Supplementary 4; Supplementary 5 _ Fig. 1s).

### Resistance to partner drug: *Pfcrt* and *Pfmdr1*

**Fig. 7:**
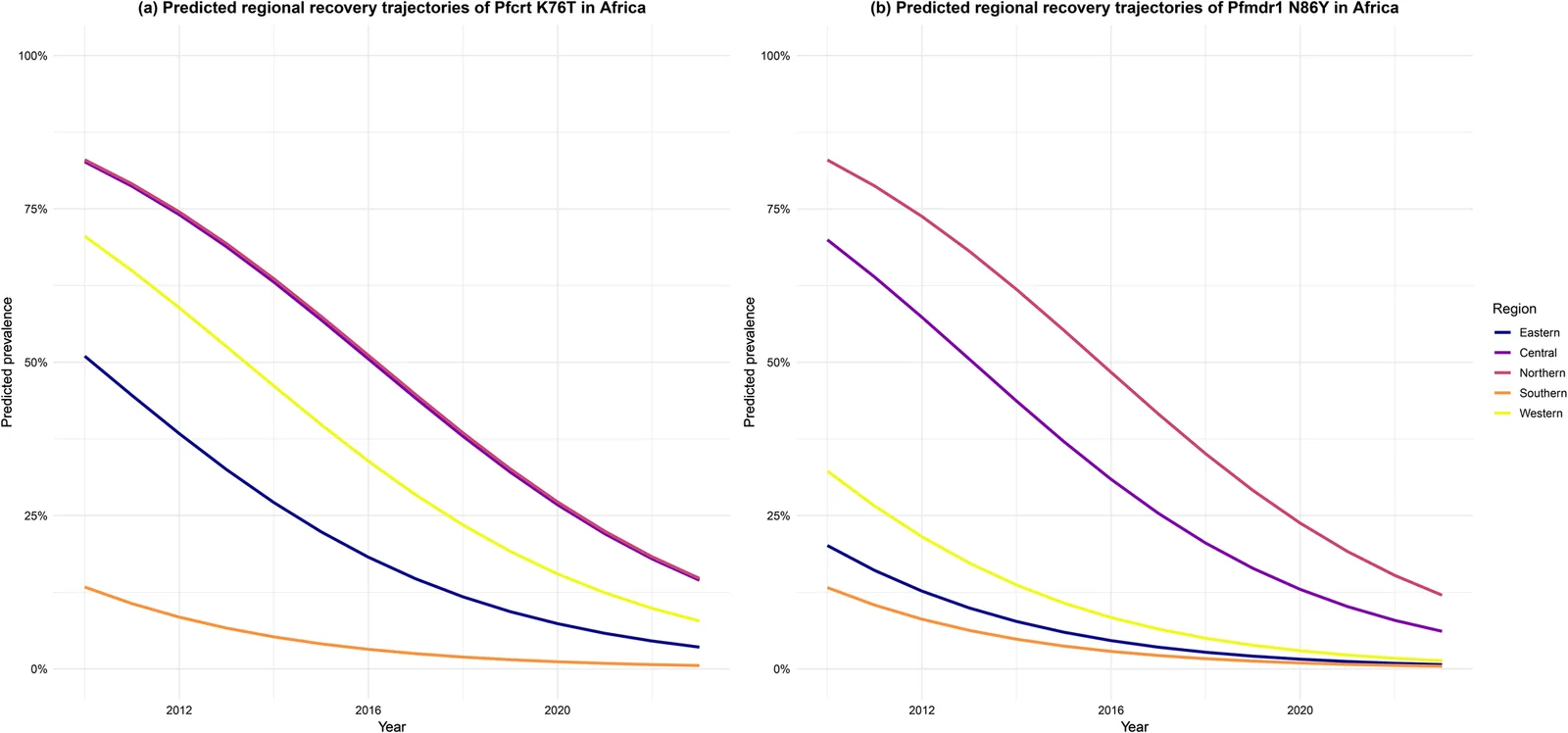
Predicted regional trajectories of the prevalence of *Pfcrt K76T* (**a**) and *Pfmdr1 N86Y* (**b**) across sub-Saharan Africa from 2010 to 2024. Predicted mutation prevalences were estimated from multivariable random-effects meta-regression models adjusted for year of study, sample size, and study density. The coloured curves represent the model-predicted prevalence for each African region (Eastern, Central, Northern, Southern, and Western Africa). Predicted values are presented on the prevalence scale following back-transformation from the logit model. Overall, declining trends were observed across all regions throughout the study period, although regional heterogeneity persisted, with higher predicted prevalences in Central and Northern Africa for both markers than in Eastern and Southern Africa

The random-effects meta-analysis in sub-Saharan Africa showed a marked reduction in pooled prevalence of *Pfcrt* K76T from 76% (95% CI: 49–91%; *n* = 5,614) in 2010 to 9% (*n* = 4,273) in 2023. Likewise, *Pfmdr1* N86Y prevalence declined from 34% (95% CI: 26–43%; *n* = 3,609) in 2010 to 0% (95% CI: 0–11%; *n* = 4,834) in 2023 (Supplementary 5_ Figs. 2 and 3; Supplementary 6). Meta-regression analysis across Africa confirmed significant temporal declines for both markers while highlighting persistent geographic heterogeneity. For *Pfcrt* K76T (170 prevalence estimates), prevalence decreased by an average of 23% per year (OR 0.77, 95% CI 0.71–0.84; *p* < 0.0001; I^2^ = 99.5%, R^2^ = 29.1%), independent of sample size and sampling intensity, African region. Regional analysis across Africa showed that although prevalence declines in all regions over time, it remained consistently higher in Central Africa (OR 4.57, 95% CI 1.99–10.48) and Western Africa (OR 2.29, 95% CI 1.14–4.62) compared with Eastern Africa. Similarly, *Pfmdr1* N86Y (166 estimates) declined by an average of 24% per year (OR 0.76, 95% CI 0.72–0.80; *p* < 0.0001; I^2^ = 97.7%, R^2^ = 60.6%), independent of study size and density and African region. The analysis by African region showed that although prevalence declined across all regions over time, it remained substantially higher in Central Africa (OR 9.25, 95% CI 5.58–15.34), Northern Africa (OR 19.36, 95% CI 6.06–61.84), and Western Africa (OR 1.89, 95% CI 1.19–3.01) compared to Eastern Africa. Given the substantial residual heterogeneity (*Pfcrt* K76T model: I^2^ = 99.5%, R^2^ = 29.1%; *Pfmdr1* N86Y model: I^2^ = 97.7%, R^2^ = 60.6%), these estimates should be interpreted as reflecting the overall direction of the temporal trend rather than the precise annual change in prevalence. (Table 1; Fig. 7).

Overall, sequencing-based approaches were employed in 69.0% (243/352) of studies, while 31.0% (109/352) relied solely on non-sequencing methods. Among sequencing studies, Sanger sequencing was the most frequently technique used (72.4%; 176/243), followed by next-generation sequencing (NGS) (21.8%; 64/243), whereas nanopore sequencing was rarely applied (0.8%; 2/243). Non-sequencing studies were predominantly based on Restriction Fragment Length Polymorphism (RFLP), accounting for 54.9% (78/142) (Supplementary 3 _Table 6).

**Table 1:** Odds ratio (OR) and *p*-values from multivariable meta-regression analysis assessing temporal trends in the prevalences of *Pfcrt* K76T and *Pfmdr1* N86Y mutations from 2010 to 2023 across sub-Saharan Africa. The model was adjusted for year, sample size, African region and the number of studies published per year within each African region

Moderator	*Pfcrt* K76T OR (95% CI)	*p*-value	*Pfmdr1* N86Y OR (95% CI)	*p*-value
Year (per year)	**0.77 (0.71–0.84)**	**<0.0001**	**0.76 (0.72–0.80)**	**<0.0001**
Sample size	1.00 (1.00–1.00)	0.239	1.00 (1.00–1.00)	0.197
Africa region				
Eastern	Ref		Ref	
Central	**4.57 (1.99–10.48)**	**<0.001**	**9.25 (5.58–15.34)**	**<0.0001**
Northern	4.71 (0.64–34.50)	0.128	**19.36 (6.06–61.84)**	**<0.0001**
Southern	0.15 (0.01–1.57)	0.113	0.61 (0.14–2.56)	0.497
Western	**2.29 (1.14–4.62)**	**0.019**	**1.89 (1.19–3.01)**	**0.007**
Studies per year–region	1.02 (0.86–1.20)	0.806	1.02 (0.95–1.11)	0.556

**Bold** indicates statistically significant associations (*p* < 0.05)

**Model fit:**

*Pfcrt* K76T—τ^2^ = 3.27; I^2^ = 99.5%; R^2^ = 29.1%; AIC = 735.8

*Pfmdr1* N86Y—τ^2^ = 1.10; I^2^ = 97.7%; R^2^ = 60.6%; AIC = 552.7

Odds ratios are relative to the reference region (Eastern Africa)

Across sub-Saharan Africa between 2010 and 2023, the pooled prevalence from the random-effects forest plot for *Pfmdr1* Y184F and NFD haplotype exhibited distinct yet overlapping temporal patterns, characterised by fluctuating prevalence rather than consistent directional trends. *Pfmdr1* Y184F increased from 57 to 58% in 2010–2011 to 67–78% in 2012–2014, followed by a transient decline in 2017–2021 (57–60%), and a slight increase in 2023 (72%). The pooled prevalence of *Pfmdr1* NFD haplotype showed global stability over time, ranging from 47% to 56%, except in 2011, when it was estimated at 19% (*N* = 1553). It was 47% in 2010 (*N* = 1046), fluctuating at intermediate levels during the period 2012–2015 (41–44%), increasing transiently in 2016 (54%; *N* = 1778), and stabilising at moderate levels between 2020 and 2023 (47–56%). For both markers, between-country heterogeneity remained consistently high (*I*^*2*^ typically > 85%), with higher prevalence in East Africa and more variable or lower levels in parts of West and Central Africa (Supplementary 5 _ Figs. 4 and 5; Supplementary 6).

### SP resistance: *Pfdhfr* and *Pfdhps*

**Fig. 8:**
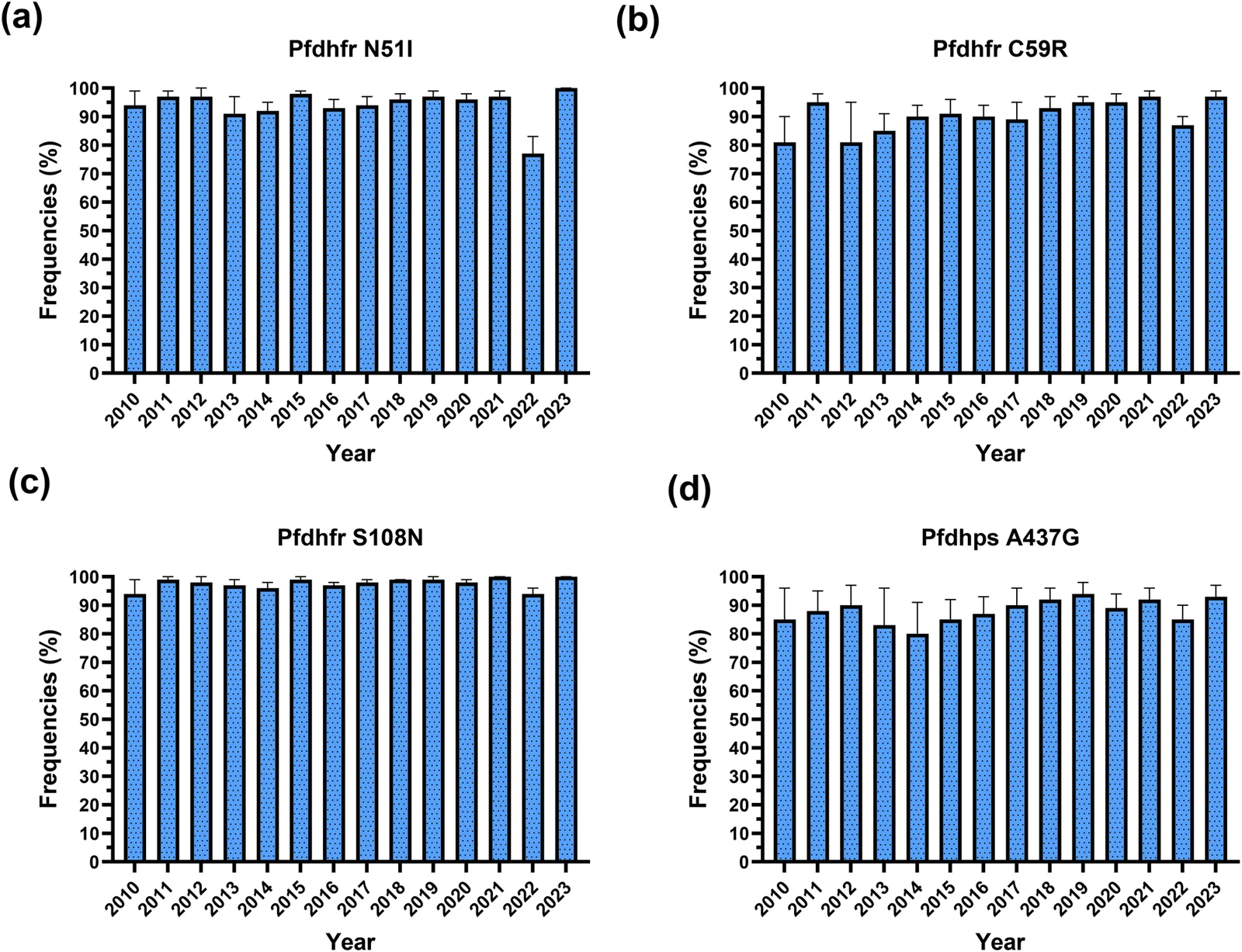
Annual pooled prevalence of sulfadoxine–pyrimethamine resistance-associated *Pfdhfr* and *Pfdhps* mutations across sub-Saharan Africa from 2010 to 2023. Panels show the annual pooled prevalence estimates for (**a**) *Pfdhfr* N51I, (**b**) *Pfdhfr* C59R, (**c**) *Pfdhfr* S108N, and (**d**) *Pfdhps* A437G. Bars represent the pooled prevalence estimated using the random-effects meta-analysis for each calendar year, while the error bars indicate the corresponding 95% confidence intervals (95% CI). The figure illustrates the persistently high prevalence of these sulfadoxine–pyrimethamine resistance-associated mutations throughout the study period across sub-Saharan Africa

**Fig. 9:**
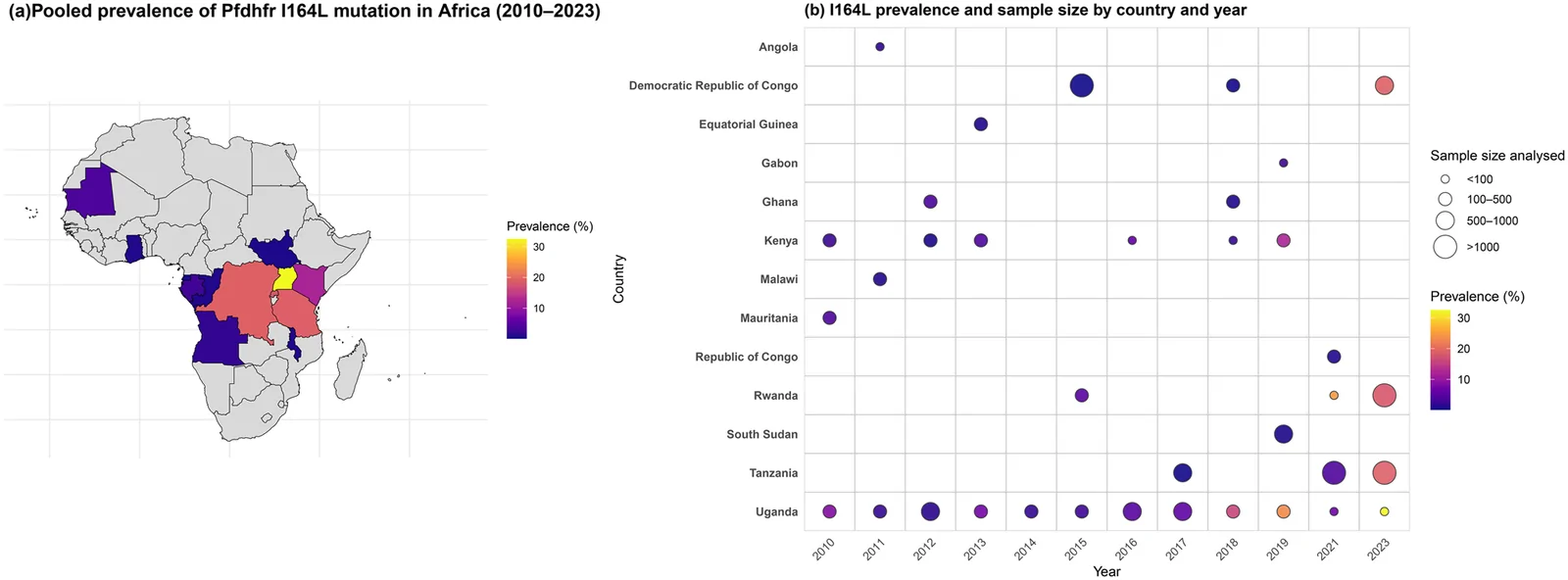
Spatial distribution and country-level temporal trends in the pooled prevalence of the *Pfdhfr* I164L mutation across sub-Saharan Africa from 2010 to 2023. (**a**) Geographic distribution of the pooled prevalence of the *Pfdhfr* I164L mutation by country. Countries with available data are coloured according to the pooled prevalence (%) shown on the accompanying colour scale, whereas countries without eligible data are shown in grey. (**b**) Country-level temporal distribution of the *Pfdhfr* I164L mutation from 2010 to 2023. Each circle represents a country-year observation. Circle size is proportional to the number of samples analysed, and circle colour indicates the reported mutation prevalence (%) according to the colour scale. This figure illustrates the spatial heterogeneity and temporal occurrence of the *Pfdhfr* I164L mutation across sub-Saharan Africa during the study period

Temporal trends from the forest plot of random-effects meta-analysis showed that the prevalence of *Pfdhfr* N51I, C59R, and S108N, together with *Pfdhps* A437G, remained consistently high across sub-Saharan Africa between 2010 and 2023, with extensive overlap of 95% confidence intervals across years, indicating stable temporal patterns. *Pfdhfr* N51I was near fixation throughout the study period, with pooled prevalence ranging from 77% to 100%, while C59R also showed persistently high prevalence (81–97%). S108N showed the greatest temporal stability, with prevalence consistently ranging from 94% to 100%. Likewise, *Pfdhps* A437G remained highly prevalent over time, with pooled estimates ranging from 80% to 94% (Fig. 8; Supplementary 4 _ Figs. 6, 7, 8 and 9; Supplementary 7).

In contrast, *Pfdhfr* I164L displayed pronounced spatial heterogeneity and limited temporal expansion. During the early period (2010–2014), I164L was rare, with prevalence generally below 10% in most countries, including Ghana (2012: 4%; 8/200), Kenya (2013: 4.3%; 13/304), and Uganda (2013: 7.2%; 25/347). From 2015 onwards, sporadic increases were observed but were not sustained and remained confined to a small number of settings. In the most recent years (2019–2023), I164L persisted at low to moderate prevalence, with localized peaks of approximately 20–30% in Rwanda (2021: 24.7%; 19/77), Uganda (2023: 32.9%; 24/73), the Democratic Republic of Congo (2023: 19.1%; 116/606), and Tanzania (2023: 18.9%; 278/1470). Spatially, I164L showed a focal distribution, with higher prevalence mainly in East Africa, whereas it remained rare or absent in West and Central Africa. The pooled prevalence of *Pfdhfr* I164L in the Eastern Africa region increased from 1% (95% CI: 0–11%; I^2^ = 27.8%) in 2010 to 19% (17–20%; I^2^ = 79.8%) in 2023, whereas it was 0% over time in the other African region (Fig. 9; Supplementary 7; Supplementary 8). 

**Fig. 10:**
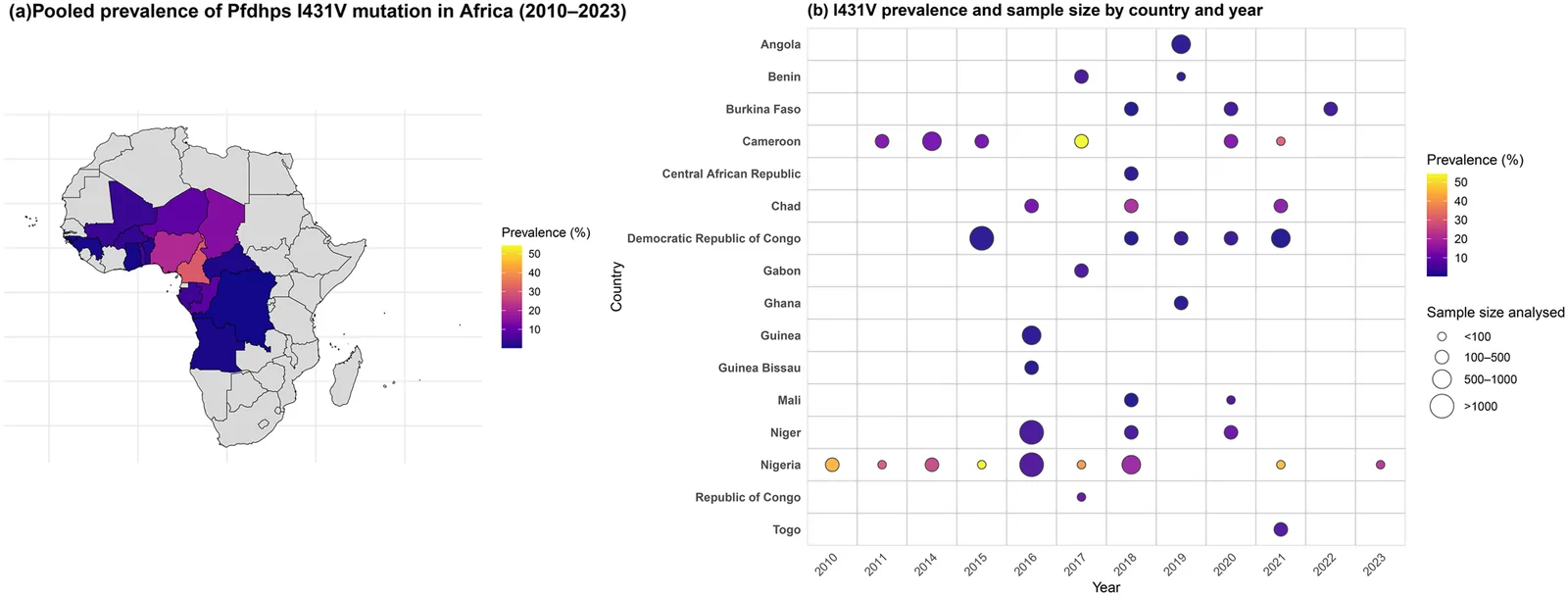
Spatial distribution and country-level temporal trends in the pooled prevalence of the *Pfdhps* I431V mutation across sub-Saharan Africa from 2010 to 2023. (**a**) Geographic distribution of the pooled prevalence of the *Pfdhps* I431V mutation by country. Countries with available data are coloured according to the pooled prevalence (%) shown on the accompanying colour scale, whereas countries without eligible data are shown in grey. (**b**) Country-level temporal distribution of the *Pfdhps* I431V mutation from 2010 to 2023. Each circle represents a country-year observation. Circle size is proportional to the number of samples analysed, and circle colour indicates the reported mutation prevalence (%) according to the colour scale. This figure highlights the spatial heterogeneity and temporal emergence of the *Pfdhps* I431V mutation across sub-Saharan Africa during the study period

The *Pfdhps* I431V mutation was detected intermittently, with prevalence generally remaining low to moderate (5–30%) in most settings but reaching high levels (>40–50%) in a limited number of countries. Markedly elevated prevalence was observed in Nigeria (2015: 54%; 51/95) and Cameroon (2017: 54%; 76/140). Since 2010, I431V has been consistently reported in Nigeria and Cameroon, with prevalence ranging from 5 to 54% and 11–54%, respectively. Elsewhere, detections were sporadic and typically at a low prevalence level, including in Niger (2020: 9%; 13/139), Burkina Faso (2020: 3%; 7/216), Mali (2020: 4%; 4/97), Togo (2021: 5%; 19/357), as well as in Central Africa, notably the Democratic Republic of Congo (2020: 2%; 8/337) and Chad (2021: 14%; 31/214). Overall, the pooled prevalence of I431V was highest in West (18%; 95% CI: 3–59%; I^2^ = 98.3% in 2021) and Central (7%; 95% CI: 1–40%; I^2^ = 96% in 2021) Africa, while remaining rare or absent in most East (0%) and Southern (0%) African settings. (Fig. 10; Supplementary 7; Supplementary 8).

**Fig. 11:**
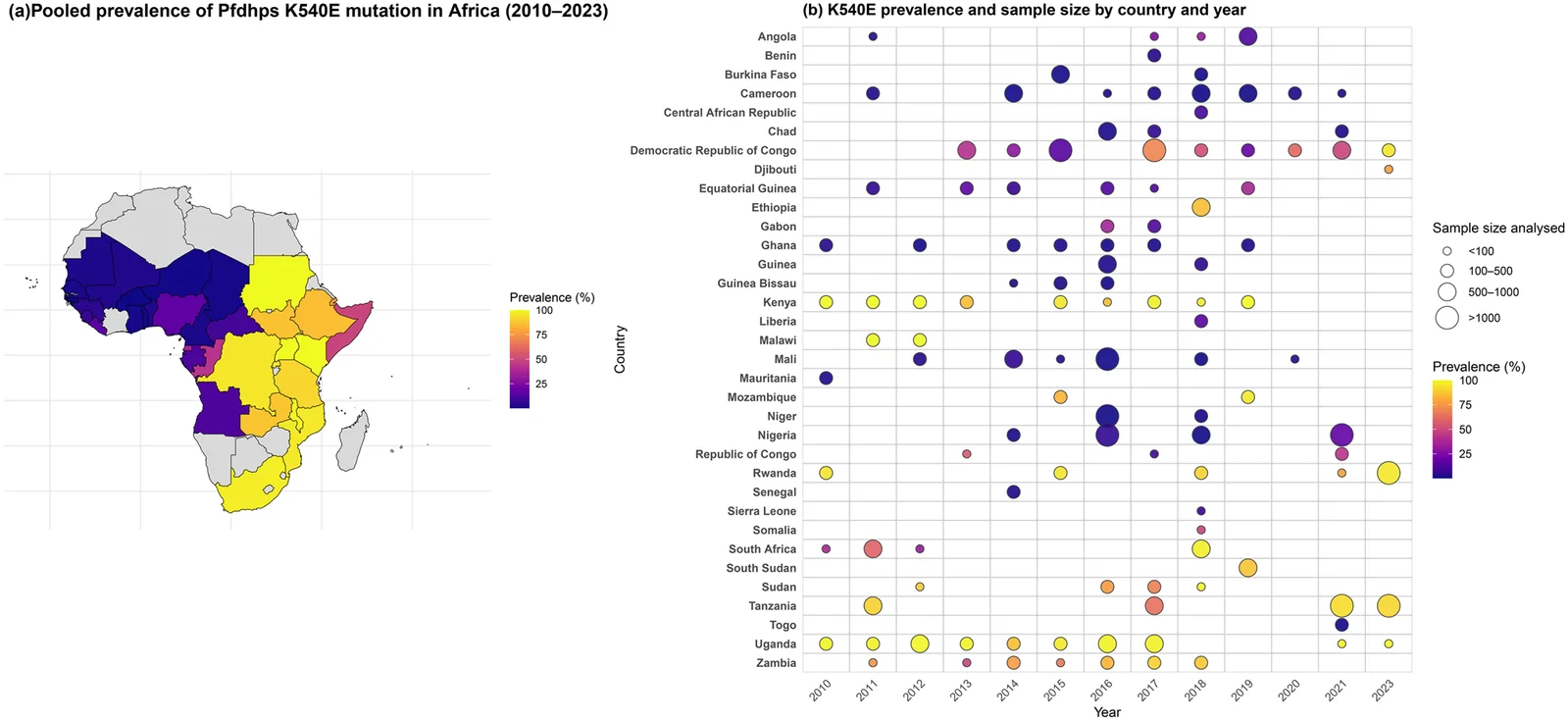
Spatial distribution and country-level temporal trends in the pooled prevalence of the *Pfdhps* K540E mutation across sub-Saharan Africa from 2010 to 2023. (**a**) Geographic distribution of the pooled prevalence of the *Pfdhps* K540E mutation by country. Countries with available data are coloured according to the pooled prevalence (%) shown on the accompanying colour scale, whereas countries without eligible data are shown in grey. (**b**) Country-level temporal distribution of the *Pfdhps* K540E mutation from 2010 to 2023. Each circle represents a country-year observation. Circle size is proportional to the number of samples analysed, and circle colour indicates the reported mutation prevalence (%) according to the colour scale. The figure illustrates the marked geographic heterogeneity of the *Pfdhps* K540E mutation, with consistently higher prevalence observed in Eastern and Southern Africa than in most countries in West and Central Africa

The *Pfdhps* K540E mutation showed the most pronounced regional contrast, with a strong and persistent spatial–temporal pattern. In East and Southern Africa, K540E reached high and sustained prevalence, frequently exceeding 60–80% and in some settings approaching fixation (>90%). The pooled prevalence in East Africa was 99% (95% CI: 92–100%; I^2^ = 41.9%) in 2010 and 94% (95% CI: 83–98%; I^2^ = 90.8%) in 2023. Uganda consistently reported high prevalence from 2010 onwards (70–95%), while Kenya showed persistently elevated levels (80–100%) over more than a decade. In Tanzania, prevalence increased over time, reaching 70–90% in 2020–2023, and similarly high levels (60–85%) were observed in Rwanda, Malawi, and Zambia, which consistently report prevalence above 70%. In contrast, West and Central Africa exhibited substantially lower and more variable prevalence. In Central Africa, the pooled prevalence increased from 3% (1–7%; I^2^ = 0%) in 2010 to 10% (95% CI: 1–49%; I^2^ = 94.8%) in 2021, whereas it remained low at 1% (95% CI: 0–20%; I^2^ = 92.2%) in 2021 in West Africa. Ghana, Nigeria, and Burkina Faso consistently reported prevalence below 5%, whereas low to moderate levels (10–40%) were observed in the Central African Republic (2018: 10%; 15/157), Gabon (2016: 36%; 74/207), and the Republic of Congo (2021: 43%; 140/327). A notable exception was the Democratic Republic of Congo, where K540E prevalence increased sharply to 92% (435/475) in 2023 (Fig. 11; Supplementary 7; Supplementary 8).

**Fig. 12:**
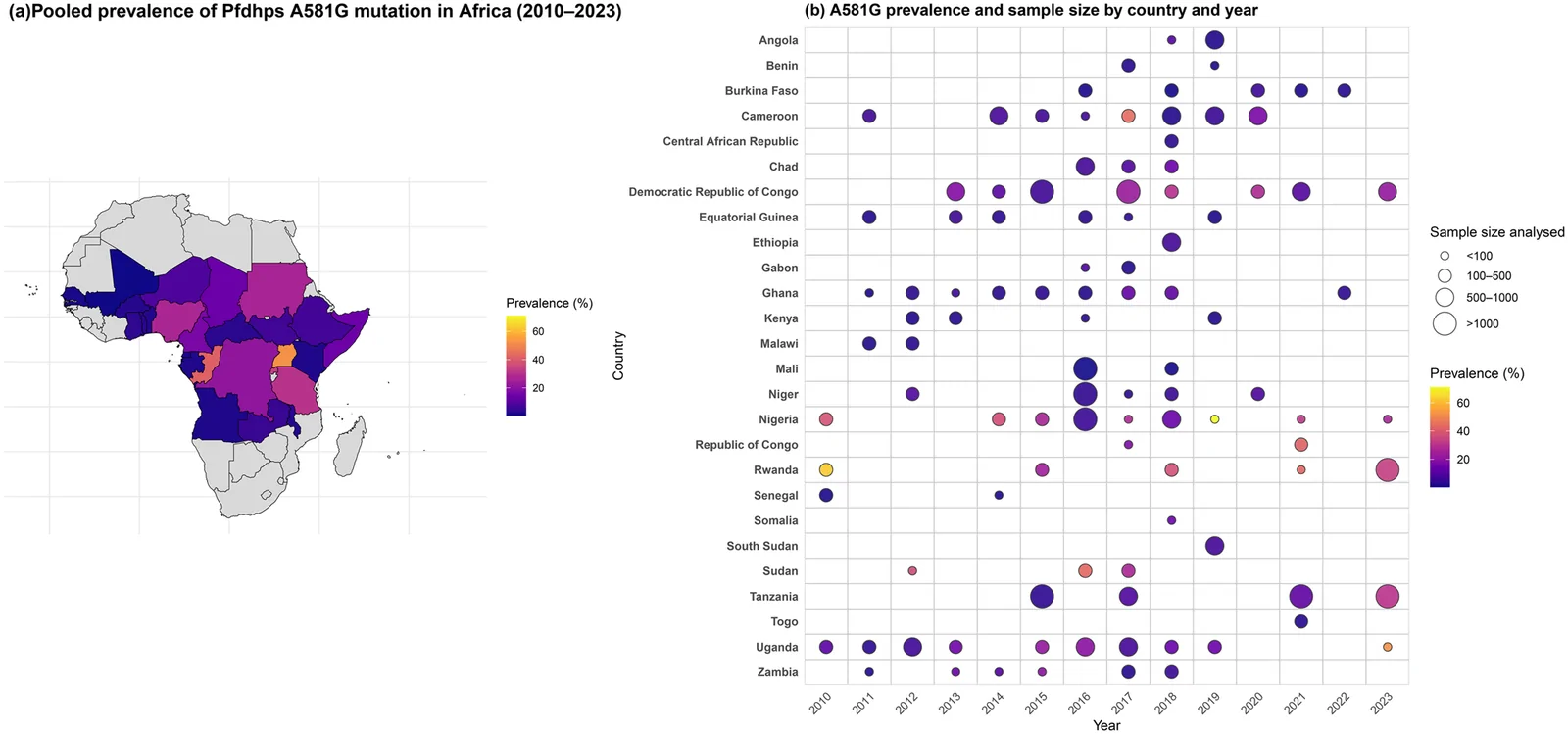
Spatial distribution and country-level temporal trends in the pooled prevalence of the *Pfdhps* A581G mutation across sub-Saharan Africa from 2010 to 2023. (**a**) Geographic distribution of the pooled prevalence of the *Pfdhps* A581G mutation by country. Countries with available data are coloured according to the pooled prevalence (%) shown on the accompanying colour scale, whereas countries without eligible data are shown in grey. (**b**) Country-level temporal distribution of the *Pfdhps* A581G mutation from 2010 to 2023. Each circle represents a country-year observation. Circle size is proportional to the number of samples analysed, and circle colour indicates the reported mutation prevalence (%) according to the colour scale. The figure illustrates the heterogeneous distribution of the *Pfdhps* A581G mutation across sub-Saharan Africa, with higher pooled prevalences observed in several East African countries and lower prevalences in most West and Central African countries during the study period

Finally, *Pfdhps* A581G displayed spatial clustering and heterogeneous temporal dynamics. Although generally detected at low to moderate prevalence (10–25%), higher and persistent levels (30–50%) were observed in a limited number of settings, particularly in East Africa in 2023, including Rwanda (33%; 861/2610), Tanzania (30%; 465/1534), and Uganda (52%; 38/73). Rwanda consistently exhibited elevated prevalence, with recurrent levels of 40–60%, peaking in 2010 at 63% (66/104), whereas Tanzania and Uganda showed greater interannual variability (10–40%). In the Democratic Republic of Congo, A581G was repeatedly detected at a moderate level (20–45%). Spatial analysis highlighted higher concentrations in East and Central Africa. At the same time, West African countries such as Ghana, Burkina Faso, and Senegal generally reported low prevalence (<10%), with Nigeria representing an exception, showing persistent moderate prevalence (20–40%) over time. The pooled prevalence of *Pfdhps* A581G in East Africa increased from 1% (95% CI: 0–63%; I2 = 94.7%) in 2010 to 36% (95% CI: 28–46%; I2 Fig. 12; Supplementary 7; Supplementary 8).

## Discussion

This systematic review synthesises the spatial and temporal dynamics of *Plasmodium falciparum* molecular markers associated with antimalarial drug resistance across sub-Saharan Africa between 2010 and 2024. Validated *PfKelch*13 mutations associated with artemisinin partial resistance were first identified in East Africa and increased over time, predominantly involving R561H, R622I, and A675V. These mutations were subsequently reported in Central Africa, whereas detections in West Africa remained limited and sporadic.

The emergence of molecular markers of artemisinin partial resistance should be interpreted in close relation to trends in ACT efficacy. Treatment failure of ACTs, particularly AL, the most widely used ACT in Africa, has been reported at several sentinel sites across the continent. In West Africa, AL efficacy below 90% has been documented in Mali (2016) [55], Burkina Faso (2018) [56], Nigeria (2020) [57], and Niger (2022) [58]. Similar findings have been reported in Central Africa, including the Democratic Republic of Congo (2017) [59] and Angola (2019) [60], as well as in East Africa, notably in Kenya (2017) [61] and Uganda (2019) [62]. When considered alongside these findings, our spatio-temporal analysis suggests that increases in validated *PfKelch13* mutations associated with artemisinin partial resistance have generally occurred in the same regions and periods where reduced AL efficacy has been reported, particularly in East and Central Africa. However, this observation is based on ecological comparisons rather than linked genotype-treatment outcome data and should therefore be interpreted with caution. In contrast, this pattern was less apparent in West Africa. The WHO defines mutations as validated markers of artemisinin partial resistance when two criteria are fulfilled. First, there must be a statistically significant association between a *PfKelch13* mutation and delayed parasite clearance. Second, the mutation must be associated with either more than 1% survival in the in vitro ring stage survival assay (RSA)^0-3h^ in at least five isolates or a statistically significant difference in the RSA^0-3h^ assay between culture-adapted recombinant isogenic parasite lines expressing the mutant allele compared with those expressing the wild-type [12]. Indeed, the validated *PfKelch13* markers have been confirmed to be associated with the artemisinin partial resistance phenotype characterized by delayed parasite clearance following treatment with ACTs or artemisinin monotherapy. In Southeast Asia, validated *PfKelch13* mutations, such as C580Y and R539T, are frequently reported [63]. In contrast, these mutations remain rare in Africa (in our systematic review, only five cases were identified, all from Ghana and Nigeria). Although R561H was initially described in Southeast Asia, its prevalence is now increasing in several African countries, particularly in Rwanda and Tanzania. Conversely, mutations such as Y493H and F446I, which are largely found in Southeast Asia, were not observed in Africa [63–65]. In the same line, other validated mutations, including R622I and C469Y, seem to have emerged independently in East Africa rather than being imported from Asia. These observations suggest that artemisinin partial resistance in Africa is not simply the result of the spread of Southeast Asian lineages but reflects local evolutionary dynamics under distinct epidemiological and transmission conditions. The independent emergence and spread of validated *PfKelch13* mutations across different African regions underscores the need to characterise artemisinin partial resistance within the specific African context and to adapt surveillance and control strategies accordingly.

Delayed parasite clearance, the key clinical phenotype of artemisinin partial resistance, has been repeatedly reported in East Africa and shown to be associated with validated molecular markers. Notably, ring-stage survival assays demonstrated that the *PfKelch13* R561H mutation confers reduced susceptibility to dihydroartemisinin, providing the first definitive evidence of artemisinin partial resistance on the African continent [13]. Confirmed artemisinin partial resistance has been documented in Eritrea (R622I) [66], Rwanda (R561H) [67], Uganda (A675V, C469Y) [68, 69], and Tanzania (R561H) [70], where delayed parasite clearance has been observed alongside validated *PfKelch13*, resistance markers. In addition, the detection of validated or candidate mutations at prevalences exceeding 5% at specific sites, has been reported in Ethiopia (R622I) [71, 72], Namibia (P441L) [73], Sudan (R622I) [74], and Zambia (P441L) [75], but not associated with the delay in parasite clearance. The emergence and spread of artemisinin partial resistance represent a significant threat to malaria control and elimination efforts in Africa. This challenge is further compounded by the widespread increase in the *Pfmdr1* NFD haplotype, which is associated with reduced susceptibility to lumefantrine [34, 76, 77] and is likely contributing to the declining efficacy of AL across the continent. Collectively, these findings highlight the urgent need to strengthen molecular surveillance and to explore alternative treatment and resistance-mitigation strategies to safeguard malaria control gains in Africa.

Distinct regional patterns of antimalarial drug resistance are evident in West Africa compared with other African regions. This divergence is particularly apparent for molecular markers of artemisinin partial resistance, as validated *PfKelch13* mutations have been reported in East and Central Africa but remain largely absent in West Africa, despite documented declines in AL efficacy in both regions. Moreover, the observed spatial clustering of *PfKelch13* mutations indicates that the emergence and expansion of these variants are occurring within specific regional hotspots, particularly in East Africa and the Horn of Africa, rather than through continent-wide dissemination. Similar regional contrasts are observed for markers of SP resistance. Indeed, over time, the *Pfdhps* K540E mutation, associated with the quintuple mutant haplotype conferring full SP resistance and clinical treatment failure [78–80], has approached fixation in East Africa and reached moderate prevalence in Central Africa, while remaining uncommon in West Africa. Conversely, the *Pfdhps* I431V mutation, whose contribution to SP resistance is not yet fully established [81], has been reported predominantly in West Africa and is largely absent in East Africa. These contrasting regional patterns highlight substantial differences in parasite genetic diversity across Africa and underscore the need to better understand the biological, epidemiological, and ecological drivers underlying resistance emergence and spread. Factors such as antimalarial drug use policies, transmission intensity, and environmental conditions are likely to shape regional molecular resistance profiles. The emergence and spread of drug-resistant parasites are shaped by parasite-related factors, including the level of resistance conferred by specific mutations, the parasite’s genetic background, and the fitness costs associated with resistance [82–85]. Environmental factors also play a key role, notably ecological conditions influencing transmission intensity, the competence of local mosquito vectors to transmit resistant strains, and their susceptibility to vector control interventions [86–91]. Moreover, transmission dynamics, such as transmission intensity, drug pressure, opportunities for genetic recombination, competition between susceptible and resistant parasites, and the contribution of host immunity to infection clearance, strongly influence the evolution of resistance [90, 92–96]. Accordingly, resistance-mitigation strategies should be region-specific and informed by local molecular epidemiology. Given the marked regional heterogeneity in resistance patterns across Africa, tailored, region-specific strategies with clearly defined objectives are required to improve the effectiveness of resistance containment efforts and to support malaria elimination goals. For example, given the distinct SP resistance profiles, it is critical to reassess the effectiveness of SP for intermittent preventive treatment in pregnancy (IPTp) and seasonal malaria chemoprevention (SMC) in different SP resistance settings. Although SP has remained effective in improving pregnancy outcomes, particularly birth weight, across a range of resistance contexts, including areas of East Africa with high prevalence of the quintuple mutation [97]. This fact emphasises the need for continuous evaluation of preventive strategies in the context of evolving resistance landscapes.

Furthermore, our systematic review demonstrated a significant continent-wide decline in the prevalence of the *Pfcrt* K76T and *Pfmdr1* N86Y mutations over time following the withdrawal of chloroquine. These temporal trends indicate a substantial reduction in chloroquine-resistant parasite populations after drug removal, suggesting that chloroquine could be reconsidered in combination with other antimalarial drugs under carefully monitored conditions. In addition, these findings support the continued use of ACT partner drugs that exert opposing selective pressure, particularly amodiaquine. In settings where AL efficacy has been reported to decline, ASAQ represents a viable alternative. Indeed, therapeutic efficacy studies conducted across multiple African countries have consistently shown high efficacy of ASAQ, with treatment failure rates remaining below 10%, supporting its sustained use as a first-line or alternative ACT [1, 3].

Overall, antimalarial drug resistance has become a significant public health concern in Africa due to its emergence and progressive spread, underscoring the need for strengthened and coordinated mitigation strategies. Continuous monitoring of ACT efficacy through therapeutic efficacy studies should constitute the cornerstone of resistance surveillance. The WHO recommends conducting therapeutic efficacy studies at sentinel sites in each country at least every two years to assess drug efficacy. However, since 2020, therapeutic efficacy studies data have been reported from only 15 African countries, highlighting substantial gaps in surveillance coverage [1]. Molecular surveillance of resistance markers should systematically complement therapeutic efficacy studies to enable early detection of emerging resistance and to inform treatment policy decisions [6]. In our meta-analysis, many molecular studies originated from East Africa, whereas data from West and Central Africa remain limited, restricting comprehensive continent-wide assessments. Sequencing-based methods have steadily expanded across African countries, marking a shift from non-sequencing approaches and from Sanger to high-throughput NGS technologies. Taken together, this marks an encouraging trend toward strengthening molecular surveillance of antimalarial drug resistance in sub-Saharan Africa.

This systematic review has some limitations. Grey literature sources were not systematically searched; therefore, relevant unpublished studies may have been missed. The analysis was conducted at the country level rather than at individual study sites, limiting the ability to capture finer within-country spatial and temporal trends. The aggregation of data at the country level may have masked subnational variation in mutation prevalence and localized resistance hotspots. Although this review aimed to describe spatio-temporal trends in antimalarial resistance markers across sub-Saharan Africa, analyses were conducted primarily at the country level because site-specific data were inconsistently reported and longitudinal data from the same locations were often unavailable. The use of a 5° neighbourhood threshold (approximately 550 km) was a pragmatic choice to ensure spatial connectivity but may link geographically distant sites. Therefore, the hotspot analysis should be interpreted cautiously because the included studies were not based on a standardized surveillance network, and site distribution varied substantially across countries and years. Consequently, identified hotspots reflect areas of elevated mutation prevalence among surveyed locations rather than definitive centers of resistance emergence or transmission. In addition, site-specific temporal trends could not be reliably assessed, and the spatial patterns presented should be interpreted as broad national and regional trends rather than precise local estimates. Although the meta-regression identified significant temporal and regional associations, substantial residual heterogeneity remained (I^2^ > 97%), indicating that additional unmeasured factors may have influenced the observed prevalence. Therefore, the estimated odds ratios should be interpreted as overall trends rather than precise effect estimates.

Other limitations include the possibility that publication bias may have influenced the available evidence, as studies reporting unusual or emerging resistance patterns may be more likely to be published. The review was restricted to studies published in English and French, potentially introducing language bias. Surveillance intensity varied considerably across sub-Saharan Africa, with some countries and regions contributing substantially more data than others. Differences in study design, sampling strategies, and target populations may have affected the comparability of prevalence estimates across studies. Despite careful screening, the possibility of overlapping study populations or duplicate sample reporting cannot be entirely excluded. These limitations may have influenced the pooled prevalence estimates and the interpretation of spatio-temporal trends. Although most studies were assessed as having a low risk of bias, this does not necessarily imply high comparability across studies, as methodological and clinical heterogeneity remained.

However, such methodological differences are widespread and are unlikely to have substantially affected the overall regional and temporal patterns observed. Furthermore, because data extraction, cleaning, and synthesis were time-intensive, a small number of very recent publications may not have been included. Nevertheless, the dataset spans 15 years (2010–2024) and provides evidence consistent with long-term trends in the prevalence of molecular markers associated with antimalarial drug resistance. Unlike previous reviews and WHO surveillance reports, our study quantitatively evaluated temporal trends through meta-regression analysis and provided a detailed assessment of the evolution of individual resistance mutations and haplotypes. By synthesizing data from a large number of studies, this work offers a broader regional perspective on the dynamics of antimalarial drug resistance and complements existing surveillance efforts.

## Conclusion

This systematic review provides a broad overview of the major molecular markers associated with antimalarial drug resistance across sub-Saharan Africa over 15 years (2010–2024), while highlighting important geographical variations and gaps in surveillance coverage. The prevalence of resistance-associated mutations shows clear temporal dynamics and distinct spatial patterns across regions, underscoring the growing challenge that antimalarial resistance represents for malaria control in Africa. These findings highlight the need for region-specific strategies aligned with the local epidemiology of molecular markers of antimalarial drug resistance, supported by continuous molecular surveillance.

## Electronic supplementary material

Below is the link to the electronic supplementary material.

Supplementary material 1

## Data Availability

The datasets used and/or analyzed during the current study are presented within the manuscript and additional supporting files and are also available from the corresponding author on reasonable request.

## Abbreviations

ACT: Artemisinin-based combination therapy
AL: Artemether–lumefantrine
SP: sulfadoxine-pyrimethamine
*Pfcrt*: *Plasmodium falciparum chloroquine resistance transporter*
*Pfmdr1*: *Plasmodium falciparum multidrug resistance 1*
*Pfdhfr*: *Plasmodium falciparum dihydrofolate reductase*
*Pfdhps*: *Plasmodium falciparum dihydropteroate synthase*
*PfKelch13*: *Plasmodium falciparum Kelch13*
PCR: Polymerase chain reaction
NGS: Next Generation Sequencing
WHO: World Health Organisation
PRISMA: Preferred reporting items for systematic review and meta-analysis
