# Supplementary material for: Spatio-temporal trends of the prevalence of Plasmodium falciparum antimalarial drug resistance markers across sub-Saharan Africa from 2010 to 2024: a systematic review and meta-analysis
Source: BMC Infect Dis. 2026 Sep 3;26:1605. doi: 10.1186/s12879-026-14233-2 (PMC13545778; doi:10.1186/s12879-026-14233-2)
Supplement: Supplementary file 1 [file 12879_2026_14233_MOESM1_ESM.zip › Supplementary materials_15.07.2026/Supplementary_01_Search equation.docx]

**Supplementary document 1**

# Equations

- **Scopus**

( TI-AB-AK ( antimalarial  OR  artemisinin  OR  artesunate  OR  artemether  OR  lumefantrine  OR  amodiaquine  OR  sulfadoxine  OR  pyrimethamine  OR  dihydroartemisinin  OR  piperaquine  OR  mefloquine  OR  pyronaridine )  AND  TI-AB-AK ( plasmodium  OR  "plasmodium falciparum"  OR  malaria  OR  paludism )  AND  TI-AB-AK ( "Africa South of the Sahara"  OR  "Africa, Central"  OR  "Cameroon"  OR  "Central African Republic"  OR  "Chad"  OR  "Congo"  OR  "Democratic Republic of the Congo"  OR  "Equatorial Guinea"  OR  "Gabon"  OR  "Sao Tome and Principe"  OR  "Africa, Eastern"  OR  "Burundi"  OR  "Djibouti"  OR  "Eritrea"  OR  "Ethiopia"  OR  "Kenya"  OR  "Rwanda"  OR  "Somalia"  OR  "South Sudan"  OR  "Sudan"  OR  "Tanzania"  OR  "Uganda"  OR  "Africa, Southern"  OR  "Angola"  OR  "Botswana"  OR  "Eswatini"  OR  "Lesotho"  OR  "Malawi"  OR  "Mozambique"  OR  "Namibia"  OR  "South Africa"  OR  "Zambia"  OR  "Zimbabwe"  OR  "Africa, Western"  OR  "Benin"  OR  "Burkina Faso"  OR  "Cabo Verde"  OR  "Cote d'Ivoire"  OR  "Gambia"  OR  "Ghana"  OR  "Guinea"  OR  "Guinea-Bissau"  OR  "Liberia"  OR  "Mali"  OR  "Mauritania"  OR  "Niger"  OR  "Nigeria"  OR  "Senegal"  OR  "Sierra Leone"  OR  "Togo" )  AND  TITLE-ABS-KEY ( efficac*  OR  treatmen*  OR  therap*  OR  "failure"  OR  "treatment failure"  OR  "effect"  OR  "effecting"  OR  "effective"  OR  "effectively"  OR  "effectiveness"  OR  "effectivenesses"  OR  "effectives"  OR  "effectivities"  OR  "effectivity"  OR  "effects"  OR  "molecular markers "  OR  " plasmodium falciparum chloroquine resistance transporter"  OR  "pfcrt"  OR  "plasmodium falciparum multi drug resistance"  OR  "pfmdr"  OR  "plasmodium falciparum dihydrofolate reductase"  OR  "pfdhfr"  OR  "plasmodium falciparum dihydrofolate synthase"  OR  "pfdhps"  OR  "plasmodium falciparum kelch 13"  OR  "pfk13"  OR  "plasmodium falciparum plasmepsin 2"  OR  "pfpm2" ) )  AND  ( LIMIT-TO ( DOCTYPE ,  "ar" )  OR  LIMIT-TO ( DOCTYPE ,  "sh" ) )  AND  (LIMIT-TO ( PUBYEAR ,  2024 ))

- **Pubmed / MEDLINE**

((("Plasmodium"[All Fields] OR ("Plasmodium"[All Fields] AND "falciparum"[All Fields])) AND ("Antimalarial"[All Fields] OR ("Malaria"[All Fields] AND "drug"[All Fields])) AND ("resistance"[All Fields] OR ("molecular"[All Fields] AND "markers"[All Fields]) OR "Pfcrt"[All Fields] OR ("chloroquine"[All Fields] AND "resistance"[All Fields] AND "transporter"[All Fields]) OR "pfmdr1"[All Fields] OR "pfK13"[All Fields] OR "Pfdhps"[All Fields] OR "Pfdhfr"[All Fields] OR "pfpm"[All Fields]) AND ("Africa"[All Fields] OR ("subSaharan"[All Fields] AND "Africa"[All Fields]))) AND "2"[All Fields] AND 2024/01/01:2024/12/31[Date - Publication]) NOT (systematic review[Publication Type])

- **Web of Science**

((((ALL=(antimalarial OR artemisinin OR artesunate OR artemether OR lumefantrine OR amodiaquine OR sulfadoxine OR pyrimethamine OR dihydroartemisinin OR piperaquine OR mefloquine OR pyronaridine )) AND ALL=(plasmodium OR "plasmodium falciparum" OR malaria OR paludisme )) AND ALL=("Africa South of the Sahara" OR "Africa, Central" OR "Cameroon" OR "Central African Republic" OR "Chad" OR "Congo" OR "Democratic Republic of the Congo" OR "Equatorial Guinea" OR "Gabon" OR "Sao Tome and Principe" OR "Africa, Eastern" OR "Burundi" OR "Djibouti" OR "Eritrea" OR "Ethiopia" OR "Kenya" OR "Rwanda" OR "Somalia" OR "South Sudan" OR "Sudan" OR "Tanzania" OR "Uganda" OR "Africa, Southern" OR "Angola" OR "Botswana" OR "Eswatini" OR "Lesotho" OR "Malawi" OR "Mozambique" OR "Namibia" OR "South Africa" OR "Zambia" OR "Zimbabwe" OR "Africa, Western" OR "Benin" OR "Burkina Faso" OR "Cabo Verde" OR "Cote d'Ivoire" OR "Gambia" OR "Ghana" OR "Guinea" OR "Guinea-Bissau" OR "Liberia" OR "Mali" OR "Mauritania" OR "Niger" OR "Nigeria" OR "Senegal" OR "Sierra Leone" OR "Togo" )) AND ALL=(efficac* OR treatmen* OR therap* OR "failure" OR "treatment failure" OR "effect" OR "effecting" OR "effective" OR "effectively" OR "effectiveness" OR "effectivenessas" OR "effectivess" OR "effectivites" OR "effectivity" OR "effects" OR "molecular markers " OR " plasmodium falciparum chloroquine resistance transporter" OR "pfcrt" OR "plasmodium falciparum multi drug resistance" OR "pfmdr1" OR "plasmodium falciparum dihydrofolate reductase" OR "Pfdhfr" OR "plasmodium falciparum dihydrofolate synthase" OR "pfdhps" OR "plasmodium falciparum kelch 13" OR "pfk13" OR "plasmodium falciparum plasmepsin 2" OR "pfpm2" )) AND DOP=(2024-01-01/2024-12-31)

- **Embase**

('plasmodium falciparum'/exp OR 'Plasmodium falciparum' OR malaria) AND ('drug resistance'/exp OR 'antimalarial drug resistance' OR 'malaria drug resistance' OR 'drug resistance, malaria') AND (pfcrt OR 'chloroquine resistance transporter' OR 'P. falciparum chloroquine resistance transporter' OR pfmdr1 OR 'multidrug resistance 1' OR 'P. falciparum multidrug resistance 1' OR pfkelch13 OR kelch13 OR k13 OR 'kelch propeller domain' OR 'kelch 13' OR pfdhfr OR 'dihydrofolate reductase' OR pfdhps OR 'dihydropteroate synthase') AND (mutation* OR polymorphism* OR variant* OR genotype* OR haplotype* OR allele* OR marker* OR 'molecular marker*' OR surveillance OR prevalence) AND ('africa'/exp OR Africa OR 'sub-Saharan Africa' OR 'West Africa' OR 'East Africa' OR 'Central Africa' OR 'Southern Africa' OR Benin OR Burkina Faso OR Cabo Verde OR Cameroon OR 'Central African Republic' OR Chad OR Congo OR 'Democratic Republic of the Congo' OR Djibouti OR 'Equatorial Guinea' OR Eritrea OR Eswatini OR Ethiopia OR Gabon OR Gambia OR Ghana OR Guinea OR 'Guinea-Bissau' OR Kenya OR Lesotho OR Liberia OR Madagascar OR Malawi OR Mali OR Mauritania OR Mauritius OR Mozambique OR Namibia OR Niger OR Nigeria OR Rwanda OR Senegal OR Seychelles OR 'Sierra Leone' OR Somalia OR 'South Africa' OR 'South Sudan' OR Sudan OR Tanzania OR Togo OR Tunisia OR Uganda OR Zambia OR Zimbabwe) AND [2010-2024]/py
