# Supplementary material for: Spatio-temporal trends of the prevalence of Plasmodium falciparum antimalarial drug resistance markers across sub-Saharan Africa from 2010 to 2024: a systematic review and meta-analysis
Source: BMC Infect Dis. 2026 Sep 3;26:1605. doi: 10.1186/s12879-026-14233-2 (PMC13545778; doi:10.1186/s12879-026-14233-2)
Supplement: Supplementary file 1 [file 12879_2026_14233_MOESM1_ESM.zip › Supplementary materials_15.07.2026/Supplementary_03_Summary studies included and sequencing methods.docx]

**Supplementary document 3**

# Table 1: Descriptive analysis of studies included in the meta-analysis from 2010 to 2024

| Year of publication (N=352) | n | % |
| --- | --- | --- |
| 2011 | 2 | 0.6 |
| 2012 | 5 | 1.4 |
| 2013 | 12 | 3.4 |
| 2014 | 16 | 4.5 |
| 2015 | 28 | 8.0 |
| 2016 | 25 | 7.1 |
| 2017 | 25 | 7.1 |
| 2018 | 27 | 7.7 |
| 2019 | 30 | 8.5 |
| 2020 | 43 | 12.2 |
| 2021 | 42 | 11.9 |
| 2022 | 21 | 6.0 |
| 2023 | 40 | 11.4 |
| 2024 | 36 | 10.2 |

- **Table 2**: Studies on *Pfcrt* included in the meta-analysis per year and per country

| **Country** | **2010** | **2011** | **2012** | **2013** | **2014** | **2015** | **2016** | **2017** | **2018** | **2019** | **2020** | **2021** | **2022** | **2023** | **Total** |
| --- | --- | --- | --- | --- | --- | --- | --- | --- | --- | --- | --- | --- | --- | --- | --- |
| Angola | 0 | 1 | 0 | 0 | 0 | 0 | 0 | 1 | 1 | 1 | 0 | 0 | 0 | 0 | 4 |
| Benin | 0 | 1 | 1 | 0 | 0 | 0 | 0 | 0 | 0 | 1 | 0 | 0 | 0 | 0 | 3 |
| Botswana | 0 | 0 | 0 | 0 | 0 | 0 | 1 | 0 | 0 | 0 | 0 | 0 | 0 | 0 | 1 |
| Burkina Faso | 0 | 0 | 2 | 0 | 0 | 0 | 1 | 0 | 1 | 0 | 0 | 1 | 1 | 0 | 6 |
| Cameroon | 0 | 0 | 2 | 0 | 2 | 0 | 0 | 1 | 1 | 1 | 3 | 0 | 0 | 0 | 10 |
| Cape Verde | 0 | 0 | 0 | 0 | 0 | 0 | 0 | 1 | 0 | 0 | 0 | 0 | 0 | 0 | 1 |
| Chad | 0 | 0 | 0 | 0 | 0 | 0 | 2 | 1 | 1 | 0 | 0 | 1 | 0 | 0 | 5 |
| Congo | 2 | 0 | 0 | 1 | 0 | 2 | 0 | 0 | 0 | 0 | 0 | 0 | 0 | 0 | 5 |
| Côte d'Ivoire | 0 | 0 | 0 | 0 | 1 | 1 | 0 | 0 | 0 | 0 | 0 | 0 | 0 | 0 | 2 |
| Democratic Republic of Congo | 1 | 0 | 0 | 1 | 2 | 0 | 0 | 2 | 0 | 3 | 1 | 1 | 0 | 0 | 11 |
| Equatorial Guinea | 0 | 1 | 0 | 2 | 1 | 0 | 1 | 0 | 0 | 2 | 0 | 0 | 0 | 0 | 7 |
| Eritrea | 0 | 0 | 0 | 0 | 1 | 0 | 0 | 0 | 0 | 0 | 0 | 0 | 0 | 0 | 1 |
| Ethiopia | 1 | 2 | 1 | 1 | 4 | 0 | 1 | 1 | 1 | 0 | 1 | 1 | 0 | 0 | 14 |
| Gabon | 0 | 1 | 1 | 1 | 3 | 0 | 0 | 0 | 0 | 0 | 0 | 0 | 0 | 0 | 6 |
| Gambia | 0 | 0 | 2 | 2 | 2 | 1 | 1 | 0 | 1 | 0 | 0 | 0 | 0 | 0 | 9 |
| Ghana | 2 | 2 | 4 | 0 | 2 | 1 | 1 | 2 | 2 | 0 | 0 | 0 | 1 | 0 | 17 |
| Guinea | 0 | 0 | 0 | 0 | 0 | 0 | 1 | 0 | 1 | 0 | 0 | 0 | 0 | 0 | 2 |
| Guinea-Bissau | 1 | 1 | 1 | 0 | 1 | 1 | 2 | 0 | 1 | 0 | 0 | 0 | 0 | 0 | 8 |
| Kenya | 3 | 2 | 3 | 4 | 1 | 3 | 2 | 2 | 1 | 1 | 0 | 0 | 0 | 0 | 22 |
| Liberia | 0 | 0 | 0 | 0 | 0 | 0 | 0 | 0 | 1 | 0 | 0 | 0 | 0 | 0 | 1 |
| Madagascar | 0 | 0 | 0 | 0 | 0 | 0 | 1 | 1 | 0 | 0 | 0 | 0 | 0 | 0 | 2 |
| Malawi | 0 | 0 | 1 | 0 | 0 | 0 | 0 | 0 | 0 | 0 | 0 | 0 | 0 | 0 | 1 |
| Mali | 0 | 0 | 1 | 1 | 3 | 1 | 5 | 0 | 1 | 0 | 1 | 0 | 0 | 0 | 13 |
| Mauritania | 1 | 0 | 0 | 0 | 0 | 0 | 0 | 0 | 0 | 0 | 0 | 0 | 0 | 0 | 1 |
| Mozambique | 2 | 0 | 0 | 0 | 0 | 3 | 0 | 1 | 1 | 0 | 0 | 0 | 0 | 0 | 7 |
| Niger | 0 | 0 | 0 | 0 | 0 | 0 | 1 | 0 | 1 | 0 | 0 | 0 | 0 | 0 | 2 |
| Nigeria | 0 | 1 | 0 | 0 | 2 | 2 | 3 | 1 | 1 | 1 | 0 | 0 | 0 | 0 | 11 |
| Rwanda | 1 | 0 | 0 | 0 | 0 | 1 | 0 | 0 | 0 | 0 | 0 | 1 | 0 | 0 | 3 |
| Sao Tome and Principe | 0 | 0 | 0 | 0 | 1 | 1 | 1 | 0 | 0 | 0 | 0 | 0 | 0 | 0 | 3 |
| Senegal | 3 | 3 | 2 | 2 | 4 | 0 | 0 | 0 | 0 | 0 | 0 | 0 | 0 | 0 | 14 |
| Sierra Leone | 0 | 0 | 0 | 0 | 0 | 0 | 0 | 0 | 1 | 0 | 0 | 0 | 0 | 0 | 1 |
| Somalia | 0 | 0 | 0 | 0 | 0 | 0 | 0 | 0 | 1 | 0 | 0 | 0 | 0 | 0 | 1 |
| South Africa | 1 | 1 | 1 | 0 | 0 | 0 | 0 | 0 | 1 | 0 | 0 | 0 | 0 | 0 | 4 |
| South Sudan | 0 | 0 | 0 | 0 | 0 | 0 | 0 | 0 | 0 | 1 | 0 | 0 | 0 | 0 | 1 |
| Sudan | 0 | 0 | 1 | 0 | 0 | 0 | 2 | 1 | 1 | 0 | 0 | 0 | 0 | 0 | 5 |
| Uganda | 4 | 3 | 4 | 4 | 5 | 4 | 3 | 4 | 2 | 1 | 0 | 1 | 0 | 0 | 35 |
| Tanzania | 1 | 3 | 1 | 1 | 2 | 0 | 0 | 1 | 0 | 1 | 0 | 0 | 0 | 0 | 10 |
| Zambia | 0 | 0 | 1 | 0 | 0 | 1 | 2 | 1 | 0 | 0 | 0 | 0 | 0 | 0 | 5 |
| Zanzibar | 1 | 0 | 0 | 0 | 0 | 0 | 0 | 1 | 0 | 0 | 0 | 0 | 0 | 0 | 2 |
| Zimbabwe | 0 | 0 | 0 | 1 | 0 | 0 | 0 | 0 | 1 | 0 | 0 | 0 | 0 | 0 | 2 |

- **Table 3:** Studies on *Pfmdr1* included in the meta-analysis per year and per country

| **Country** | **2010** | **2011** | **2012** | **2013** | **2014** | **2015** | **2016** | **2017** | **2018** | **2019** | **2020** | **2021** | **2022** | **2023** | **Total** |
| --- | --- | --- | --- | --- | --- | --- | --- | --- | --- | --- | --- | --- | --- | --- | --- |
| Angola | 0 | 2 | 1 | 1 | 0 | 1 | 0 | 1 | 1 | 1 | 0 | 0 | 0 | 0 | 8 |
| Benin | 0 | 1 | 1 | 0 | 0 | 1 | 0 | 0 | 0 | 1 | 0 | 0 | 0 | 0 | 4 |
| Botswana | 0 | 0 | 0 | 0 | 0 | 0 | 1 | 0 | 0 | 0 | 0 | 0 | 0 | 0 | 1 |
| Burkina Faso | 0 | 0 | 2 | 0 | 0 | 1 | 2 | 0 | 2 | 0 | 0 | 1 | 1 | 0 | 9 |
| Cameroon | 0 | 0 | 0 | 0 | 2 | 0 | 0 | 1 | 1 | 1 | 3 | 0 | 0 | 0 | 8 |
| Cape Verde | 0 | 0 | 0 | 0 | 0 | 0 | 0 | 1 | 0 | 0 | 0 | 0 | 0 | 0 | 1 |
| Chad | 0 | 0 | 0 | 0 | 0 | 0 | 2 | 1 | 1 | 0 | 0 | 1 | 0 | 0 | 5 |
| Congo | 2 | 0 | 0 | 1 | 0 | 3 | 0 | 0 | 0 | 0 | 0 | 0 | 0 | 0 | 6 |
| Democratic Republic of Congo | 0 | 0 | 1 | 0 | 2 | 1 | 0 | 1 | 0 | 2 | 1 | 1 | 0 | 0 | 9 |
| Equatorial Guinea | 0 | 1 | 1 | 3 | 1 | 0 | 1 | 0 | 0 | 2 | 0 | 0 | 0 | 0 | 9 |
| Eritrea | 0 | 0 | 0 | 0 | 1 | 0 | 0 | 0 | 0 | 0 | 0 | 0 | 0 | 0 | 1 |
| Ethiopia | 1 | 1 | 0 | 1 | 2 | 0 | 1 | 2 | 1 | 0 | 1 | 0 | 0 | 0 | 10 |
| Gabon | 0 | 0 | 1 | 0 | 2 | 1 | 0 | 0 | 1 | 0 | 0 | 0 | 0 | 0 | 5 |
| Gambia | 0 | 0 | 1 | 1 | 1 | 1 | 1 | 0 | 1 | 0 | 0 | 0 | 0 | 0 | 6 |
| Ghana | 2 | 1 | 4 | 1 | 2 | 1 | 2 | 2 | 3 | 0 | 0 | 0 | 1 | 0 | 19 |
| Guinea | 0 | 0 | 0 | 0 | 0 | 0 | 2 | 0 | 1 | 0 | 0 | 0 | 0 | 0 | 3 |
| Guinea-Bissau | 1 | 1 | 1 | 0 | 1 | 1 | 1 | 0 | 1 | 0 | 0 | 0 | 0 | 0 | 7 |
| Kenya | 2 | 2 | 4 | 4 | 1 | 1 | 2 | 2 | 1 | 1 | 0 | 0 | 0 | 0 | 20 |
| Liberia | 0 | 0 | 0 | 0 | 0 | 0 | 0 | 0 | 1 | 0 | 0 | 0 | 0 | 0 | 1 |
| Madagascar | 0 | 0 | 0 | 0 | 0 | 0 | 1 | 1 | 0 | 0 | 0 | 0 | 0 | 0 | 2 |
| Mali | 0 | 0 | 1 | 0 | 3 | 1 | 6 | 0 | 1 | 0 | 1 | 0 | 0 | 0 | 13 |
| Mauritania | 1 | 0 | 0 | 0 | 0 | 0 | 0 | 0 | 0 | 0 | 0 | 0 | 0 | 0 | 1 |
| Mozambique | 2 | 0 | 1 | 0 | 0 | 3 | 0 | 1 | 1 | 0 | 0 | 0 | 0 | 0 | 8 |
| Niger | 0 | 0 | 1 | 0 | 0 | 0 | 1 | 1 | 1 | 0 | 0 | 0 | 0 | 0 | 4 |
| Nigeria | 0 | 1 | 0 | 0 | 2 | 2 | 3 | 0 | 2 | 1 | 0 | 0 | 0 | 0 | 11 |
| Rwanda | 1 | 0 | 0 | 0 | 0 | 1 | 0 | 0 | 1 | 1 | 0 | 1 | 0 | 0 | 5 |
| Sao Tome and Principe | 0 | 0 | 0 | 0 | 1 | 1 | 1 | 0 | 0 | 0 | 0 | 0 | 0 | 0 | 3 |
| Senegal | 2 | 1 | 1 | 1 | 3 | 1 | 1 | 1 | 0 | 0 | 0 | 0 | 0 | 0 | 11 |
| Sierra Leone | 0 | 0 | 0 | 0 | 0 | 0 | 0 | 0 | 1 | 0 | 0 | 0 | 0 | 0 | 1 |
| Somalia | 0 | 0 | 0 | 0 | 0 | 0 | 0 | 0 | 1 | 0 | 0 | 0 | 0 | 0 | 1 |
| South Africa | 0 | 1 | 0 | 0 | 0 | 0 | 0 | 0 | 1 | 0 | 0 | 0 | 0 | 0 | 2 |
| South Sudan | 0 | 0 | 0 | 0 | 0 | 0 | 0 | 0 | 0 | 1 | 0 | 0 | 0 | 0 | 1 |
| Sudan | 0 | 0 | 1 | 0 | 0 | 0 | 1 | 2 | 2 | 0 | 0 | 0 | 0 | 0 | 6 |
| Uganda | 3 | 3 | 5 | 3 | 5 | 5 | 4 | 4 | 2 | 2 | 0 | 1 | 0 | 1 | 38 |
| Tanzania | 2 | 3 | 0 | 1 | 2 | 0 | 1 | 1 | 1 | 1 | 0 | 0 | 0 | 0 | 12 |
| Zambia | 0 | 0 | 1 | 0 | 0 | 1 | 1 | 1 | 0 | 0 | 0 | 0 | 0 | 0 | 4 |
| Zanzibar | 1 | 0 | 0 | 0 | 0 | 0 | 0 | 1 | 0 | 0 | 0 | 0 | 0 | 0 | 2 |

- **Table 4:** Studies on *PfKelch13* included in the meta-analysis per year and per country

| **Country** | **2010** | **2011** | **2012** | **2013** | **2014** | **2015** | **2016** | **2017** | **2018** | **2019** | **2020** | **2021** | **2022** | **2023** | **Total** |
| --- | --- | --- | --- | --- | --- | --- | --- | --- | --- | --- | --- | --- | --- | --- | --- |
| Angola | 0 | 1 | 1 | 1 | 0 | 2 | 0 | 1 | 1 | 1 | 0 | 0 | 0 | 0 | 8 |
| Benin | 0 | 0 | 0 | 0 | 1 | 1 | 0 | 1 | 0 | 2 | 0 | 0 | 0 | 0 | 5 |
| Botswana | 0 | 0 | 0 | 0 | 0 | 0 | 1 | 0 | 0 | 0 | 0 | 0 | 0 | 0 | 1 |
| Burkina Faso | 0 | 0 | 1 | 0 | 0 | 1 | 0 | 0 | 1 | 0 | 0 | 1 | 0 | 0 | 4 |
| Cameroon | 0 | 0 | 0 | 0 | 3 | 0 | 2 | 2 | 1 | 0 | 1 | 0 | 0 | 0 | 9 |
| Cape Verde | 0 | 0 | 0 | 0 | 0 | 0 | 0 | 1 | 0 | 0 | 0 | 0 | 0 | 0 | 1 |
| Central African Republic | 0 | 0 | 0 | 0 | 0 | 0 | 0 | 0 | 0 | 1 | 0 | 0 | 0 | 0 | 1 |
| Chad | 0 | 0 | 0 | 0 | 0 | 0 | 1 | 1 | 0 | 0 | 0 | 1 | 0 | 0 | 3 |
| Congo | 2 | 0 | 0 | 1 | 0 | 2 | 1 | 0 | 0 | 0 | 0 | 0 | 0 | 0 | 6 |
| Côte d'Ivoire | 0 | 0 | 0 | 0 | 1 | 0 | 1 | 0 | 0 | 0 | 0 | 0 | 0 | 0 | 2 |
| Democratic Republic of Congo | 0 | 0 | 0 | 1 | 2 | 1 | 0 | 3 | 2 | 3 | 1 | 1 | 0 | 0 | 14 |
| Equatorial Guinea | 0 | 1 | 0 | 2 | 1 | 0 | 1 | 0 | 1 | 2 | 0 | 0 | 0 | 0 | 8 |
| Eritrea | 0 | 0 | 0 | 0 | 1 | 0 | 1 | 1 | 0 | 1 | 0 | 0 | 0 | 0 | 4 |
| Ethiopia | 1 | 1 | 0 | 1 | 3 | 0 | 0 | 1 | 1 | 0 | 0 | 0 | 0 | 0 | 8 |
| Gabon | 0 | 1 | 1 | 1 | 2 | 1 | 0 | 0 | 0 | 1 | 0 | 0 | 0 | 0 | 7 |
| Gambia | 0 | 0 | 0 | 0 | 1 | 0 | 0 | 0 | 0 | 0 | 0 | 0 | 0 | 0 | 1 |
| Ghana | 2 | 1 | 3 | 1 | 3 | 1 | 2 | 1 | 4 | 0 | 0 | 0 | 1 | 0 | 19 |
| Guinea | 0 | 0 | 0 | 0 | 0 | 0 | 2 | 0 | 0 | 0 | 0 | 0 | 0 | 0 | 2 |
| Guinea-Bissau | 0 | 0 | 0 | 0 | 1 | 1 | 2 | 0 | 1 | 0 | 0 | 0 | 0 | 0 | 5 |
| Kenya | 2 | 1 | 3 | 4 | 1 | 1 | 2 | 3 | 1 | 2 | 0 | 1 | 0 | 0 | 21 |
| Liberia | 0 | 0 | 0 | 0 | 0 | 0 | 0 | 0 | 1 | 0 | 0 | 0 | 0 | 0 | 1 |
| Madagascar | 0 | 0 | 0 | 0 | 1 | 0 | 0 | 0 | 0 | 0 | 0 | 0 | 0 | 0 | 1 |
| Malawi | 0 | 0 | 1 | 0 | 1 | 0 | 0 | 0 | 0 | 0 | 0 | 0 | 0 | 0 | 2 |
| Mali | 0 | 1 | 0 | 0 | 1 | 1 | 2 | 0 | 0 | 0 | 0 | 0 | 0 | 0 | 5 |
| Mozambique | 0 | 0 | 0 | 0 | 0 | 4 | 0 | 1 | 2 | 0 | 0 | 1 | 0 | 0 | 8 |
| Niger | 0 | 0 | 0 | 0 | 0 | 0 | 0 | 1 | 0 | 0 | 0 | 0 | 0 | 0 | 1 |
| Nigeria | 0 | 0 | 0 | 1 | 2 | 1 | 2 | 0 | 3 | 0 | 0 | 0 | 0 | 0 | 9 |
| Rwanda | 2 | 0 | 0 | 0 | 1 | 1 | 0 | 0 | 1 | 2 | 0 | 1 | 0 | 1 | 9 |
| Senegal | 0 | 0 | 0 | 0 | 1 | 1 | 3 | 3 | 1 | 1 | 0 | 0 | 0 | 0 | 10 |
| Sierra Leone | 0 | 0 | 0 | 0 | 0 | 0 | 1 | 0 | 1 | 0 | 0 | 0 | 0 | 0 | 2 |
| Somalia | 0 | 0 | 0 | 0 | 0 | 0 | 0 | 1 | 0 | 0 | 0 | 0 | 0 | 0 | 1 |
| South Africa | 1 | 1 | 1 | 0 | 0 | 0 | 0 | 0 | 1 | 0 | 0 | 0 | 0 | 0 | 4 |
| South Sudan | 0 | 0 | 0 | 0 | 0 | 0 | 0 | 0 | 0 | 1 | 0 | 0 | 0 | 0 | 1 |
| Sudan | 0 | 0 | 0 | 0 | 0 | 0 | 2 | 2 | 1 | 0 | 1 | 0 | 0 | 0 | 6 |
| Togo | 0 | 0 | 0 | 1 | 0 | 0 | 0 | 0 | 0 | 0 | 0 | 0 | 0 | 0 | 1 |
| Uganda | 0 | 0 | 2 | 2 | 3 | 2 | 4 | 3 | 3 | 4 | 1 | 2 | 1 | 0 | 27 |
| Tanzania | 0 | 1 | 1 | 0 | 1 | 1 | 1 | 1 | 1 | 1 | 0 | 0 | 0 | 0 | 8 |
| Zambia | 0 | 0 | 0 | 0 | 0 | 1 | 1 | 1 | 0 | 0 | 0 | 0 | 0 | 0 | 3 |
| Zanzibar | 0 | 0 | 0 | 0 | 0 | 0 | 0 | 1 | 0 | 0 | 0 | 0 | 0 | 0 | 1 |

- **Table 5**: Studies on Sulfadoxine Pyrimethamine (SP) resistance (*Pfdhfr / Pfdhps)* included in the meta-analysis per year and per country

| **Country** | **2010** | **2011** | **2012** | **2013** | **2014** | **2015** | **2016** | **2017** | **2018** | **2019** | **2020** | **2021** | **2022** | **2023** | **Total** |
| --- | --- | --- | --- | --- | --- | --- | --- | --- | --- | --- | --- | --- | --- | --- | --- |
| Angola | 0 | 2 | 0 | 0 | 0 | 0 | 0 | 1 | 1 | 1 | 0 | 0 | 0 | 0 | 5 |
| Benin | 0 | 1 | 1 | 0 | 0 | 0 | 0 | 1 | 0 | 1 | 0 | 0 | 0 | 0 | 4 |
| Burkina Faso | 2 | 0 | 2 | 0 | 0 | 3 | 1 | 0 | 1 | 0 | 1 | 1 | 1 | 0 | 12 |
| Cameroon | 0 | 1 | 0 | 0 | 2 | 1 | 2 | 3 | 1 | 1 | 3 | 1 | 0 | 0 | 15 |
| Cape Verde | 0 | 0 | 0 | 0 | 0 | 0 | 0 | 1 | 0 | 0 | 0 | 0 | 0 | 0 | 1 |
| Central African Republic | 0 | 0 | 0 | 0 | 0 | 0 | 0 | 0 | 1 | 0 | 0 | 0 | 0 | 0 | 1 |
| Chad | 0 | 0 | 0 | 0 | 0 | 0 | 2 | 1 | 1 | 0 | 0 | 1 | 0 | 0 | 5 |
| Comoros | 0 | 0 | 0 | 1 | 0 | 0 | 0 | 0 | 0 | 0 | 0 | 0 | 0 | 0 | 1 |
| Congo | 1 | 0 | 0 | 2 | 0 | 1 | 0 | 1 | 0 | 0 | 0 | 0 | 0 | 0 | 5 |
| Democratic Republic of the Congo | 0 | 0 | 0 | 1 | 3 | 1 | 0 | 1 | 2 | 1 | 2 | 2 | 0 | 0 | 13 |
| Equatorial Guinea | 0 | 1 | 1 | 2 | 2 | 0 | 1 | 1 | 0 | 1 | 0 | 0 | 0 | 0 | 9 |
| Ethiopia | 0 | 0 | 0 | 0 | 1 | 0 | 0 | 0 | 1 | 0 | 0 | 0 | 0 | 0 | 2 |
| Gabon | 0 | 2 | 1 | 1 | 1 | 0 | 3 | 1 | 0 | 1 | 0 | 0 | 0 | 0 | 10 |
| Gambia | 0 | 0 | 0 | 0 | 0 | 0 | 1 | 0 | 1 | 0 | 0 | 0 | 0 | 0 | 2 |
| Ghana | 2 | 1 | 3 | 1 | 3 | 1 | 2 | 3 | 3 | 1 | 0 | 0 | 1 | 0 | 21 |
| Guinea | 0 | 0 | 0 | 0 | 0 | 0 | 1 | 0 | 1 | 0 | 0 | 0 | 0 | 0 | 2 |
| Guinea-Bissau | 0 | 0 | 0 | 0 | 1 | 1 | 2 | 0 | 1 | 0 | 0 | 0 | 0 | 0 | 5 |
| Kenya | 1 | 1 | 2 | 4 | 0 | 2 | 2 | 1 | 2 | 1 | 0 | 0 | 0 | 0 | 16 |
| Liberia | 0 | 0 | 0 | 0 | 0 | 0 | 0 | 0 | 1 | 0 | 0 | 0 | 0 | 0 | 1 |
| Madagascar | 0 | 0 | 0 | 0 | 0 | 0 | 0 | 0 | 1 | 0 | 0 | 1 | 0 | 0 | 2 |
| Malawi | 0 | 2 | 2 | 0 | 0 | 0 | 0 | 0 | 0 | 0 | 0 | 0 | 0 | 0 | 4 |
| Mali | 0 | 0 | 1 | 0 | 3 | 1 | 3 | 1 | 1 | 0 | 1 | 0 | 0 | 0 | 11 |
| Mauritania | 1 | 0 | 0 | 0 | 0 | 0 | 0 | 0 | 0 | 0 | 0 | 0 | 0 | 0 | 1 |
| Mozambique | 1 | 0 | 0 | 0 | 0 | 1 | 0 | 0 | 1 | 0 | 0 | 1 | 0 | 0 | 4 |
| Niger | 0 | 0 | 1 | 0 | 0 | 0 | 1 | 2 | 1 | 0 | 0 | 0 | 0 | 0 | 5 |
| Nigeria | 1 | 1 | 0 | 0 | 2 | 3 | 3 | 1 | 3 | 1 | 0 | 3 | 0 | 0 | 18 |
| Rwanda | 1 | 0 | 0 | 0 | 0 | 1 | 0 | 0 | 1 | 0 | 0 | 1 | 0 | 0 | 4 |
| Senegal | 3 | 2 | 0 | 1 | 2 | 0 | 0 | 0 | 0 | 0 | 0 | 0 | 0 | 0 | 8 |
| Sierra Leone | 0 | 0 | 0 | 0 | 0 | 0 | 1 | 0 | 1 | 0 | 0 | 0 | 0 | 0 | 2 |
| Somalia | 0 | 1 | 0 | 0 | 0 | 1 | 0 | 0 | 1 | 0 | 0 | 0 | 0 | 0 | 3 |
| South Africa | 1 | 1 | 1 | 0 | 0 | 0 | 0 | 0 | 1 | 0 | 0 | 0 | 0 | 0 | 4 |
| South Sudan | 0 | 0 | 0 | 0 | 0 | 0 | 0 | 0 | 0 | 1 | 0 | 0 | 0 | 0 | 1 |
| Sudan | 0 | 0 | 1 | 0 | 0 | 0 | 1 | 1 | 1 | 0 | 0 | 0 | 0 | 0 | 4 |
| Uganda | 2 | 2 | 4 | 2 | 1 | 1 | 1 | 2 | 1 | 1 | 0 | 0 | 0 | 0 | 17 |
| United Republic of Tanzania | 0 | 3 | 0 | 1 | 0 | 1 | 0 | 1 | 0 | 1 | 0 | 0 | 0 | 0 | 7 |
| Zambia | 0 | 1 | 0 | 1 | 1 | 1 | 0 | 1 | 0 | 0 | 0 | 0 | 0 | 0 | 5 |

# Table 6: Descriptive analysis of the methods used for the detection of mutations among the studies included in the meta-analysis

| Variable | Category | n | % |
| --- | --- | --- | --- |
| Sequencing (N=352) | Yes | 243 | 69.0 |
|  | No | 109 | 31.0 |
| Sequencing technologies (N=243) | Sanger | 176 | 72.4 |
|  | NGS | 64 | 21.8 |
|  | Nanopore | 2 | 0.8 |
|  | Sanger and NGS | 1 | 0.4 |
| Non-Sequencing (N=142) | RFLP | 78 | 54.9 |
|  | HRM | 17 | 12.0 |
|  | qPCR | 17 | 12.0 |
|  | LDR | 9 | 6.3 |
|  | qPCR and RFLP | 4 | 2.8 |
|  | SSOP | 6 | 4.2 |
|  | qPCR and HRM | 2 | 1.4 |
|  | qPCR and LDR | 2 | 1.4 |
|  | RFLP and SSOP | 2 | 1.4 |
|  | ASPE | 1 | 0.7 |
|  | PCR-Microsatellites | 1 | 0.7 |
|  | PET | 1 | 0.7 |
|  | qPCR and SSOP | 1 | 0.7 |
|  | RFLP and HRM | 1 | 0.7 |

*RFLP: Restriction Fragment Length Polymorphism; HRM : High Resolution Melting; qPCR : quantitative Polymerase Chain Reaction; LDR: Ligase Detection Reaction; SSOP : Sequence Specific Oligonucleotide Probe; ASPE : Allelic Specific Primer Extension (ASPE)

## Table 7: Temporal trends of the use of sequencing for the detection of mutations among the studies published from 2011 to 2024

| Year of publication | N_year | No | Yes |
| --- | --- | --- | --- |
| 2011 | 2 | 2 (100.0%) | 0 (0.0%) |
| 2012 | 5 | 5 (100.0%) | 0 (0.0%) |
| 2013 | 12 | 8 (66.7%) | 4 (33.3%) |
| 2014 | 16 | 10 (62.5%) | 6 (37.5%) |
| 2015 | 28 | 12 (42.9%) | 16 (57.1%) |
| 2016 | 25 | 7 (28.0%) | 18 (72.0%) |
| 2017 | 25 | 10 (40.0%) | 15 (60.0%) |
| 2018 | 27 | 5 (18.5%) | 22 (81.5%) |
| 2019 | 30 | 9 (30.0%) | 21 (70.0%) |
| 2020 | 43 | 5 (11.6%) | 38 (88.4%) |
| 2021 | 42 | 14 (33.3%) | 28 (66.7%) |
| 2022 | 21 | 7 (33.3%) | 14 (66.7%) |
| 2023 | 40 | 8 (20.0%) | 32 (80.0%) |
| 2024 | 36 | 7 (19.4%) | 29 (80.6%) |

## Table 8: Temporal trends of the type of sequencing methods used for the detection of mutations among the studies published from 2013 to 2024

| Year of publication | N_year | Sanger | NGS | Nanopore |
| --- | --- | --- | --- | --- |
| 2013 | 4 | 4 (100.0%) | 0 (0.0%) | 0 (0.0%) |
| 2014 | 6 | 5 (83.3%) | 1 (16.7%) | 0 (0.0%) |
| 2015 | 16 | 14 (87.5%) | 2 (12.5%) | 0 (0.0%) |
| 2016 | 18 | 16 (88.9%) | 2 (11.1%) | 0 (0.0%) |
| 2017 | 15 | 12 (80.0%) | 3 (20.0%) | 0 (0.0%) |
| 2018 | 22 | 20 (90.9%) | 2 (9.1%) | 0 (0.0%) |
| 2019 | 21 | 16 (76.2%) | 5 (23.8%) | 0 (0.0%) |
| 2020 | 38 | 30 (78.9%) | 8 (21.1%) | 0 (0.0%) |
| 2021 | 28 | 23 (82.1%) | 5 (17.9%) | 0 (0.0%) |
| 2022 | 14 | 9 (64.3%) | 5 (35.7%) | 0 (0.0%) |
| 2023 | 32 | 13 (40.6%) | 18 (56.2%) | 1 (3.1%) |
| 2024 | 29 | 14 (48.3%) | 14 (48.3%) | 1 (3.4%) |
