# Supplementary material for: Spatio-temporal trends of the prevalence of Plasmodium falciparum antimalarial drug resistance markers across sub-Saharan Africa from 2010 to 2024: a systematic review and meta-analysis
Source: BMC Infect Dis. 2026 Sep 3;26:1605. doi: 10.1186/s12879-026-14233-2 (PMC13545778; doi:10.1186/s12879-026-14233-2)
Supplement: Supplementary file 1 [file 12879_2026_14233_MOESM1_ESM.zip › Supplementary materials_15.07.2026/Supplementary_04_PfKelch 13_Forest plot by year.docx]

**Supplementary document 04**

Contents

[1.	PfKelch13 A675V	5](#_Toc219841407)

[1.1.	Forest Plot PfKelch13 A675V– Year 2010	5](#_Toc219841408)

[1.2.	Forest Plot PfKelch13 A675V– Year 2011	5](#_Toc219841409)

[1.3.	Forest Plot PfKelch13 A675V– Year 2012	6](#_Toc219841410)

[1.4.	Forest Plot PfKelch13 A675V– Year 2013	6](#_Toc219841411)

[1.5.	Forest Plot PfKelch13 A675V– Year 2014	7](#_Toc219841412)

[1.6.	Forest Plot PfKelch13 A675V– Year 2015	8](#_Toc219841413)

[1.7.	Forest Plot PfKelch13 A675V – Year 2016	9](#_Toc219841414)

[1.8.	Forest Plot PfKelch13 A675V – Year 2017	10](#_Toc219841415)

[1.9.	Forest Plot PfKelch13 A675V– Year 2018	11](#_Toc219841416)

[1.10.	Forest Plot PfKelch13 A675V– Year 2019	12](#_Toc219841417)

[1.11.	Forest Plot PfKelch13 A675V– Year 2020	12](#_Toc219841418)

[1.12.	Forest Plot PfKelch13 A675V– Year 2021	13](#_Toc219841419)

[1.13.	Forest Plot PfKelch13 A675V– Year 2022	13](#_Toc219841420)

[1.14.	Forest Plot PfKelch13 A675V– Year 2023	14](#_Toc219841421)

[2.	PfKelch13 C469Y	14](#_Toc219841422)

[2.1.	Forest Plot PfKelch13 C469Y– Year 2010	14](#_Toc219841423)

[2.2.	Forest Plot PfKelch13 C469Y– Year 2011	15](#_Toc219841424)

[2.3.	Forest Plot PfKelch13 C469Y– Year 2012	15](#_Toc219841425)

[2.4.	Forest Plot PfKelch13 C469Y– Year 2013	16](#_Toc219841426)

[2.5.	Forest Plot PfKelch13 C469Y– Year 2014	16](#_Toc219841427)

[2.6.	Forest Plot PfKelch13 C469Y– Year 2015	17](#_Toc219841428)

[2.7.	Forest Plot PfKelch13 C469Y– Year 2016	18](#_Toc219841429)

[2.8.	Forest Plot PfKelch13 C469Y– Year 2017	19](#_Toc219841430)

[2.9.	Forest Plot PfKelch13 C469Y– Year 2018	20](#_Toc219841431)

[2.10.	Forest Plot PfKelch13 C469Y– Year 2019	21](#_Toc219841432)

[2.11.	Forest Plot PfKelch13 C469Y– Year 2020	21](#_Toc219841433)

[2.12.	Forest Plot PfKelch13 C469Y– Year 2021	22](#_Toc219841434)

[2.13.	Forest Plot PfKelch13 C469Y– Year 2022	22](#_Toc219841435)

[2.14.	Forest Plot PfKelch13 C469Y– Year 2023	23](#_Toc219841436)

[3.	PfKelch13 C580Y	23](#_Toc219841437)

[3.1.	Forest Plot PfKelch13 C580Y– Year 2010	23](#_Toc219841438)

[3.2.	Forest Plot PfKelch13 C580Y– Year 2011	24](#_Toc219841439)

[3.3.	Forest Plot PfKelch13 C580Y– Year 2012	24](#_Toc219841440)

[3.4.	Forest Plot PfKelch13 C580Y– Year 2013	25](#_Toc219841441)

[3.5.	Forest Plot PfKelch13 C580Y– Year 2014	25](#_Toc219841442)

[3.6.	Forest Plot PfKelch13 C580Y– Year 2015	26](#_Toc219841443)

[3.7.	Forest Plot PfKelch13 C580Y– Year 2016	27](#_Toc219841444)

[3.8.	Forest Plot PfKelch13 C580Y– Year 2017	28](#_Toc219841445)

[3.9.	Forest Plot PfKelch13 C580Y– Year 2018	29](#_Toc219841446)

[3.10.	Forest Plot PfKelch13 C580Y– Year 2019	30](#_Toc219841447)

[3.11.	Forest Plot PfKelch13 C580Y– Year 2020	30](#_Toc219841448)

[3.12.	Forest Plot PfKelch13 C580Y– Year 2021	31](#_Toc219841449)

[3.13.	Forest Plot PfKelch13 C580Y– Year 2022	31](#_Toc219841450)

[3.14.	Forest Plot PfKelch13 C580Y– Year 2023	32](#_Toc219841451)

[4.	PfKelch13 P574L	32](#_Toc219841452)

[4.1.	Forest Plot PfKelch13 P574L– Year 2010	32](#_Toc219841453)

[4.2.	Forest Plot PfKelch13 P574L– Year 2011	33](#_Toc219841454)

[4.3.	Forest Plot PfKelch13 P574L– Year 2012	33](#_Toc219841455)

[4.4.	Forest Plot PfKelch13 P574L– Year 2013	34](#_Toc219841456)

[4.5.	Forest Plot PfKelch13 P574L– Year 2014	34](#_Toc219841457)

[4.6.	Forest Plot PfKelch13 P574L– Year 2015	35](#_Toc219841458)

[4.7.	Forest Plot PfKelch13 P574L– Year 2016	36](#_Toc219841459)

[4.8.	Forest Plot PfKelch13 P574L– Year 2017	37](#_Toc219841460)

[4.9.	Forest Plot PfKelch13 P574L– Year 2018	38](#_Toc219841461)

[4.10.	Forest Plot PfKelch13 P574L– Year 2019	39](#_Toc219841462)

[4.11.	Forest Plot PfKelch13 P574L– Year 2020	39](#_Toc219841463)

[4.12.	Forest Plot PfKelch13 P574L– Year 2021	40](#_Toc219841464)

[4.13.	Forest Plot PfKelch13 P574L– Year 2022	40](#_Toc219841465)

[4.14.	Forest Plot PfKelch13 P574L– Year 2023	41](#_Toc219841466)

[5.	PfKelch13 R539T	41](#_Toc219841467)

[5.1.	Forest Plot PfKelch13 R539T– Year 2010	41](#_Toc219841468)

[5.2.	Forest Plot PfKelch13 R539T– Year 2011	42](#_Toc219841469)

[5.3.	Forest Plot PfKelch13 R539T– Year 2012	42](#_Toc219841470)

[5.4.	Forest Plot PfKelch13 R539T– Year 2013	43](#_Toc219841471)

[5.5.	Forest Plot PfKelch13 R539T– Year 2014	43](#_Toc219841472)

[5.6.	Forest Plot PfKelch13 R539T– Year 2015	44](#_Toc219841473)

[5.7.	Forest Plot PfKelch13 R539T– Year 2016	45](#_Toc219841474)

[5.8.	Forest Plot PfKelch13 R539T– Year 2017	46](#_Toc219841475)

[5.9.	Forest Plot PfKelch13 R539T– Year 2018	47](#_Toc219841476)

[5.10.	Forest Plot PfKelch13 R539T– Year 2019	48](#_Toc219841477)

[5.11.	Forest Plot PfKelch13 R539T– Year 2020	48](#_Toc219841478)

[5.12.	Forest Plot PfKelch13 R539T– Year 2021	49](#_Toc219841479)

[5.13.	Forest Plot PfKelch13 R539T– Year 2022	49](#_Toc219841480)

[5.14.	Forest Plot PfKelch13 R539T– Year 2023	50](#_Toc219841481)

[6.	PfKelch13 R561H	50](#_Toc219841482)

[6.1.	Forest Plot PfKelch13 R561H– Year 2010	50](#_Toc219841483)

[6.2.	Forest Plot PfKelch13 R561H– Year 2011	51](#_Toc219841484)

[6.3.	Forest Plot PfKelch13 R561H– Year 2012	51](#_Toc219841485)

[6.4.	Forest Plot PfKelch13 R561H– Year 2013	52](#_Toc219841486)

[6.5.	Forest Plot PfKelch13 R561H– Year 2014	52](#_Toc219841487)

[6.6.	Forest Plot PfKelch13 R561H– Year 2015	53](#_Toc219841488)

[6.7.	Forest Plot PfKelch13 R561H– Year 2016	54](#_Toc219841489)

[6.8.	Forest Plot PfKelch13 R561H– Year 2017	55](#_Toc219841490)

[6.9.	Forest Plot PfKelch13 R561H– Year 2018	56](#_Toc219841491)

[6.10.	Forest Plot PfKelch13 R561H– Year 2019	57](#_Toc219841492)

[6.11.	Forest Plot PfKelch13 R561H– Year 2020	57](#_Toc219841493)

[6.12.	Forest Plot PfKelch13 R561H– Year 2021	58](#_Toc219841494)

[6.13.	Forest Plot PfKelch13 R561H– Year 2022	58](#_Toc219841495)

[6.14.	Forest Plot PfKelch13 R561H– Year 2023	59](#_Toc219841496)

[7.	PfKelch13 R622I	59](#_Toc219841497)

[7.1.	Forest Plot PfKelch13 R622I– Year 2010	59](#_Toc219841498)

[7.2.	Forest Plot PfKelch13 R622I– Year 2011	60](#_Toc219841499)

[7.3.	Forest Plot PfKelch13 R622I– Year 2012	60](#_Toc219841500)

[7.4.	Forest Plot PfKelch13 R622I– Year 2013	61](#_Toc219841501)

[7.5.	Forest Plot PfKelch13 R622I– Year 2014	61](#_Toc219841502)

[7.6.	Forest Plot PfKelch13 R622I– Year 2015	62](#_Toc219841503)

[7.7.	Forest Plot PfKelch13 R622I– Year 2016	63](#_Toc219841504)

[7.8.	Forest Plot PfKelch13 R622I– Year 2017	64](#_Toc219841505)

[7.9.	Forest Plot PfKelch13 R622I– Year 2018	65](#_Toc219841506)

[7.10.	Forest Plot PfKelch13 R622I– Year 2019	66](#_Toc219841507)

[7.11.	Forest Plot PfKelch13 R622I– Year 2020	66](#_Toc219841508)

[7.12.	Forest Plot PfKelch13 R622I– Year 2021	67](#_Toc219841509)

[7.13.	Forest Plot PfKelch13 R622I– Year 2022	67](#_Toc219841510)

[7.14.	Forest Plot PfKelch13 R622I– Year 2023	68](#_Toc219841511)

[8.	PfKelch13 G449A	68](#_Toc219841512)

[8.1.	Forest Plot PfKelch13 G449A– Year 2010	68](#_Toc219841513)

[8.2.	Forest Plot PfKelch13 G449A– Year 2011	69](#_Toc219841514)

[8.3.	Forest Plot PfKelch13 G449A– Year 2012	69](#_Toc219841515)

[8.4.	Forest Plot PfKelch13 G449A– Year 2013	70](#_Toc219841516)

[8.5.	Forest Plot PfKelch13 G449A– Year 2014	70](#_Toc219841517)

[8.6.	Forest Plot PfKelch13 G449A– Year 2015	71](#_Toc219841518)

[8.7.	Forest Plot PfKelch13 G449A– Year 2016	72](#_Toc219841519)

[8.8.	Forest Plot PfKelch13 G449A– Year 2017	73](#_Toc219841520)

[8.9.	Forest Plot PfKelch13 G449A– Year 2018	74](#_Toc219841521)

[8.10.	Forest Plot PfKelch13 G449A– Year 2019	75](#_Toc219841522)

[8.11.	Forest Plot PfKelch13 G449A– Year 2020	75](#_Toc219841523)

[8.12.	Forest Plot PfKelch13 G449A– Year 2021	76](#_Toc219841524)

[8.13.	Forest Plot PfKelch13 G449A– Year 2022	76](#_Toc219841525)

[8.14.	Forest Plot PfKelch13 G449A– Year 2023	77](#_Toc219841526)

[9.	PfKelch13 P441L	77](#_Toc219841527)

[9.1.	Forest Plot PfKelch13 P441L– Year 2010	77](#_Toc219841528)

[9.2.	Forest Plot PfKelch13 P441L– Year 2011	78](#_Toc219841529)

[9.3.	Forest Plot PfKelch13 P441L– Year 2012	78](#_Toc219841530)

[9.4.	Forest Plot PfKelch13 P441L– Year 2013	79](#_Toc219841531)

[9.5.	Forest Plot PfKelch13 P441L– Year 2014	79](#_Toc219841532)

[9.6.	Forest Plot PfKelch13 P441L– Year 2015	80](#_Toc219841533)

[9.7.	Forest Plot PfKelch13 P441L– Year 2016	81](#_Toc219841534)

[9.8.	Forest Plot PfKelch13 P441L– Year 2017	82](#_Toc219841535)

[9.9.	Forest Plot PfKelch13 P441L– Year 2018	83](#_Toc219841536)

[9.10.	Forest Plot PfKelch13 P441L– Year 2019	84](#_Toc219841537)

[9.11.	Forest Plot PfKelch13 P441L– Year 2020	84](#_Toc219841538)

[9.12.	Forest Plot PfKelch13 P441L– Year 2021	85](#_Toc219841539)

[9.13.	Forest Plot PfKelch13 P441L– Year 2022	85](#_Toc219841540)

[9.14.	Forest Plot PfKelch13 P441L– Year 2023	86](#_Toc219841541)

[10.	PfKelch13 P527H	86](#_Toc219841542)

[10.1.	Forest Plot PfKelch13 P527H– Year 2010	86](#_Toc219841543)

[10.2.	Forest Plot PfKelch13 P527H– Year 2011	87](#_Toc219841544)

[10.3.	Forest Plot PfKelch13 P527H– Year 2012	87](#_Toc219841545)

[10.4.	Forest Plot PfKelch13 P527H– Year 2013	88](#_Toc219841546)

[10.5.	Forest Plot PfKelch13 P527H– Year 2014	88](#_Toc219841547)

[10.6.	Forest Plot PfKelch13 P527H– Year 2015	89](#_Toc219841548)

[10.7.	Forest Plot PfKelch13 P527H– Year 2016	90](#_Toc219841549)

[10.8.	Forest Plot PfKelch13 P527H– Year 2017	91](#_Toc219841550)

[10.9.	Forest Plot PfKelch13 P527H– Year 2018	92](#_Toc219841551)

[10.10.	Forest Plot PfKelch13 P527H– Year 2019	93](#_Toc219841552)

[10.11.	Forest Plot PfKelch13 P527H– Year 2020	93](#_Toc219841553)

[10.12.	Forest Plot PfKelch13 P527H– Year 2021	94](#_Toc219841554)

[10.13.	Forest Plot PfKelch13 P527H– Year 2022	94](#_Toc219841555)

[10.14.	Forest Plot PfKelch13 P527H– Year 2023	95](#_Toc219841556)

# 1. PfKelch13 A675V

## 1.1. Forest Plot PfKelch13 A675V– Year 2010

## 1.2. Forest Plot PfKelch13 A675V– Year 2011

## 1.3. Forest Plot PfKelch13 A675V– Year 2012

## 1.4. Forest Plot PfKelch13 A675V– Year 2013

## 1.5. Forest Plot PfKelch13 A675V– Year 2014

## 1.6. Forest Plot PfKelch13 A675V– Year 2015

## 1.7. Forest Plot PfKelch13 A675V – Year 2016

## 1.8. Forest Plot PfKelch13 A675V – Year 2017

## 1.9. Forest Plot PfKelch13 A675V– Year 2018

## 1.10. Forest Plot PfKelch13 A675V– Year 2019

## 1.11. Forest Plot PfKelch13 A675V– Year 2020

## 1.12. Forest Plot PfKelch13 A675V– Year 2021

## 1.13. Forest Plot PfKelch13 A675V– Year 2022

## 1.14. Forest Plot PfKelch13 A675V– Year 2023

# 2. PfKelch13 C469Y

## 2.1. Forest Plot PfKelch13 C469Y– Year 2010

## 2.2. Forest Plot PfKelch13 C469Y– Year 2011

## 2.3. Forest Plot PfKelch13 C469Y– Year 2012

## 2.4. Forest Plot PfKelch13 C469Y– Year 2013

## 2.5. Forest Plot PfKelch13 C469Y– Year 2014

## 2.6. Forest Plot PfKelch13 C469Y– Year 2015

## 2.7. Forest Plot PfKelch13 C469Y– Year 2016

## 2.8. Forest Plot PfKelch13 C469Y– Year 2017

## 2.9. Forest Plot PfKelch13 C469Y– Year 2018

## 2.10. Forest Plot PfKelch13 C469Y– Year 2019

## 2.11. Forest Plot PfKelch13 C469Y– Year 2020

## 2.12. Forest Plot PfKelch13 C469Y– Year 2021

## 2.13. Forest Plot PfKelch13 C469Y– Year 2022

## 2.14. Forest Plot PfKelch13 C469Y– Year 2023

# 3. PfKelch13 C580Y

## 3.1. Forest Plot PfKelch13 C580Y– Year 2010

## 3.2. Forest Plot PfKelch13 C580Y– Year 2011

## 3.3. Forest Plot PfKelch13 C580Y– Year 2012

## 3.4. Forest Plot PfKelch13 C580Y– Year 2013

## 3.5. Forest Plot PfKelch13 C580Y– Year 2014

## 3.6. Forest Plot PfKelch13 C580Y– Year 2015

## 3.7. Forest Plot PfKelch13 C580Y– Year 2016

## 3.8. Forest Plot PfKelch13 C580Y– Year 2017

## 3.9. Forest Plot PfKelch13 C580Y– Year 2018

## 3.10. Forest Plot PfKelch13 C580Y– Year 2019

## 3.11. Forest Plot PfKelch13 C580Y– Year 2020

## 3.12. Forest Plot PfKelch13 C580Y– Year 2021

## 3.13. Forest Plot PfKelch13 C580Y– Year 2022

## 3.14. Forest Plot PfKelch13 C580Y– Year 2023

# 4. PfKelch13 P574L

## 4.1. Forest Plot PfKelch13 P574L– Year 2010

## 4.2. Forest Plot PfKelch13 P574L– Year 2011

## 4.3. Forest Plot PfKelch13 P574L– Year 2012

## 4.4. Forest Plot PfKelch13 P574L– Year 2013

## 4.5. Forest Plot PfKelch13 P574L– Year 2014

## 4.6. Forest Plot PfKelch13 P574L– Year 2015

## 4.7. Forest Plot PfKelch13 P574L– Year 2016

## 4.8. Forest Plot PfKelch13 P574L– Year 2017

## 4.9. Forest Plot PfKelch13 P574L– Year 2018

## 4.10. Forest Plot PfKelch13 P574L– Year 2019

## 4.11. Forest Plot PfKelch13 P574L– Year 2020

## 4.12. Forest Plot PfKelch13 P574L– Year 2021

## 4.13. Forest Plot PfKelch13 P574L– Year 2022

## 4.14. Forest Plot PfKelch13 P574L– Year 2023

# 5. PfKelch13 R539T

## 5.1. Forest Plot PfKelch13 R539T– Year 2010

## 5.2. Forest Plot PfKelch13 R539T– Year 2011

## 5.3. Forest Plot PfKelch13 R539T– Year 2012

## 5.4. Forest Plot PfKelch13 R539T– Year 2013

## 5.5. Forest Plot PfKelch13 R539T– Year 2014

## 5.6. Forest Plot PfKelch13 R539T– Year 2015

## 5.7. Forest Plot PfKelch13 R539T– Year 2016

## 5.8. Forest Plot PfKelch13 R539T– Year 2017

## 5.9. Forest Plot PfKelch13 R539T– Year 2018

## 5.10. Forest Plot PfKelch13 R539T– Year 2019

## 5.11. Forest Plot PfKelch13 R539T– Year 2020

## 5.12. Forest Plot PfKelch13 R539T– Year 2021

## 5.13. Forest Plot PfKelch13 R539T– Year 2022

## 5.14. Forest Plot PfKelch13 R539T– Year 2023

# 6. PfKelch13 R561H

## 6.1. Forest Plot PfKelch13 R561H– Year 2010

## 6.2. Forest Plot PfKelch13 R561H– Year 2011

## 6.3. Forest Plot PfKelch13 R561H– Year 2012

## 6.4. Forest Plot PfKelch13 R561H– Year 2013

## 6.5. Forest Plot PfKelch13 R561H– Year 2014

## 6.6. Forest Plot PfKelch13 R561H– Year 2015

## 6.7. Forest Plot PfKelch13 R561H– Year 2016

## 6.8. Forest Plot PfKelch13 R561H– Year 2017

## 6.9. Forest Plot PfKelch13 R561H– Year 2018

## 6.10. Forest Plot PfKelch13 R561H– Year 2019

## 6.11. Forest Plot PfKelch13 R561H– Year 2020

## 6.12. Forest Plot PfKelch13 R561H– Year 2021

## 6.13. Forest Plot PfKelch13 R561H– Year 2022

## 6.14. Forest Plot PfKelch13 R561H– Year 2023

# 7. PfKelch13 R622I

## 7.1. Forest Plot PfKelch13 R622I– Year 2010

## 7.2. Forest Plot PfKelch13 R622I– Year 2011

## 7.3. Forest Plot PfKelch13 R622I– Year 2012

## 7.4. Forest Plot PfKelch13 R622I– Year 2013

## 7.5. Forest Plot PfKelch13 R622I– Year 2014

## 7.6. Forest Plot PfKelch13 R622I– Year 2015

## 7.7. Forest Plot PfKelch13 R622I– Year 2016

## 7.8. Forest Plot PfKelch13 R622I– Year 2017

## 7.9. Forest Plot PfKelch13 R622I– Year 2018

## 7.10. Forest Plot PfKelch13 R622I– Year 2019

## 7.11. Forest Plot PfKelch13 R622I– Year 2020

## 7.12. Forest Plot PfKelch13 R622I– Year 2021

## 7.13. Forest Plot PfKelch13 R622I– Year 2022

## 7.14. Forest Plot PfKelch13 R622I– Year 2023

# 8. PfKelch13 G449A

## 8.1. Forest Plot PfKelch13 G449A– Year 2010

## 8.2. Forest Plot PfKelch13 G449A– Year 2011

## 8.3. Forest Plot PfKelch13 G449A– Year 2012

## 8.4. Forest Plot PfKelch13 G449A– Year 2013

## 8.5. Forest Plot PfKelch13 G449A– Year 2014

## 8.6. Forest Plot PfKelch13 G449A– Year 2015

## 8.7. Forest Plot PfKelch13 G449A– Year 2016

## 8.8. Forest Plot PfKelch13 G449A– Year 2017

## 8.9. Forest Plot PfKelch13 G449A– Year 2018

## 8.10. Forest Plot PfKelch13 G449A– Year 2019

## 8.11. Forest Plot PfKelch13 G449A– Year 2020

## 8.12. Forest Plot PfKelch13 G449A– Year 2021

## 8.13. Forest Plot PfKelch13 G449A– Year 2022

## 8.14. Forest Plot PfKelch13 G449A– Year 2023

# 9. PfKelch13 P441L

## 9.1. Forest Plot PfKelch13 P441L– Year 2010

## 9.2. Forest Plot PfKelch13 P441L– Year 2011

## 9.3. Forest Plot PfKelch13 P441L– Year 2012

## 9.4. Forest Plot PfKelch13 P441L– Year 2013

## 9.5. Forest Plot PfKelch13 P441L– Year 2014

## 9.6. Forest Plot PfKelch13 P441L– Year 2015

## 9.7. Forest Plot PfKelch13 P441L– Year 2016

## 9.8. Forest Plot PfKelch13 P441L– Year 2017

## 9.9. Forest Plot PfKelch13 P441L– Year 2018

## 9.10. Forest Plot PfKelch13 P441L– Year 2019

## 9.11. Forest Plot PfKelch13 P441L– Year 2020

## 9.12. Forest Plot PfKelch13 P441L– Year 2021

## 9.13. Forest Plot PfKelch13 P441L– Year 2022

## 9.14. Forest Plot PfKelch13 P441L– Year 2023

# 10. PfKelch13 P527H

## 10.1. Forest Plot PfKelch13 P527H– Year 2010

## 10.2. Forest Plot PfKelch13 P527H– Year 2011

## 10.3. Forest Plot PfKelch13 P527H– Year 2012

## 10.4. Forest Plot PfKelch13 P527H– Year 2013

## 10.5. Forest Plot PfKelch13 P527H– Year 2014

## 10.6. Forest Plot PfKelch13 P527H– Year 2015

## 10.7. Forest Plot PfKelch13 P527H– Year 2016

## 10.8. Forest Plot PfKelch13 P527H– Year 2017

## 10.9. Forest Plot PfKelch13 P527H– Year 2018

## 10.10. Forest Plot PfKelch13 P527H– Year 2019

## 10.11. Forest Plot PfKelch13 P527H– Year 2020

## 10.12. Forest Plot PfKelch13 P527H– Year 2021

## 10.13. Forest Plot PfKelch13 P527H– Year 2022

## 10.14. Forest Plot PfKelch13 P527H– Year 2023
