# Supplementary material for: Spatio-temporal trends of the prevalence of Plasmodium falciparum antimalarial drug resistance markers across sub-Saharan Africa from 2010 to 2024: a systematic review and meta-analysis
Source: BMC Infect Dis. 2026 Sep 3;26:1605. doi: 10.1186/s12879-026-14233-2 (PMC13545778; doi:10.1186/s12879-026-14233-2)
Supplement: Supplementary file 1 [file 12879_2026_14233_MOESM1_ESM.zip › Supplementary materials_15.07.2026/Supplementary_05_ Additional map of PfKelch13_Pfcrt_Pfmdr1_Pfdhfr_Pfdhps.docx]

**Supplementary document 5**

1. ***Pf Kelch 13***

**Figure 1**: **Country-level temporal trends in the prevalence of candidate and associated *PfKelch13* mutations across sub-Saharan Africa from 2014 to 2023.** The figure shows the temporal distribution of the candidate or associated *PfKelch13* mutations G449A, P441L, and P527H reported by country. Each panel represents a single mutation. Each circle corresponds to a country-year observation. Circle size is proportional to the number of samples analysed, whereas circle colour indicates the reported mutation prevalence (%) according to the colour scale. Only country-year observations in which the corresponding mutation was detected are displayed.

2. ***Pfcrt***

**Figure 2**: **Country-level temporal trends in the prevalence of the *Pfcrt* K76T mutation across sub-Saharan Africa from 2010 to 2023**. Each circle represents a country-year observation reporting the prevalence of the *Pfcrt* K76T mutation. Circle size is proportional to the number of samples analysed, while circle colour indicates the reported mutation prevalence (%) according to the colour scale. The figure illustrates the temporal evolution and regional variation in *Pfcrt* K76T prevalence across sub-Saharan Africa during the study period.

3. ***Pfmdr1***

**Figure 3**: **Country-level temporal trends in the prevalence of the *Pfmdr1* N86Y mutation across sub-Saharan Africa from 2010 to 2023**. Each circle represents a country-year observation reporting the prevalence of the *Pfmdr1* N86Y mutation. Circle size is proportional to the number of samples analysed, while circle colour indicates the reported mutation prevalence (%) according to the accompanying colour scale. The figure illustrates the temporal evolution and regional variation in *Pfmdr1* N86Y prevalence across sub-Saharan Africa during the study period.

**Figure 4**: **Country-level temporal trends in the prevalence of the *Pfmdr1* Y184F mutation across sub-Saharan Africa from 2010 to 2023**. Each circle represents a country-year observation reporting the prevalence of the *Pfmdr1* Y184F mutation. Circle size is proportional to the number of samples analysed, while circle colour indicates the reported mutation prevalence (%) according to the accompanying colour scale. The figure illustrates the temporal evolution and regional variation in *Pfmdr1* Y184F prevalence across sub-Saharan Africa during the study period, with the mutation remaining common in many countries throughout the study period.

**Figure 5:** **Country-level temporal trends in the prevalence of the *Pfmdr1* NFD (N86–184F–D1246) haplotype across sub-Saharan Africa from 2010 to 2023.** Each circle represents a country-year observation reporting the prevalence of the *Pfmdr1* NFD haplotype. Circle size is proportional to the number of samples analysed, while circle colour indicates the reported haplotype prevalence (%) according to the accompanying colour scale. The NFD haplotype comprises the wild-type N86, mutant 184F, and wild-type D1246 alleles of *Pfmdr1*. The figure illustrates the temporal evolution and regional variation in the prevalence of the NFD haplotype across sub-Saharan Africa during the study period.

4. ***Pfdhfr/Pfdhps***

**Figure 6**: **Spatial distribution and country-level temporal trends in the pooled prevalence of the *Pfdhfr* N51I mutation across sub-Saharan Africa from 2010 to 2023**. **(a)** Geographic distribution of the pooled prevalence of the *Pfdhfr* N51I mutation by country. Countries with available data are coloured according to the pooled prevalence (%) shown on the accompanying colour scale, whereas countries without eligible data are shown in grey. **(b)** Country-level temporal distribution of the *Pfdhfr* N51I mutation from 2010 to 2023. Each circle represents a country-year observation. Circle size is proportional to the number of samples analysed, and circle colour indicates the reported mutation prevalence (%) according to the colour scale. The figure demonstrates the consistently high prevalence of the *Pfdhfr* N51I mutation across much of sub-Saharan Africa throughout the study period.

**Figure 7:** **Spatial distribution and country-level temporal trends in the pooled prevalence of the *Pfdhfr* C59R mutation across sub-Saharan Africa from 2010 to 2023.** **(a)** Geographic distribution of the pooled prevalence of the *Pfdhfr* C59R mutation by country. Countries with available data are coloured according to the pooled prevalence (%) shown on the accompanying colour scale, whereas countries without eligible data are shown in grey. **(b)** Country-level temporal distribution of the *Pfdhfr* C59R mutation from 2010 to 2023. Each circle represents a country-year observation. Circle size is proportional to the number of samples analysed, and circle colour indicates the reported mutation prevalence (%) according to the colour scale. The figure illustrates the persistently high prevalence of the *Pfdhfr* C59R mutation across much of sub-Saharan Africa throughout the study period, with some geographic variability between countries.

**Figure 8:** **Spatial distribution and country-level temporal trends in the pooled prevalence of the *Pfdhfr* S108N mutation across sub-Saharan Africa from 2010 to 2023**. **(a)** Geographic distribution of the pooled prevalence of the *Pfdhfr* S108N mutation by country. Countries with available data are coloured according to the pooled prevalence (%) shown on the accompanying colour scale, whereas countries without eligible data are shown in grey. **(b)** Country-level temporal distribution of the *Pfdhfr* S108N mutation from 2010 to 2023. Each circle represents a country-year observation. Circle size is proportional to the number of samples analysed, and circle colour indicates the reported mutation prevalence (%) according to the colour scale. The figure demonstrates the near-fixation and persistently high prevalence of the *Pfdhfr* S108N mutation across most of sub-Saharan Africa throughout the study period.

**Figure 9: Spatial distribution and country-level temporal trends in the pooled prevalence of the *Pfdhps* A437G mutation across sub-Saharan Africa from 2010 to 2023.** **(a)** Geographic distribution of the pooled prevalence of the *Pfdhps* A437G mutation by country. Countries with available data are coloured according to the pooled prevalence (%) shown on the accompanying colour scale, whereas countries without eligible data are shown in grey. **(b)** Country-level temporal distribution of the *Pfdhps* A437G mutation from 2010 to 2023. Each circle represents a country-year observation. Circle size is proportional to the number of samples analysed, and circle colour indicates the reported mutation prevalence (%) according to the colour scale. The figure demonstrates the widespread distribution and persistently high prevalence of the *Pfdhps* A437G mutation across sub-Saharan Africa throughout the study period, although some geographical variation is evident.

**Figure 10: Spatial distribution and country-level temporal trends in the pooled prevalence of the *Pfdhps* S436A/F mutation across sub-Saharan Africa from 2010 to 2023.** **(a)** Geographic distribution of the pooled prevalence of the *Pfdhps* S436A/F mutation by country. Countries with available data are coloured according to the pooled prevalence (%) shown on the accompanying colour scale, whereas countries without eligible data are shown in grey. **(b)** Country-level temporal distribution of the *Pfdhps* S436A/F mutation from 2010 to 2023. Each circle represents a country-year observation. Circle size is proportional to the number of samples analysed, and circle colour indicates the reported mutation prevalence (%) according to the accompanying colour scale. The figure illustrates the heterogeneous geographic distribution of the *Pfdhps* S436A/F mutation across sub-Saharan Africa, with substantial variation in prevalence between countries and over time.
