# Supplementary material for: Spatio-temporal trends of the prevalence of Plasmodium falciparum antimalarial drug resistance markers across sub-Saharan Africa from 2010 to 2024: a systematic review and meta-analysis
Source: BMC Infect Dis. 2026 Sep 3;26:1605. doi: 10.1186/s12879-026-14233-2 (PMC13545778; doi:10.1186/s12879-026-14233-2)
Supplement: Supplementary file 1 [file 12879_2026_14233_MOESM1_ESM.zip › Supplementary materials_15.07.2026/Supplementary_06_Pfcrt _Pfmdr1_Forest plot by year.docx]

**Supplementary document_06**

Contents

[1.	Pfcrt K76T	3](#_Toc219846292)

[1.1.	Forest Plot Pfcrt K76T– Year 2010	3](#_Toc219846293)

[1.2.	Forest Plot Pfcrt K76T– Year 2011	3](#_Toc219846294)

[1.3.	Forest Plot Pfcrt K76T– Year 2012	4](#_Toc219846295)

[1.4.	Forest Plot Pfcrt K76T– Year 2013	5](#_Toc219846296)

[1.5.	Forest Plot Pfcrt K76T– Year 2014	6](#_Toc219846297)

[1.6.	Forest Plot Pfcrt K76T– Year 2015	7](#_Toc219846298)

[1.7.	Forest Plot Pfcrt K76T– Year 2016	8](#_Toc219846299)

[1.8.	Forest Plot Pfcrt K76T– Year 2017	9](#_Toc219846300)

[1.9.	Forest Plot Pfcrt K76T– Year 2018	10](#_Toc219846301)

[1.10.	Forest Plot Pfcrt K76T– Year 2019	11](#_Toc219846302)

[1.11.	Forest Plot Pfcrt K76T– Year 2020	11](#_Toc219846303)

[1.12.	Forest Plot Pfcrt K76T– Year 2021	12](#_Toc219846304)

[1.13.	Forest Plot Pfcrt K76T– Year 2022	12](#_Toc219846305)

[1.14.	Forest Plot Pfcrt K76T– Year 2023	12](#_Toc219846306)

[2.	Pfmdr1 N86Y	13](#_Toc219846307)

[2.1.	Forest Plot Pfmdr1 N86Y– Year 2010	13](#_Toc219846308)

[2.2.	Forest Plot Pfmdr1 N86Y– Year 2011	13](#_Toc219846309)

[2.3.	Forest Plot Pfmdr1 N86Y– Year 2012	14](#_Toc219846310)

[2.4.	Forest Plot Pfmdr1 N86Y– Year 2013	14](#_Toc219846311)

[2.5.	Forest Plot Pfmdr1 N86Y– Year 2014	15](#_Toc219846312)

[2.6.	Forest Plot Pfmdr1 N86Y– Year 2015	15](#_Toc219846313)

[2.7.	Forest Plot Pfmdr1 N86Y– Year 2016	16](#_Toc219846314)

[2.8.	Forest Plot Pfmdr1 N86Y– Year 2017	17](#_Toc219846315)

[2.9.	Forest Plot Pfmdr1 N86Y– Year 2018	18](#_Toc219846316)

[2.10.	Forest Plot Pfmdr1 N86Y– Year 2019	19](#_Toc219846317)

[2.11.	Forest Plot Pfmdr1 N86Y– Year 2020	19](#_Toc219846318)

[2.12.	Forest Plot Pfmdr1 N86Y– Year 2021	20](#_Toc219846319)

[2.13.	Forest Plot Pfmdr1 N86Y– Year 2022	20](#_Toc219846320)

[2.14.	Forest Plot Pfmdr1 N86Y– Year 2023	20](#_Toc219846321)

[3.	Pfmdr1 Y184F	21](#_Toc219846322)

[3.1.	Forest Plot Pfmdr1 Y184F– Year 2010	21](#_Toc219846323)

[3.2.	Forest Plot Pfmdr1 Y184F– Year 2011	21](#_Toc219846324)

[3.3.	Forest Plot Pfmdr1 Y184F– Year 2012	22](#_Toc219846325)

[3.4.	Forest Plot Pfmdr1 Y184F– Year 2013	22](#_Toc219846326)

[3.5.	Forest Plot Pfmdr1 Y184F– Year 2014	23](#_Toc219846327)

[3.6.	Forest Plot Pfmdr1 Y184F– Year 2015	23](#_Toc219846328)

[3.7.	Forest Plot Pfmdr1 Y184F– Year 2016	24](#_Toc219846329)

[3.8.	Forest Plot Pfmdr1 Y184F– Year 2017	25](#_Toc219846330)

[3.9.	Forest Plot Pfmdr1 Y184F– Year 2018	26](#_Toc219846331)

[3.10.	Forest Plot Pfmdr1 Y184F– Year 2019	27](#_Toc219846332)

[3.11.	Forest Plot Pfmdr1 Y184F– Year 2020	27](#_Toc219846333)

[3.12.	Forest Plot Pfmdr1 Y184F– Year 2021	28](#_Toc219846334)

[3.13.	Forest Plot Pfmdr1 Y184F– Year 2022	28](#_Toc219846335)

[3.14.	Forest Plot Pfmdr1 Y184F– Year 2023	28](#_Toc219846336)

[4.	Pfmdr1 NFD	29](#_Toc219846337)

[4.1.	Forest Plot Pfmdr1 NFD– Year 2010	29](#_Toc219846338)

[4.2.	Forest Plot Pfmdr1 NFD– Year 2011	29](#_Toc219846339)

[4.3.	Forest Plot Pfmdr1 NFD– Year 2012	30](#_Toc219846340)

[4.4.	Forest Plot Pfmdr1 NFD– Year 2013	30](#_Toc219846341)

[4.5.	Forest Plot Pfmdr1 NFD– Year 2014	30](#_Toc219846342)

[4.6.	Forest Plot Pfmdr1 NFD– Year 2015	31](#_Toc219846343)

[4.7.	Forest Plot Pfmdr1 NFD– Year 2016	31](#_Toc219846344)

[4.8.	Forest Plot Pfmdr1 NFD– Year 2017	32](#_Toc219846345)

[4.9.	Forest Plot Pfmdr1 NFD– Year 2018	32](#_Toc219846346)

[4.10.	Forest Plot Pfmdr1 NFD– Year 2019	33](#_Toc219846347)

[4.11.	Forest Plot Pfmdr1 NFD– Year 2020	33](#_Toc219846348)

[4.12.	Forest Plot Pfmdr1 NFD– Year 2021	33](#_Toc219846349)

[4.13.	Forest Plot Pfmdr1 NFD– Year 2022	34](#_Toc219846350)

[4.14.	Forest Plot Pfmdr1 NFD– Year 2023	34](#_Toc219846351)

# 1. Pfcrt K76T

## 1.1. Forest Plot Pfcrt K76T– Year 2010

## 1.2. Forest Plot Pfcrt K76T– Year 2011

## 1.3. Forest Plot Pfcrt K76T– Year 2012

## 1.4. Forest Plot Pfcrt K76T– Year 2013

## 1.5. Forest Plot Pfcrt K76T– Year 2014

## 1.6. Forest Plot Pfcrt K76T– Year 2015

## 1.7. Forest Plot Pfcrt K76T– Year 2016

## 1.8. Forest Plot Pfcrt K76T– Year 2017

## 1.9. Forest Plot Pfcrt K76T– Year 2018

## 1.10. Forest Plot Pfcrt K76T– Year 2019

## 1.11. Forest Plot Pfcrt K76T– Year 2020

## 1.12. Forest Plot Pfcrt K76T– Year 2021

## 1.13. Forest Plot Pfcrt K76T– Year 2022

## 1.14. Forest Plot Pfcrt K76T– Year 2023

# 2. Pfmdr1 N86Y

## 2.1. Forest Plot Pfmdr1 N86Y– Year 2010

## 2.2. Forest Plot Pfmdr1 N86Y– Year 2011

## 2.3. Forest Plot Pfmdr1 N86Y– Year 2012

## 2.4. Forest Plot Pfmdr1 N86Y– Year 2013

## 2.5. Forest Plot Pfmdr1 N86Y– Year 2014

## 2.6. Forest Plot Pfmdr1 N86Y– Year 2015

## 2.7. Forest Plot Pfmdr1 N86Y– Year 2016

## 2.8. Forest Plot Pfmdr1 N86Y– Year 2017

## 2.9. Forest Plot Pfmdr1 N86Y– Year 2018

## 2.10. Forest Plot Pfmdr1 N86Y– Year 2019

## 2.11. Forest Plot Pfmdr1 N86Y– Year 2020

## 2.12. Forest Plot Pfmdr1 N86Y– Year 2021

## 2.13. Forest Plot Pfmdr1 N86Y– Year 2022

## 2.14. Forest Plot Pfmdr1 N86Y– Year 2023

# 3. Pfmdr1 Y184F

## 3.1. Forest Plot Pfmdr1 Y184F– Year 2010

## 3.2. Forest Plot Pfmdr1 Y184F– Year 2011

## 3.3. Forest Plot Pfmdr1 Y184F– Year 2012

## 3.4. Forest Plot Pfmdr1 Y184F– Year 2013

## 3.5. Forest Plot Pfmdr1 Y184F– Year 2014

## 3.6. Forest Plot Pfmdr1 Y184F– Year 2015

## 3.7. Forest Plot Pfmdr1 Y184F– Year 2016

## 3.8. Forest Plot Pfmdr1 Y184F– Year 2017

## 3.9. Forest Plot Pfmdr1 Y184F– Year 2018

## 3.10. Forest Plot Pfmdr1 Y184F– Year 2019

## 3.11. Forest Plot Pfmdr1 Y184F– Year 2020

## 3.12. Forest Plot Pfmdr1 Y184F– Year 2021

## 3.13. Forest Plot Pfmdr1 Y184F– Year 2022

## 3.14. Forest Plot Pfmdr1 Y184F– Year 2023

# 4. Pfmdr1 NFD

## 4.1. Forest Plot Pfmdr1 NFD– Year 2010

## 4.2. Forest Plot Pfmdr1 NFD– Year 2011

## 4.3. Forest Plot Pfmdr1 NFD– Year 2012

## 4.4. Forest Plot Pfmdr1 NFD– Year 2013

## 4.5. Forest Plot Pfmdr1 NFD– Year 2014

## 4.6. Forest Plot Pfmdr1 NFD– Year 2015

## 4.7. Forest Plot Pfmdr1 NFD– Year 2016

## 4.8. Forest Plot Pfmdr1 NFD– Year 2017

## 4.9. Forest Plot Pfmdr1 NFD– Year 2018

## 4.10. Forest Plot Pfmdr1 NFD– Year 2019

## 4.11. Forest Plot Pfmdr1 NFD– Year 2020

## 4.12. Forest Plot Pfmdr1 NFD– Year 2021

## 4.13. Forest Plot Pfmdr1 NFD– Year 2022

## 4.14. Forest Plot Pfmdr1 NFD– Year 2023
