# Supplementary material for: Spatio-temporal trends of the prevalence of Plasmodium falciparum antimalarial drug resistance markers across sub-Saharan Africa from 2010 to 2024: a systematic review and meta-analysis
Source: BMC Infect Dis. 2026 Sep 3;26:1605. doi: 10.1186/s12879-026-14233-2 (PMC13545778; doi:10.1186/s12879-026-14233-2)
Supplement: Supplementary file 1 [file 12879_2026_14233_MOESM1_ESM.zip › Supplementary materials_15.07.2026/Supplementary_07_SP Resistance_Forest plot by year.docx]

**Supplementary document_07**

Contents

[1.	Pfdhfr N51I	5](#_Toc219843951)

[1.1.	Forest Plot Pfdhfr N51I– Year 2010	5](#_Toc219843952)

[1.2.	Forest Plot Pfdhfr N51I– Year 2011	5](#_Toc219843953)

[1.3.	Forest Plot Pfdhfr N51I– Year 2012	6](#_Toc219843954)

[1.4.	Forest Plot Pfdhfr N51I– Year 2013	6](#_Toc219843955)

[1.5.	Forest Plot Pfdhfr N51I– Year 2014	7](#_Toc219843956)

[1.6.	Forest Plot Pfdhfr N51I– Year 2015	7](#_Toc219843957)

[1.7.	Forest Plot Pfdhfr N51I– Year 2016	8](#_Toc219843958)

[1.8.	Forest Plot Pfdhfr N51I– Year 2017	9](#_Toc219843959)

[1.9.	Forest Plot Pfdhfr N51I– Year 2018	10](#_Toc219843960)

[1.10.	Forest Plot Pfdhfr N51I– Year 2019	11](#_Toc219843961)

[1.11.	Forest Plot Pfdhfr N51I– Year 2020	11](#_Toc219843962)

[1.12.	Forest Plot Pfdhfr N51I– Year 2021	12](#_Toc219843963)

[1.13.	Forest Plot Pfdhfr N51I– Year 2022	12](#_Toc219843964)

[1.14.	Forest Plot Pfdhfr N51I– Year 2023	12](#_Toc219843965)

[2.	Pfdhfr C59R	13](#_Toc219843966)

[2.1.	Forest Plot Pfdhfr C59R– Year 2010	13](#_Toc219843967)

[2.2.	Forest Plot Pfdhfr C59R– Year 2011	13](#_Toc219843968)

[2.3.	Forest Plot Pfdhfr C59R– Year 2012	14](#_Toc219843969)

[2.4.	Forest Plot Pfdhfr C59R– Year 2013	14](#_Toc219843970)

[2.5.	Forest Plot Pfdhfr C59R– Year 2014	15](#_Toc219843971)

[2.6.	Forest Plot Pfdhfr C59R– Year 2015	15](#_Toc219843972)

[2.7.	Forest Plot Pfdhfr C59R– Year 2016	16](#_Toc219843973)

[2.8.	Forest Plot Pfdhfr C59R– Year 2017	17](#_Toc219843974)

[2.9.	Forest Plot Pfdhfr C59R– Year 2018	18](#_Toc219843975)

[2.10.	Forest Plot Pfdhfr C59R– Year 2019	18](#_Toc219843976)

[2.11.	Forest Plot Pfdhfr C59R– Year 2020	19](#_Toc219843977)

[2.12.	Forest Plot Pfdhfr C59R– Year 2021	19](#_Toc219843978)

[2.13.	Forest Plot Pfdhfr C59R– Year 2022	19](#_Toc219843979)

[2.14.	Forest Plot Pfdhfr C59R– Year 2023	20](#_Toc219843980)

[3.	Pfdhfr S108N	20](#_Toc219843981)

[3.1.	Forest Plot Pfdhfr S108N– Year 2010	20](#_Toc219843982)

[3.2.	Forest Plot Pfdhfr S108N– Year 2011	21](#_Toc219843983)

[3.3.	Forest Plot Pfdhfr S108N– Year 2012	21](#_Toc219843984)

[3.4.	Forest Plot Pfdhfr S108N– Year 2013	22](#_Toc219843985)

[3.5.	Forest Plot Pfdhfr S108N– Year 2014	22](#_Toc219843986)

[3.6.	Forest Plot Pfdhfr S108N– Year 2015	23](#_Toc219843987)

[3.7.	Forest Plot Pfdhfr S108N– Year 2016	23](#_Toc219843988)

[3.8.	Forest Plot Pfdhfr S108N– Year 2017	24](#_Toc219843989)

[3.9.	Forest Plot Pfdhfr S108N– Year 2018	25](#_Toc219843990)

[3.10.	Forest Plot Pfdhfr S108N– Year 2019	26](#_Toc219843991)

[3.11.	Forest Plot Pfdhfr S108N– Year 2020	26](#_Toc219843992)

[3.12.	Forest Plot Pfdhfr S108N– Year 2021	27](#_Toc219843993)

[3.13.	Forest Plot Pfdhfr S108N– Year 2022	27](#_Toc219843994)

[3.14.	Forest Plot Pfdhfr S108N– Year 2023	27](#_Toc219843995)

[4.	Pfdhfr I164L	28](#_Toc219843996)

[4.1.	Forest Plot Pfdhfr I164L– Year 2010	28](#_Toc219843997)

[4.2.	Forest Plot Pfdhfr I164L– Year 2011	28](#_Toc219843998)

[4.3.	Forest Plot Pfdhfr I164L– Year 2012	29](#_Toc219843999)

[4.4.	Forest Plot Pfdhfr I164L– Year 2013	29](#_Toc219844000)

[4.5.	Forest Plot Pfdhfr I164L– Year 2014	30](#_Toc219844001)

[4.6.	Forest Plot Pfdhfr I164L– Year 2015	30](#_Toc219844002)

[4.7.	Forest Plot Pfdhfr I164L– Year 2016	31](#_Toc219844003)

[4.8.	Forest Plot Pfdhfr I164L– Year 2017	31](#_Toc219844004)

[4.9.	Forest Plot Pfdhfr I164L– Year 2018	32](#_Toc219844005)

[4.10.	Forest Plot Pfdhfr I164L– Year 2019	32](#_Toc219844006)

[4.11.	Forest Plot Pfdhfr I164L– Year 2020	33](#_Toc219844007)

[4.12.	Forest Plot Pfdhfr I164L– Year 2021	33](#_Toc219844008)

[4.13.	Forest Plot Pfdhfr I164L– Year 2022	33](#_Toc219844009)

[4.14.	Forest Plot Pfdhfr I164L– Year 2023	34](#_Toc219844010)

[5.	Pfdhps I431V	34](#_Toc219844011)

[5.1.	Forest Plot Pfdhps I431V– Year 2010	34](#_Toc219844012)

[5.2.	Forest Plot Pfdhps I431V– Year 2011	34](#_Toc219844013)

[5.3.	Forest Plot Pfdhps I431V– Year 2012	34](#_Toc219844014)

[5.4.	Forest Plot Pfdhps I431V– Year 2014	35](#_Toc219844015)

[5.5.	Forest Plot Pfdhps I431V– Year 2015	35](#_Toc219844016)

[5.6.	Forest Plot Pfdhps I431V– Year 2016	35](#_Toc219844017)

[5.7.	Forest Plot Pfdhps I431V– Year 2017	36](#_Toc219844018)

[5.8.	Forest Plot Pfdhps I431V– Year 2018	36](#_Toc219844019)

[5.9.	Forest Plot Pfdhps I431V– Year 2019	37](#_Toc219844020)

[5.10.	Forest Plot Pfdhps I431V– Year 2020	37](#_Toc219844021)

[5.11.	Forest Plot Pfdhps I431V– Year 2021	37](#_Toc219844022)

[5.12.	Forest Plot Pfdhps I431V– Year 2022	38](#_Toc219844023)

[5.13.	Forest Plot Pfdhps I431V– Year 2023	38](#_Toc219844024)

[6.	Pfdhps S436A	38](#_Toc219844025)

[6.1.	Forest Plot Pfdhps S436A– Year 2010	38](#_Toc219844026)

[6.2.	Forest Plot Pfdhps S436A– Year 2011	39](#_Toc219844027)

[6.3.	Forest Plot Pfdhps S436A– Year 2012	39](#_Toc219844028)

[6.4.	Forest Plot Pfdhps S436A– Year 2013	40](#_Toc219844029)

[6.5.	Forest Plot Pfdhps S436A– Year 2014	40](#_Toc219844030)

[6.6.	Forest Plot Pfdhps S436A– Year 2015	41](#_Toc219844031)

[6.7.	Forest Plot Pfdhps S436A– Year 2016	41](#_Toc219844032)

[6.8.	Forest Plot Pfdhps S436A– Year 2017	42](#_Toc219844033)

[6.9.	Forest Plot Pfdhps S436A– Year 2018	43](#_Toc219844034)

[6.10.	Forest Plot Pfdhps S436A– Year 2019	43](#_Toc219844035)

[6.11.	Forest Plot Pfdhps S436A– Year 2020	44](#_Toc219844036)

[6.12.	Forest Plot Pfdhps S436A– Year 2021	44](#_Toc219844037)

[6.13.	Forest Plot Pfdhps S436A– Year 2022	44](#_Toc219844038)

[6.14.	Forest Plot Pfdhps S436A– Year 2023	45](#_Toc219844039)

[7.	Pfdhps A437G	45](#_Toc219844040)

[7.1.	Forest Plot Pfdhps A437G– Year 2010	45](#_Toc219844041)

[7.2.	Forest Plot Pfdhps A437G– Year 2011	46](#_Toc219844042)

[7.3.	Forest Plot Pfdhps A437G– Year 2012	46](#_Toc219844043)

[7.4.	Forest Plot Pfdhps A437G– Year 2013	47](#_Toc219844044)

[7.5.	Forest Plot Pfdhps A437G– Year 2014	47](#_Toc219844045)

[7.6.	Forest Plot Pfdhps A437G– Year 2015	48](#_Toc219844046)

[7.7.	Forest Plot Pfdhps A437G– Year 2016	49](#_Toc219844047)

[7.8.	Forest Plot Pfdhps A437G– Year 2017	50](#_Toc219844048)

[7.9.	Forest Plot Pfdhps A437G– Year 2018	51](#_Toc219844049)

[7.10.	Forest Plot Pfdhps A437G– Year 2019	52](#_Toc219844050)

[7.11.	Forest Plot Pfdhps A437G– Year 2020	52](#_Toc219844051)

[7.12.	Forest Plot Pfdhps A437G– Year 2021	53](#_Toc219844052)

[7.13.	Forest Plot Pfdhps A437G– Year 2022	53](#_Toc219844053)

[7.14.	Forest Plot Pfdhps A437G– Year 2023	53](#_Toc219844054)

[8.	Pfdhps K540E	54](#_Toc219844055)

[8.1.	Forest Plot Pfdhps K540E– Year 2010	54](#_Toc219844056)

[8.2.	Forest Plot Pfdhps K540E– Year 2011	54](#_Toc219844057)

[8.3.	Forest Plot Pfdhps K540E– Year 2012	55](#_Toc219844058)

[8.4.	Forest Plot Pfdhps K540E– Year 2013	55](#_Toc219844059)

[8.5.	Forest Plot Pfdhps K540E– Year 2014	56](#_Toc219844060)

[8.6.	Forest Plot Pfdhps K540E– Year 2015	56](#_Toc219844061)

[8.7.	Forest Plot Pfdhps K540E– Year 2016	57](#_Toc219844062)

[8.8.	Forest Plot Pfdhps K540E– Year 2017	58](#_Toc219844063)

[8.9.	Forest Plot Pfdhps K540E– Year 2018	59](#_Toc219844064)

[8.10.	Forest Plot Pfdhps K540E– Year 2019	60](#_Toc219844065)

[8.11.	Forest Plot Pfdhps K540E– Year 2020	60](#_Toc219844066)

[8.12.	Forest Plot Pfdhps K540E– Year 2021	61](#_Toc219844067)

[8.13.	Forest Plot Pfdhps K540E– Year 2022	61](#_Toc219844068)

[8.14.	Forest Plot Pfdhps K540E– Year 2023	61](#_Toc219844069)

[9.	Pfdhps A581G	62](#_Toc219844070)

[9.1.	Forest Plot Pfdhps A581G– Year 2010	62](#_Toc219844071)

[9.2.	Forest Plot Pfdhps A581G– Year 2011	62](#_Toc219844072)

[9.3.	Forest Plot Pfdhps A581G– Year 2012	63](#_Toc219844073)

[9.4.	Forest Plot Pfdhps A581G– Year 2013	63](#_Toc219844074)

[9.5.	Forest Plot Pfdhps A581G– Year 2014	64](#_Toc219844075)

[9.6.	Forest Plot Pfdhps A581G– Year 2015	64](#_Toc219844076)

[9.7.	Forest Plot Pfdhps A581G– Year 2016	65](#_Toc219844077)

[9.8.	Forest Plot Pfdhps A581G– Year 2017	66](#_Toc219844078)

[9.9.	Forest Plot Pfdhps A581G– Year 2018	67](#_Toc219844079)

[9.10.	Forest Plot Pfdhps A581G– Year 2019	68](#_Toc219844080)

[9.11.	Forest Plot Pfdhps A581G– Year 2020	68](#_Toc219844081)

[9.12.	Forest Plot Pfdhps A581G– Year 2021	69](#_Toc219844082)

[9.13.	Forest Plot Pfdhps A581G– Year 2022	69](#_Toc219844083)

[9.14.	Forest Plot Pfdhps A581G– Year 2023	69](#_Toc219844084)

# 1. Pfdhfr N51I

## 1.1. Forest Plot Pfdhfr N51I– Year 2010

## 1.2. Forest Plot Pfdhfr N51I– Year 2011

## 1.3. Forest Plot Pfdhfr N51I– Year 2012

## 1.4. Forest Plot Pfdhfr N51I– Year 2013

## 1.5. Forest Plot Pfdhfr N51I– Year 2014

## 1.6. Forest Plot Pfdhfr N51I– Year 2015

## 1.7. Forest Plot Pfdhfr N51I– Year 2016

## 1.8. Forest Plot Pfdhfr N51I– Year 2017

## 1.9. Forest Plot Pfdhfr N51I– Year 2018

## 1.10. Forest Plot Pfdhfr N51I– Year 2019

## 1.11. Forest Plot Pfdhfr N51I– Year 2020

## 1.12. Forest Plot Pfdhfr N51I– Year 2021

## 1.13. Forest Plot Pfdhfr N51I– Year 2022

## 1.14. Forest Plot Pfdhfr N51I– Year 2023

# 2. Pfdhfr C59R

## 2.1. Forest Plot Pfdhfr C59R– Year 2010

## 2.2. Forest Plot Pfdhfr C59R– Year 2011

## 2.3. Forest Plot Pfdhfr C59R– Year 2012

## 2.4. Forest Plot Pfdhfr C59R– Year 2013

## 2.5. Forest Plot Pfdhfr C59R– Year 2014

## 2.6. Forest Plot Pfdhfr C59R– Year 2015

## 2.7. Forest Plot Pfdhfr C59R– Year 2016

## 2.8. Forest Plot Pfdhfr C59R– Year 2017

## 2.9. Forest Plot Pfdhfr C59R– Year 2018

## 2.10. Forest Plot Pfdhfr C59R– Year 2019

## 2.11. Forest Plot Pfdhfr C59R– Year 2020

## 2.12. Forest Plot Pfdhfr C59R– Year 2021

## 2.13. Forest Plot Pfdhfr C59R– Year 2022

## 2.14. Forest Plot Pfdhfr C59R– Year 2023

# 3. Pfdhfr S108N

## 3.1. Forest Plot Pfdhfr S108N– Year 2010

## 3.2. Forest Plot Pfdhfr S108N– Year 2011

## 3.3. Forest Plot Pfdhfr S108N– Year 2012

## 3.4. Forest Plot Pfdhfr S108N– Year 2013

## 3.5. Forest Plot Pfdhfr S108N– Year 2014

## 3.6. Forest Plot Pfdhfr S108N– Year 2015

## 3.7. Forest Plot Pfdhfr S108N– Year 2016

## 3.8. Forest Plot Pfdhfr S108N– Year 2017

## 3.9. Forest Plot Pfdhfr S108N– Year 2018

## 3.10. Forest Plot Pfdhfr S108N– Year 2019

## 3.11. Forest Plot Pfdhfr S108N– Year 2020

## 3.12. Forest Plot Pfdhfr S108N– Year 2021

## 3.13. Forest Plot Pfdhfr S108N– Year 2022

## 3.14. Forest Plot Pfdhfr S108N– Year 2023

# 4. Pfdhfr I164L

## 4.1. Forest Plot Pfdhfr I164L– Year 2010

## 4.2. Forest Plot Pfdhfr I164L– Year 2011

## 4.3. Forest Plot Pfdhfr I164L– Year 2012

## 4.4. Forest Plot Pfdhfr I164L– Year 2013

## 4.5. Forest Plot Pfdhfr I164L– Year 2014

## 4.6. Forest Plot Pfdhfr I164L– Year 2015

## 4.7. Forest Plot Pfdhfr I164L– Year 2016

## 4.8. Forest Plot Pfdhfr I164L– Year 2017

## 4.9. Forest Plot Pfdhfr I164L– Year 2018

## 4.10. Forest Plot Pfdhfr I164L– Year 2019

## 4.11. Forest Plot Pfdhfr I164L– Year 2020

## 4.12. Forest Plot Pfdhfr I164L– Year 2021

## 4.13. Forest Plot Pfdhfr I164L– Year 2022

## 4.14. Forest Plot Pfdhfr I164L– Year 2023

# 5. Pfdhps I431V

## 5.1. Forest Plot Pfdhps I431V– Year 2010

## 5.2. Forest Plot Pfdhps I431V– Year 2011

## 5.3. Forest Plot Pfdhps I431V– Year 2012

## 5.4. Forest Plot Pfdhps I431V– Year 2014

## 5.5. Forest Plot Pfdhps I431V– Year 2015

## 5.6. Forest Plot Pfdhps I431V– Year 2016

## 5.7. Forest Plot Pfdhps I431V– Year 2017

## 5.8. Forest Plot Pfdhps I431V– Year 2018

## 5.9. Forest Plot Pfdhps I431V– Year 2019

## 5.10. Forest Plot Pfdhps I431V– Year 2020

## 5.11. Forest Plot Pfdhps I431V– Year 2021

## 5.12. Forest Plot Pfdhps I431V– Year 2022

## 5.13. Forest Plot Pfdhps I431V– Year 2023

# 6. Pfdhps S436A

## 6.1. Forest Plot Pfdhps S436A– Year 2010

## 6.2. Forest Plot Pfdhps S436A– Year 2011

## 6.3. Forest Plot Pfdhps S436A– Year 2012

## 6.4. Forest Plot Pfdhps S436A– Year 2013

## 6.5. Forest Plot Pfdhps S436A– Year 2014

## 6.6. Forest Plot Pfdhps S436A– Year 2015

## 6.7. Forest Plot Pfdhps S436A– Year 2016

## 6.8. Forest Plot Pfdhps S436A– Year 2017

## 6.9. Forest Plot Pfdhps S436A– Year 2018

## 6.10. Forest Plot Pfdhps S436A– Year 2019

## 6.11. Forest Plot Pfdhps S436A– Year 2020

## 6.12. Forest Plot Pfdhps S436A– Year 2021

## 6.13. Forest Plot Pfdhps S436A– Year 2022

## 6.14. Forest Plot Pfdhps S436A– Year 2023

# 7. Pfdhps A437G

## 7.1. Forest Plot Pfdhps A437G– Year 2010

## 7.2. Forest Plot Pfdhps A437G– Year 2011

## 7.3. Forest Plot Pfdhps A437G– Year 2012

## 7.4. Forest Plot Pfdhps A437G– Year 2013

## 7.5. Forest Plot Pfdhps A437G– Year 2014

## 7.6. Forest Plot Pfdhps A437G– Year 2015

## 7.7. Forest Plot Pfdhps A437G– Year 2016

## 7.8. Forest Plot Pfdhps A437G– Year 2017

## 7.9. Forest Plot Pfdhps A437G– Year 2018

## 7.10. Forest Plot Pfdhps A437G– Year 2019

## 7.11. Forest Plot Pfdhps A437G– Year 2020

## 7.12. Forest Plot Pfdhps A437G– Year 2021

## 7.13. Forest Plot Pfdhps A437G– Year 2022

## 7.14. Forest Plot Pfdhps A437G– Year 2023

# 8. Pfdhps K540E

## 8.1. Forest Plot Pfdhps K540E– Year 2010

## 8.2. Forest Plot Pfdhps K540E– Year 2011

## 8.3. Forest Plot Pfdhps K540E– Year 2012

## 8.4. Forest Plot Pfdhps K540E– Year 2013

## 8.5. Forest Plot Pfdhps K540E– Year 2014

## 8.6. Forest Plot Pfdhps K540E– Year 2015

## 8.7. Forest Plot Pfdhps K540E– Year 2016

## 8.8. Forest Plot Pfdhps K540E– Year 2017

## 8.9. Forest Plot Pfdhps K540E– Year 2018

## 8.10. Forest Plot Pfdhps K540E– Year 2019

## 8.11. Forest Plot Pfdhps K540E– Year 2020

## 8.12. Forest Plot Pfdhps K540E– Year 2021

## 8.13. Forest Plot Pfdhps K540E– Year 2022

## 8.14. Forest Plot Pfdhps K540E– Year 2023

# 9. Pfdhps A581G

## 9.1. Forest Plot Pfdhps A581G– Year 2010

## 9.2. Forest Plot Pfdhps A581G– Year 2011

## 9.3. Forest Plot Pfdhps A581G– Year 2012

## 9.4. Forest Plot Pfdhps A581G– Year 2013

## 9.5. Forest Plot Pfdhps A581G– Year 2014

## 9.6. Forest Plot Pfdhps A581G– Year 2015

## 9.7. Forest Plot Pfdhps A581G– Year 2016

## 9.8. Forest Plot Pfdhps A581G– Year 2017

## 9.9. Forest Plot Pfdhps A581G– Year 2018

## 9.10. Forest Plot Pfdhps A581G– Year 2019

## 9.11. Forest Plot Pfdhps A581G– Year 2020

## 9.12. Forest Plot Pfdhps A581G– Year 2021

## 9.13. Forest Plot Pfdhps A581G– Year 2022

## 9.14. Forest Plot Pfdhps A581G– Year 2023
