# Supplementary material for: Spatio-temporal trends of the prevalence of Plasmodium falciparum antimalarial drug resistance markers across sub-Saharan Africa from 2010 to 2024: a systematic review and meta-analysis
Source: BMC Infect Dis. 2026 Sep 3;26:1605. doi: 10.1186/s12879-026-14233-2 (PMC13545778; doi:10.1186/s12879-026-14233-2)
Supplement: Supplementary file 1 [file 12879_2026_14233_MOESM1_ESM.zip › Supplementary materials_15.07.2026/Supplementary_08_SP resistance_Forest plot by African Region and by year.docx]

**Supplementary:** Forest plot of Sulfadoxine-Pyrimethamine resistance by sub-Saharan African region (Eastern, Central, Northern, Southern, and Western) per year

Table of Contents

[*1.*	*Pfdhfr I164L*	6](#_Toc234964555)

[1.1.	*Pfdhfr I164L*_Eastern Africa Region	6](#_Toc234964556)

[1.1.1.	*Pfdhfr I164L*_Eastern – Year 2010	6](#_Toc234964557)

[1.1.2.	*Pfdhfr I164L*_Eastern – Year 2011	6](#_Toc234964558)

[1.1.3.	*Pfdhfr I164L*_Eastern – Year 2012	7](#_Toc234964559)

[1.1.4.	*Pfdhfr I164L*_Eastern – Year 2013	7](#_Toc234964560)

[1.1.5.	*Pfdhfr I164L*_Eastern – Year 2014	7](#_Toc234964561)

[1.1.6.	*Pfdhfr I164L*_Eastern – Year 2015	8](#_Toc234964562)

[1.1.7.	*Pfdhfr I164L*_Eastern – Year 2016	8](#_Toc234964563)

[1.1.8.	*Pfdhfr I164L*_Eastern – Year 2017	8](#_Toc234964564)

[1.1.9.	*Pfdhfr I164L*_Eastern – Year 2018	9](#_Toc234964565)

[1.1.10.	*Pfdhfr I164L*_Eastern – Year 2019	9](#_Toc234964566)

[1.1.11.	*Pfdhfr I164L*_Eastern – Year 2021	9](#_Toc234964567)

[1.1.12.	*Pfdhfr I164L*_Eastern – Year 2023	10](#_Toc234964568)

[1.2.	*Pfdhfr I164L* _ Central Africa Region	10](#_Toc234964569)

[1.2.1.	*Pfdhfr I164L* _ Central – Year 2011	10](#_Toc234964570)

[1.2.2.	*Pfdhfr I164L* _ Central – Year 2013	10](#_Toc234964571)

[1.2.3.	*Pfdhfr I164L* _ Central – Year 2014	11](#_Toc234964572)

[1.2.4.	*Pfdhfr I164L* _ Central – Year 2015	11](#_Toc234964573)

[1.2.5.	*Pfdhfr I164L* _ Central – Year 2016	11](#_Toc234964574)

[1.2.6.	*Pfdhfr I164L* _ Central – Year 2017	12](#_Toc234964575)

[1.2.7.	*Pfdhfr I164L* _ Central – Year 2018	12](#_Toc234964576)

[1.2.8.	*Pfdhfr I164L* _ Central – Year 2019	12](#_Toc234964577)

[1.2.9.	*Pfdhfr I164L* _ Central – Year 2020	13](#_Toc234964578)

[1.2.10.	*Pfdhfr I164L* _ Central – Year 2021	13](#_Toc234964579)

[1.2.11.	*Pfdhfr I164L* _ Central – Year 2023	13](#_Toc234964580)

[1.3.	*Pfdhfr I164L*_Southern Africa Region	13](#_Toc234964581)

[1.3.1.	*Pfdhfr I164L*_Southern – Year 2010	13](#_Toc234964582)

[1.3.2.	*Pfdhfr I164L*_Southern – Year 2011	14](#_Toc234964583)

[1.3.3.	*Pfdhfr I164L*_Southern – Year 2012	14](#_Toc234964584)

[1.3.4.	*Pfdhfr I164L*_Southern – Year 2018	14](#_Toc234964585)

[1.4.	*Pfdhfr I164L*_Western Africa Region	14](#_Toc234964586)

[1.4.1.	*Pfdhfr I164L*_Western – Year 2010	14](#_Toc234964587)

[1.4.2.	*Pfdhfr I164L*_Western – Year 2011	15](#_Toc234964588)

[1.4.3.	*Pfdhfr I164L*_Western – Year 2012	15](#_Toc234964589)

[1.4.4.	*Pfdhfr I164L*_Western – Year 2013	15](#_Toc234964590)

[1.4.5.	*Pfdhfr I164L*_Western – Year 2014	16](#_Toc234964591)

[1.4.6.	*Pfdhfr I164L*_Western – Year 2015	16](#_Toc234964592)

[1.4.7.	*Pfdhfr I164L*_Western – Year 2016	16](#_Toc234964593)

[1.4.8.	*Pfdhfr I164L*_Western – Year 2017	17](#_Toc234964594)

[1.4.9.	*Pfdhfr I164L*_Western – Year 2018	17](#_Toc234964595)

[1.4.10.	*Pfdhfr I164L*_Western – Year 2019	17](#_Toc234964596)

[1.4.11.	*Pfdhfr I164L*_Western – Year 2020	18](#_Toc234964597)

[1.4.12.	*Pfdhfr I164L*_Western – Year 2021	18](#_Toc234964598)

[1.4.13.	*Pfdhfr I164L*_Western – Year 2022	18](#_Toc234964599)

[*2.*	*Pfdhps I431V*	19](#_Toc234964600)

[2.1.	*Pfdhps I431V*_ Eastern Africa Region	19](#_Toc234964601)

[2.1.1.	*Pfdhps I431V*_ Eastern – Year 2015	19](#_Toc234964602)

[2.2.	*Pfdhps I431V*_ Central Africa Region	19](#_Toc234964603)

[2.2.1.	*Pfdhps I431V*_ Central – Year 2011	19](#_Toc234964604)

[2.2.2.	*Pfdhps I431V*_ Central – Year 2014	19](#_Toc234964605)

[2.2.3.	*Pfdhps I431V*_ Central – Year 2015	19](#_Toc234964606)

[2.2.4.	*Pfdhps I431V*_ Central – Year 2016	20](#_Toc234964607)

[2.2.5.	*Pfdhps I431V*_ Central – Year 2017	20](#_Toc234964608)

[2.2.6.	*Pfdhps I431V*_ Central – Year 2018	20](#_Toc234964609)

[2.2.7.	*Pfdhps I431V*_ Central – Year 2019	20](#_Toc234964610)

[2.2.8.	*Pfdhps I431V*_ Central – Year 2020	21](#_Toc234964611)

[2.2.9.	*Pfdhps I431V*_ Central – Year 2021	21](#_Toc234964612)

[2.3.	*Pfdhps I431V*_ Western Africa Region	21](#_Toc234964613)

[2.3.1.	*Pfdhps I431V*_ Western – Year 2010	21](#_Toc234964614)

[2.3.2.	*Pfdhps I431V*_ Western – Year 2011	21](#_Toc234964615)

[2.3.3.	*Pfdhps I431V*_ Western – Year 2012	22](#_Toc234964616)

[2.3.4.	*Pfdhps I431V*_ Western – Year 2014	22](#_Toc234964617)

[2.3.5.	*Pfdhps I431V*_ Western – Year 2015	22](#_Toc234964618)

[2.3.6.	*Pfdhps I431V*_ Western – Year 2016	22](#_Toc234964619)

[2.3.7.	*Pfdhps I431V*_ Western – Year 2017	23](#_Toc234964620)

[2.3.8.	*Pfdhps I431V*_ Western – Year 2018	23](#_Toc234964621)

[2.3.9.	*Pfdhps I431V*_ Western – Year 2019	23](#_Toc234964622)

[2.3.10.	*Pfdhps I431V*_ Western – Year 2020	24](#_Toc234964623)

[2.3.11.	*Pfdhps I431V*_ Western – Year 2021	24](#_Toc234964624)

[2.3.12.	*Pfdhps I431V*_ Western – Year 2022	24](#_Toc234964625)

[2.3.13.	*Pfdhps I431V*_ Western – Year 2023	24](#_Toc234964626)

[*3.*	*Pfdhps K540E*	25](#_Toc234964627)

[3.1.	*Pfdhps K540E* _ Eastern Africa Region	25](#_Toc234964628)

[3.1.1.	*Pfdhps K540E* _ Eastern – Year 2010	25](#_Toc234964629)

[3.1.2.	*Pfdhps K540E* _ Eastern – Year 2011	25](#_Toc234964630)

[3.1.3.	*Pfdhps K540E* _ Eastern – Year 2012	25](#_Toc234964631)

[3.1.4.	*Pfdhps K540E* _ Eastern – Year 2013	26](#_Toc234964632)

[3.1.5.	*Pfdhps K540E* _ Eastern – Year 2014	26](#_Toc234964633)

[3.1.6.	*Pfdhps K540E* _ Eastern – Year 2015	26](#_Toc234964634)

[3.1.7.	*Pfdhps K540E* _ Eastern – Year 2016	27](#_Toc234964635)

[3.1.8.	*Pfdhps K540E* _ Eastern – Year 2017	27](#_Toc234964636)

[3.1.9.	*Pfdhps K540E* _ Eastern – Year 2018	27](#_Toc234964637)

[3.1.10.	*Pfdhps K540E* _ Eastern – Year 2019	28](#_Toc234964638)

[3.1.11.	*Pfdhps K540E* _ Eastern – Year 2021	28](#_Toc234964639)

[3.1.12.	*Pfdhps K540E* _ Eastern – Year 2023	28](#_Toc234964640)

[3.2.	*Pfdhps K540E* _ Central Africa Region	29](#_Toc234964641)

[3.2.1.	*Pfdhps K540E* _ Central – Year 2011	29](#_Toc234964642)

[3.2.2.	*Pfdhps K540E* _ Central – Year 2012	29](#_Toc234964643)

[3.2.3.	*Pfdhps K540E* _ Central – Year 2013	29](#_Toc234964644)

[3.2.4.	*Pfdhps K540E* _ Central – Year 2014	29](#_Toc234964645)

[3.2.5.	*Pfdhps K540E* _ Central – Year 2015	30](#_Toc234964646)

[3.2.6.	*Pfdhps K540E* _ Central – Year 2016	30](#_Toc234964647)

[3.2.7.	*Pfdhps K540E* _ Central – Year 2017	30](#_Toc234964648)

[3.2.8.	*Pfdhps K540E* _ Central – Year 2018	31](#_Toc234964649)

[3.2.9.	*Pfdhps K540E* _ Central – Year 2019	31](#_Toc234964650)

[3.2.10.	*Pfdhps K540E* _ Central – Year 2020	31](#_Toc234964651)

[3.2.11.	*Pfdhps K540E* _ Central – Year 2021	32](#_Toc234964652)

[3.2.12.	*Pfdhps K540E* _ Central – Year 2023	32](#_Toc234964653)

[3.3.	*Pfdhps K540E* _ Northern Africa Region	32](#_Toc234964654)

[3.3.1.	*Pfdhps K540E* _ Northern – Year 2012	32](#_Toc234964655)

[3.3.2.	*Pfdhps K540E* _ Northern – Year 2016	32](#_Toc234964656)

[3.3.3.	*Pfdhps K540E* _ Northern – Year 2017	33](#_Toc234964657)

[3.3.4.	*Pfdhps K540E* _ Northern – Year 2018	33](#_Toc234964658)

[3.4.	*Pfdhps K540E* _ Southern Africa Region	33](#_Toc234964659)

[3.4.1.	*Pfdhps K540E* _ Southern – Year 2010	33](#_Toc234964660)

[3.4.2.	*Pfdhps K540E* _ Southern – Year 2011	33](#_Toc234964661)

[3.4.3.	*Pfdhps K540E* _ Southern – Year 2012	33](#_Toc234964662)

[3.4.4.	*Pfdhps K540E* _ Southern – Year 2018	34](#_Toc234964663)

[3.5.	*Pfdhps K540E* _ Western Africa Region	34](#_Toc234964664)

[3.5.1.	*Pfdhps K540E* _ Western – Year 2010	34](#_Toc234964665)

[3.5.2.	*Pfdhps K540E* _ Western – Year 2011	34](#_Toc234964666)

[3.5.3.	*Pfdhps K540E* _ Western – Year 2012	35](#_Toc234964667)

[3.5.4.	*Pfdhps K540E* _ Western – Year 2013	35](#_Toc234964668)

[3.5.5.	*Pfdhps K540E* _ Western – Year 2014	35](#_Toc234964669)

[3.5.6.	*Pfdhps K540E* _ Western – Year 2015	36](#_Toc234964670)

[3.5.7.	*Pfdhps K540E* _ Western – Year 2016	36](#_Toc234964671)

[3.5.8.	*Pfdhps K540E* _ Western – Year 2017	36](#_Toc234964672)

[3.5.9.	*Pfdhps K540E* _ Western – Year 2018	37](#_Toc234964673)

[3.5.10.	*Pfdhps K540E* _ Western – Year 2019	37](#_Toc234964674)

[3.5.11.	*Pfdhps K540E* _ Western – Year 2020	37](#_Toc234964675)

[3.5.12.	*Pfdhps K540E* _ Western – Year 2021	38](#_Toc234964676)

[3.5.13.	*Pfdhps K540E* _ Western – Year 2022	38](#_Toc234964677)

[3.5.14.	*Pfdhps K540E* _ Western – Year 2023	38](#_Toc234964678)

[*4.*	*Pfdhps A581G*	39](#_Toc234964679)

[4.1.	*Pfdhps A581G* _ Eastern Africa Region	39](#_Toc234964680)

[4.1.1.	*Pfdhps A581G* _ Eastern – Year 2010	39](#_Toc234964681)

[4.1.2.	*Pfdhps A581G* _ Eastern – Year 2011	39](#_Toc234964682)

[4.1.3.	*Pfdhps A581G* _ Eastern – Year 2012	39](#_Toc234964683)

[4.1.4.	*Pfdhps A581G* _ Eastern – Year 2013	40](#_Toc234964684)

[4.1.5.	*Pfdhps A581G* _ Eastern – Year 2014	40](#_Toc234964685)

[4.1.6.	*Pfdhps A581G* _ Eastern – Year 2015	40](#_Toc234964686)

[4.1.7.	*Pfdhps A581G* _ Eastern – Year 2016	41](#_Toc234964687)

[4.1.8.	*Pfdhps A581G* _ Eastern – Year 2017	41](#_Toc234964688)

[4.1.9.	*Pfdhps A581G* _ Eastern – Year 2018	41](#_Toc234964689)

[4.1.10.	*Pfdhps A581G* _ Eastern – Year 2019	42](#_Toc234964690)

[4.1.11.	*Pfdhps A581G* _ Eastern – Year 2021	42](#_Toc234964691)

[4.1.12.	*Pfdhps A581G* _ Eastern – Year 2023	42](#_Toc234964692)

[4.2.	*Pfdhps A581G* _ Central Africa Region	43](#_Toc234964693)

[4.2.1.	*Pfdhps A581G* _ Central – Year 2011	43](#_Toc234964694)

[4.2.2.	*Pfdhps A581G* _ Central – Year 2012	43](#_Toc234964695)

[4.2.3.	*Pfdhps A581G* _ Central – Year 2013	43](#_Toc234964696)

[4.2.4.	*Pfdhps A581G* _ Central – Year 2014	43](#_Toc234964697)

[4.2.5.	*Pfdhps A581G* _ Central – Year 2015	44](#_Toc234964698)

[4.2.6.	*Pfdhps A581G* _ Central – Year 2016	44](#_Toc234964699)

[4.2.7.	*Pfdhps A581G* _ Central – Year 2017	44](#_Toc234964700)

[4.2.8.	*Pfdhps A581G* _ Central – Year 2018	45](#_Toc234964701)

[4.2.9.	*Pfdhps A581G* _ Central – Year 2019	45](#_Toc234964702)

[4.2.10.	*Pfdhps A581G* _ Central – Year 2020	45](#_Toc234964703)

[4.2.11.	*Pfdhps A581G* _ Central – Year 2021	46](#_Toc234964704)

[4.2.12.	Central – Year 2023	46](#_Toc234964705)

[4.3.	*Pfdhps A581G* _ Northern Africa Region	46](#_Toc234964706)

[4.3.1.	*Pfdhps A581G* _ Northern – Year 2012	46](#_Toc234964707)

[4.3.2.	*Pfdhps A581G* _ Northern – Year 2016	46](#_Toc234964708)

[4.3.3.	*Pfdhps A581G* _ Northern – Year 2017	47](#_Toc234964709)

[4.4.	*Pfdhps A581G* _ Northern Africa Region	47](#_Toc234964710)

[4.4.1.	*Pfdhps A581G* _ Southern – Year 2010	47](#_Toc234964711)

[4.4.2.	*Pfdhps A581G* _ Southern – Year 2011	47](#_Toc234964712)

[4.4.3.	*Pfdhps A581G* _ Southern – Year 2012	47](#_Toc234964713)

[4.4.4.	*Pfdhps A581G* _ Southern – Year 2018	47](#_Toc234964714)

[4.5.	*Pfdhps A581G* _ Western Africa Region	48](#_Toc234964715)

[4.5.1.	*Pfdhps A581G* _ Western – Year 2010	48](#_Toc234964716)

[4.5.2.	*Pfdhps A581G* _ Western – Year 2011	48](#_Toc234964717)

[4.5.3.	*Pfdhps A581G* _ Western – Year 2012	48](#_Toc234964718)

[4.5.4.	*Pfdhps A581G* _ Western – Year 2013	49](#_Toc234964719)

[4.5.5.	*Pfdhps A581G* _ Western – Year 2014	49](#_Toc234964720)

[4.5.6.	*Pfdhps A581G* _ Western – Year 2015	49](#_Toc234964721)

[4.5.7.	*Pfdhps A581G* _ Western – Year 2016	50](#_Toc234964722)

[4.5.8.	*Pfdhps A581G* _ Western – Year 2017	50](#_Toc234964723)

[4.5.9.	*Pfdhps A581G* _ Western – Year 2018	51](#_Toc234964724)

[4.5.10.	*Pfdhps A581G* _ Western – Year 2019	51](#_Toc234964725)

[4.5.11.	*Pfdhps A581G* _ Western – Year 2020	51](#_Toc234964726)

[4.5.12.	*Pfdhps A581G* _ Western – Year 2021	52](#_Toc234964727)

[4.5.13.	*Pfdhps A581G* _ Western – Year 2022	52](#_Toc234964728)

[4.5.14.	*Pfdhps A581G* _ Western – Year 2023	52](#_Toc234964729)

# 1. *Pfdhfr I164L*

## 1.1.  *Pfdhfr I164L*_Eastern Africa Region

### 1.1.1. *Pfdhfr I164L*_Eastern – Year 2010

### 1.1.2. *Pfdhfr I164L*_Eastern – Year 2011

### 1.1.3. *Pfdhfr I164L*_Eastern – Year 2012

### 1.1.4. *Pfdhfr I164L*_Eastern – Year 2013

### 1.1.5. *Pfdhfr I164L*_Eastern – Year 2014

### 1.1.6. *Pfdhfr I164L*_Eastern – Year 2015

### 1.1.7. *Pfdhfr I164L*_Eastern – Year 2016

### 1.1.8. *Pfdhfr I164L*_Eastern – Year 2017

### 1.1.9. *Pfdhfr I164L*_Eastern – Year 2018

### 1.1.10.  *Pfdhfr I164L*_Eastern – Year 2019

### 1.1.11.  *Pfdhfr I164L*_Eastern – Year 2021

### 1.1.12.  *Pfdhfr I164L*_Eastern – Year 2023

## 1.2. *Pfdhfr I164L* _ Central Africa Region

### 1.2.1. *Pfdhfr I164L* _ Central – Year 2011

### 1.2.2. *Pfdhfr I164L* _ Central – Year 2013

### 1.2.3. *Pfdhfr I164L* _ Central – Year 2014

### 1.2.4. *Pfdhfr I164L* _ Central – Year 2015

### 1.2.5. *Pfdhfr I164L* _ Central – Year 2016

### 1.2.6. *Pfdhfr I164L* _ Central – Year 2017

### 1.2.7. *Pfdhfr I164L* _ Central – Year 2018

### 1.2.8. *Pfdhfr I164L* _ Central – Year 2019

### 1.2.9. *Pfdhfr I164L* _ Central – Year 2020

### 1.2.10.  *Pfdhfr I164L* _ Central – Year 2021

### 1.2.11.  *Pfdhfr I164L* _ Central – Year 2023

## 1.3.  *Pfdhfr I164L*_Southern Africa Region

### 1.3.1. *Pfdhfr I164L*_Southern – Year 2010

### 1.3.2. *Pfdhfr I164L*_Southern – Year 2011

### 1.3.3. *Pfdhfr I164L*_Southern – Year 2012

### 1.3.4. *Pfdhfr I164L*_Southern – Year 2018

## 1.4.  *Pfdhfr I164L*_Western Africa Region

### 1.4.1. *Pfdhfr I164L*_Western – Year 2010

### 1.4.2. *Pfdhfr I164L*_Western – Year 2011

### 1.4.3. *Pfdhfr I164L*_Western – Year 2012

### 1.4.4. *Pfdhfr I164L*_Western – Year 2013

### 1.4.5. *Pfdhfr I164L*_Western – Year 2014

### 1.4.6. *Pfdhfr I164L*_Western – Year 2015

### 1.4.7. *Pfdhfr I164L*_Western – Year 2016

### 1.4.8. *Pfdhfr I164L*_Western – Year 2017

### 1.4.9. *Pfdhfr I164L*_Western – Year 2018

### 1.4.10.  *Pfdhfr I164L*_Western – Year 2019

### 1.4.11.  *Pfdhfr I164L*_Western – Year 2020

### 1.4.12.  *Pfdhfr I164L*_Western – Year 2021

### 1.4.13.  *Pfdhfr I164L*_Western – Year 2022

# 2. *Pfdhps I431V*

## 2.1.  *Pfdhps I431V*_ Eastern Africa Region

### 2.1.1. *Pfdhps I431V*_ Eastern – Year 2015

## 2.2.  *Pfdhps I431V*_ Central Africa Region

### 2.2.1. *Pfdhps I431V*_ Central – Year 2011

### 2.2.2. *Pfdhps I431V*_ Central – Year 2014

### 2.2.3. *Pfdhps I431V*_ Central – Year 2015

### 2.2.4. *Pfdhps I431V*_ Central – Year 2016

### 2.2.5. *Pfdhps I431V*_ Central – Year 2017

### 2.2.6. *Pfdhps I431V*_ Central – Year 2018

### 2.2.7. *Pfdhps I431V*_ Central – Year 2019

### 2.2.8. *Pfdhps I431V*_ Central – Year 2020

### 2.2.9. *Pfdhps I431V*_ Central – Year 2021

## 2.3. *Pfdhps I431V*_ Western Africa Region

### 2.3.1. *Pfdhps I431V*_ Western – Year 2010

### 2.3.2. *Pfdhps I431V*_ Western – Year 2011

### 2.3.3. *Pfdhps I431V*_ Western – Year 2012

### 2.3.4. *Pfdhps I431V*_ Western – Year 2014

### 2.3.5. *Pfdhps I431V*_ Western – Year 2015

### 2.3.6. *Pfdhps I431V*_ Western – Year 2016

### 2.3.7. *Pfdhps I431V*_ Western – Year 2017

### 2.3.8. *Pfdhps I431V*_ Western – Year 2018

### 2.3.9. *Pfdhps I431V*_ Western – Year 2019

### 2.3.10.  *Pfdhps I431V*_ Western – Year 2020

### 2.3.11.  *Pfdhps I431V*_ Western – Year 2021

### 2.3.12.  *Pfdhps I431V*_ Western – Year 2022

### 2.3.13.  *Pfdhps I431V*_ Western – Year 2023

# 3. *Pfdhps K540E*

## 3.1.  *Pfdhps K540E* _ Eastern Africa Region

### 3.1.1. *Pfdhps K540E* _ Eastern – Year 2010

### 3.1.2. *Pfdhps K540E* _ Eastern – Year 2011

### 3.1.3. *Pfdhps K540E* _ Eastern – Year 2012

### 3.1.4. *Pfdhps K540E* _ Eastern – Year 2013

### 3.1.5. *Pfdhps K540E* _ Eastern – Year 2014

### 3.1.6. *Pfdhps K540E* _ Eastern – Year 2015

### 3.1.7. *Pfdhps K540E* _ Eastern – Year 2016

### 3.1.8. *Pfdhps K540E* _ Eastern – Year 2017

### 3.1.9. *Pfdhps K540E* _ Eastern – Year 2018

### 3.1.10.  *Pfdhps K540E* _ Eastern – Year 2019

### 3.1.11.  *Pfdhps K540E* _ Eastern – Year 2021

### 3.1.12.  *Pfdhps K540E* _ Eastern – Year 2023

## 3.2.  *Pfdhps K540E* _ Central Africa Region

### 3.2.1. *Pfdhps K540E* _ Central – Year 2011

### 3.2.2. *Pfdhps K540E* _ Central – Year 2012

### 3.2.3. *Pfdhps K540E* _ Central – Year 2013

### 3.2.4. *Pfdhps K540E* _ Central – Year 2014

### 3.2.5. *Pfdhps K540E* _ Central – Year 2015

### 3.2.6. *Pfdhps K540E* _ Central – Year 2016

### 3.2.7. *Pfdhps K540E* _ Central – Year 2017

### 3.2.8. *Pfdhps K540E* _ Central – Year 2018

### 3.2.9. *Pfdhps K540E* _ Central – Year 2019

### 3.2.10.  *Pfdhps K540E* _ Central – Year 2020

### 3.2.11.  *Pfdhps K540E* _ Central – Year 2021

### 3.2.12.  *Pfdhps K540E* _ Central – Year 2023

## 3.3.  *Pfdhps K540E* _ Northern Africa Region

### 3.3.1. *Pfdhps K540E* _ Northern – Year 2012

### 3.3.2. *Pfdhps K540E* _ Northern – Year 2016

### 3.3.3. *Pfdhps K540E* _ Northern – Year 2017

### 3.3.4. *Pfdhps K540E* _ Northern – Year 2018

## 3.4.  *Pfdhps K540E* _ Southern Africa Region

### 3.4.1. *Pfdhps K540E* _ Southern – Year 2010

### 3.4.2. *Pfdhps K540E* _ Southern – Year 2011

### 3.4.3. *Pfdhps K540E* _ Southern – Year 2012

### 3.4.4. *Pfdhps K540E* _ Southern – Year 2018

## 3.5.  *Pfdhps K540E* _ Western Africa Region

### 3.5.1. *Pfdhps K540E* _ Western – Year 2010

### 3.5.2. *Pfdhps K540E* _ Western – Year 2011

### 3.5.3. *Pfdhps K540E* _ Western – Year 2012

### 3.5.4. *Pfdhps K540E* _ Western – Year 2013

### 3.5.5. *Pfdhps K540E* _ Western – Year 2014

### 3.5.6. *Pfdhps K540E* _ Western – Year 2015

### 3.5.7. *Pfdhps K540E* _ Western – Year 2016

### 3.5.8. *Pfdhps K540E* _ Western – Year 2017

### 3.5.9. *Pfdhps K540E* _ Western – Year 2018

### 3.5.10.  *Pfdhps K540E* _ Western – Year 2019

### 3.5.11.  *Pfdhps K540E* _ Western – Year 2020

### 3.5.12.  *Pfdhps K540E* _ Western – Year 2021

### 3.5.13.  *Pfdhps K540E* _ Western – Year 2022

### 3.5.14.  *Pfdhps K540E* _ Western – Year 2023

# 4. *Pfdhps A581G*

## 4.1.  *Pfdhps A581G* _ Eastern Africa Region

### 4.1.1. *Pfdhps A581G* _ Eastern – Year 2010

### 4.1.2. *Pfdhps A581G* _ Eastern – Year 2011

### 4.1.3. *Pfdhps A581G* _ Eastern – Year 2012

### 4.1.4. *Pfdhps A581G* _ Eastern – Year 2013

### 4.1.5. *Pfdhps A581G* _ Eastern – Year 2014

### 4.1.6. *Pfdhps A581G* _ Eastern – Year 2015

### 4.1.7. *Pfdhps A581G* _ Eastern – Year 2016

### 4.1.8. *Pfdhps A581G* _ Eastern – Year 2017

### 4.1.9. *Pfdhps A581G* _ Eastern – Year 2018

### 4.1.10.  *Pfdhps A581G* _ Eastern – Year 2019

### 4.1.11.  *Pfdhps A581G* _ Eastern – Year 2021

### 4.1.12.  *Pfdhps A581G* _ Eastern – Year 2023

## 4.2.  *Pfdhps A581G* _ Central Africa Region

### 4.2.1. *Pfdhps A581G* _ Central – Year 2011

### 4.2.2. *Pfdhps A581G* _ Central – Year 2012

### 4.2.3. *Pfdhps A581G* _ Central – Year 2013

### 4.2.4. *Pfdhps A581G* _ Central – Year 2014

### 4.2.5. *Pfdhps A581G* _ Central – Year 2015

### 4.2.6. *Pfdhps A581G* _ Central – Year 2016

### 4.2.7. *Pfdhps A581G* _ Central – Year 2017

### 4.2.8. *Pfdhps A581G* _ Central – Year 2018

### 4.2.9. *Pfdhps A581G* _ Central – Year 2019

### 4.2.10.  *Pfdhps A581G* _ Central – Year 2020

### 4.2.11.  *Pfdhps A581G* _ Central – Year 2021

### 4.2.12.  Central – Year 2023

## 4.3.  *Pfdhps A581G* _ Northern Africa Region

### 4.3.1. *Pfdhps A581G* _ Northern – Year 2012

### 4.3.2. *Pfdhps A581G* _ Northern – Year 2016

### 4.3.3. *Pfdhps A581G* _ Northern – Year 2017

## 4.4.  *Pfdhps A581G* _ Northern Africa Region

### 4.4.1. *Pfdhps A581G* _ Southern – Year 2010

### 4.4.2. *Pfdhps A581G* _ Southern – Year 2011

### 4.4.3. *Pfdhps A581G* _ Southern – Year 2012

### 4.4.4. *Pfdhps A581G* _ Southern – Year 2018

## 4.5.  *Pfdhps A581G* _ Western Africa Region

### 4.5.1. *Pfdhps A581G* _ Western – Year 2010

### 4.5.2. *Pfdhps A581G* _ Western – Year 2011

### 4.5.3. *Pfdhps A581G* _ Western – Year 2012

### 4.5.4. *Pfdhps A581G* _ Western – Year 2013

### 4.5.5. *Pfdhps A581G* _ Western – Year 2014

### 4.5.6. *Pfdhps A581G* _ Western – Year 2015

### 4.5.7. *Pfdhps A581G* _ Western – Year 2016

### 4.5.8. *Pfdhps A581G* _ Western – Year 2017

### 4.5.9. *Pfdhps A581G* _ Western – Year 2018

### 4.5.10.  *Pfdhps A581G* _ Western – Year 2019

### 4.5.11.  *Pfdhps A581G* _ Western – Year 2020

### 4.5.12.  *Pfdhps A581G* _ Western – Year 2021

### 4.5.13.  *Pfdhps A581G* _ Western – Year 2022

### 4.5.14.  *Pfdhps A581G* _ Western – Year 2023
